# Elevating CO_2_ electrolyzer temperature accelerates the viability of chemical electrification

**DOI:** 10.1038/s44286-026-00444-6

**Published:** 2026-10-19

**Authors:** Lukas Spiekermann, Mi Gyoung Lee, Joshua Wicks, Bosi Peng, Ke Xie, Luca Bosetti, Benedikt Winter, Jan Seiler, Edward H. Sargent, André Bardow

**Affiliations:** 1 Energy and Process Systems Engineering, Department of Mechanical and Process Engineering, ETH Zurich, Zurich, Switzerland; 2 NCCR Catalysis, Zurich, Switzerland; 3 Department of Electrical and Computer Engineering, University of Toronto, Toronto, Ontario Canada; 4 Department of Materials Science and Engineering, Incheon National University, Incheon, Republic of Korea; 5 Department of Chemistry, Northwestern University, Evanston, IL USA; 6 Department of Electrical and Computer Engineering, Northwestern University, Evanston, IL USA

**Keywords:** Chemical engineering, Chemical engineering

## Abstract

Climate change mitigation has spurred interest in producing chemicals from CO_2_. CO_2_ reduction by electrochemistry can be driven directly by green electricity, yielding even multi-carbon (C2+) products. However, the resulting processes still suffer from high electrolyzer losses and substantial energy demands. This study evaluates the environmental and economic benefits of integrating optimized electrolyzers into complete processes. For CO_2_ electroreduction to ethylene and dimerization to 1-butene, elevated-temperature electrolysis effectively utilizes waste heat. Challenging the ambient-temperature paradigm, this strategy achieves system-wide savings in energy (10%), climate impacts (9%) and overall cost (11%). Generalizing these findings across various CO_2_ electroreduction pathways identifies energy savings of up to 25% and the heating system as a key design variable affecting electrolyzer design targets. Our study demonstrates how process integration can guide CO_2_ electrolyzer development toward sustainable processes.

**Figure:**
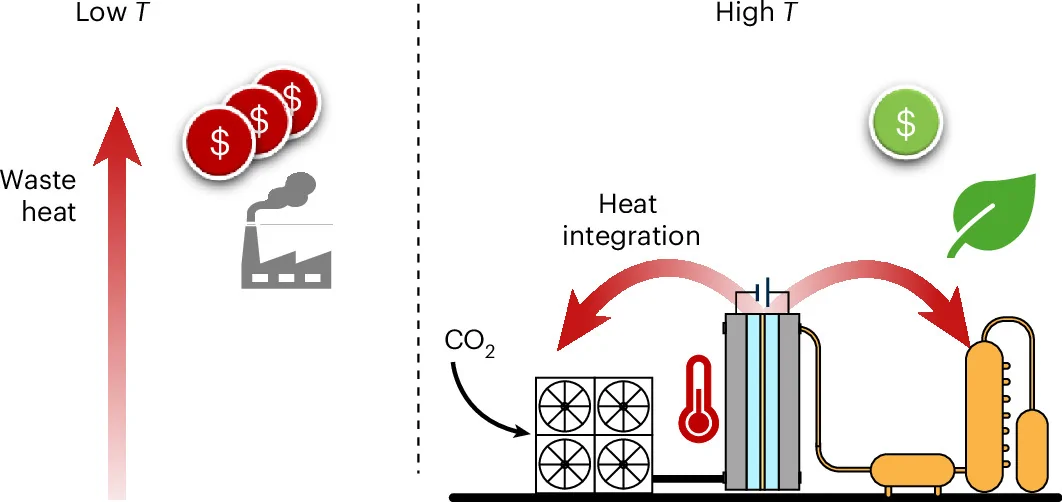

## Abstract (web summary)

Unlocking the full potential of CO_2_ electroreduction requires looking beyond ambient-temperature operation. By integrating elevated-temperature electrolysis to capture waste heat, this study highlights opportunities for substantial process-wide savings in energy, climate change impacts and cost. Ultimately, this study demonstrates how system-level integration guides the design of scalable, sustainable chemical production.

## Main

Climate change necessitates the transformation of the chemical industry away from its current fossil basis^1^. A promising alternative to fossil feedstocks is the electroreduction of CO_2_ into chemicals and fuels, driven by green electricity^2,3^. Recent years have seen substantial developments of catalysts and electrolyzers for the low-temperature CO_2_ electroreduction to products such as carbon monoxide, formic acid and ethylene^3–9^.

A challenge is CO_2_ electroreduction to larger molecules^10,11^. Typically, only C1 to C2 products are synthesized with high selectivity, but C3+ products offer a higher economic potential due to higher market prices and a higher energy density^12^. Furthermore, C3+ products can simplify downstream separation by avoiding intermediate gas separations of CO_2_, carbon monoxide, hydrogen or ethylene. Recent studies experimentally demonstrated the synthesis of C3+ products^10^, for example, butane and butenes^13,14^, but process concepts that include downstream separation are limited today.

The advance of the field relies on identifying and optimizing the key performance indicators (KPIs) for electrolyzers, such as CO_2_ conversion, Faradaic efficiency, current density and cell voltage^2^. These KPIs are often interconnected and can conflict with one another, forcing trade-offs in electrolyzer design. In addition, process performance is influenced by decisions on feedstock supply, separation, recycling of unreacted CO_2_ and process integration, inducing further usually masked trade-offs in electrolyzer design. Ultimately, the electrolyzer performance is closely tied to process KPIs, such as energy demand, cost and environmental impacts^4^.

Recent research efforts have begun to link targets for electrolyzer development to the process, calculating the impact of the electrolyzer KPIs on the overall process performance to define target values for electrolyzer KPIs, such as Faradaic efficiency or cell voltage^15,16^. Process performance is usually determined by combining electrolyzer models based on experiments^17–19^ or future scenarios^15,20–22^ with models for the downstream separation^4,15,23–37^. Most studies estimate electrical energy demands^6,22,25,33,38^, primarily for the electrolyzer. A broader viewpoint was taken by ref. ^28^, who assessed the potential of byproduct combustion and heat integration for overall energy flows in an electrolyzer and its separation system. Heat integration can improve the overall energy efficiency by more than 10% for a hydrogen production process^39^ and supply all the required heat demands for a combined CH_4_ combustion and CO_2_ electroreduction process^28^.

Still, process performance is often measured primarily by economics^2,40^. However, the mitigation potential of CO_2_ utilization technologies depends on their climate change impacts^41^. Life cycle assessment^42,43^ has thus emerged as an essential methodology for evaluating the environmental potential of electrochemical CO_2_ reduction processes^15,30,44,45^ but also needs to be linked to electrolyzer KPIs.

In summary, CO_2_ electrolyzer processes can be advanced by integrating three key factors: first, the synthesis of C2+ products to enhance economic potential; second, quantifying the cost and climate change mitigation potential on the process level of a fully integrated process; and third, closing the feedback loop from process to electrolyzer KPIs to inform electrolyzer development efforts. Addressing these challenges in new CO_2_ electrolyzer processes will accelerate the transition to sustainable chemicals, while process integration can further reduce overall climate impacts.

In this study, we quantify the impacts of the electrolyzer KPIs operating temperature, cell voltage and Faradaic efficiency on the climate change impacts and economics of a 1-butene synthesis process. This integrated tandem process concept builds on early-stage experimental work^13,14^ by combining CO_2_ electroreduction to ethylene with subsequent ethylene dimerization. 1-Butene serves as a case study for C2+ products with a wide application in polymer synthesis and current production via the cracking of fossil feedstocks^46^. Our findings highlight the benefits of higher operating temperatures for enhanced heat integration and overall process performance, while also quantifying trade-offs associated with Faradaic efficiency and cell voltage. Analyzing a variety of CO_2_ pathways establishes elevating electrolyzer temperature as a promising pathway for substantial energy savings through heat integration.

## Results

### Energy and carbon flows in the CO_2_ electroreduction process

**Fig. 1:**
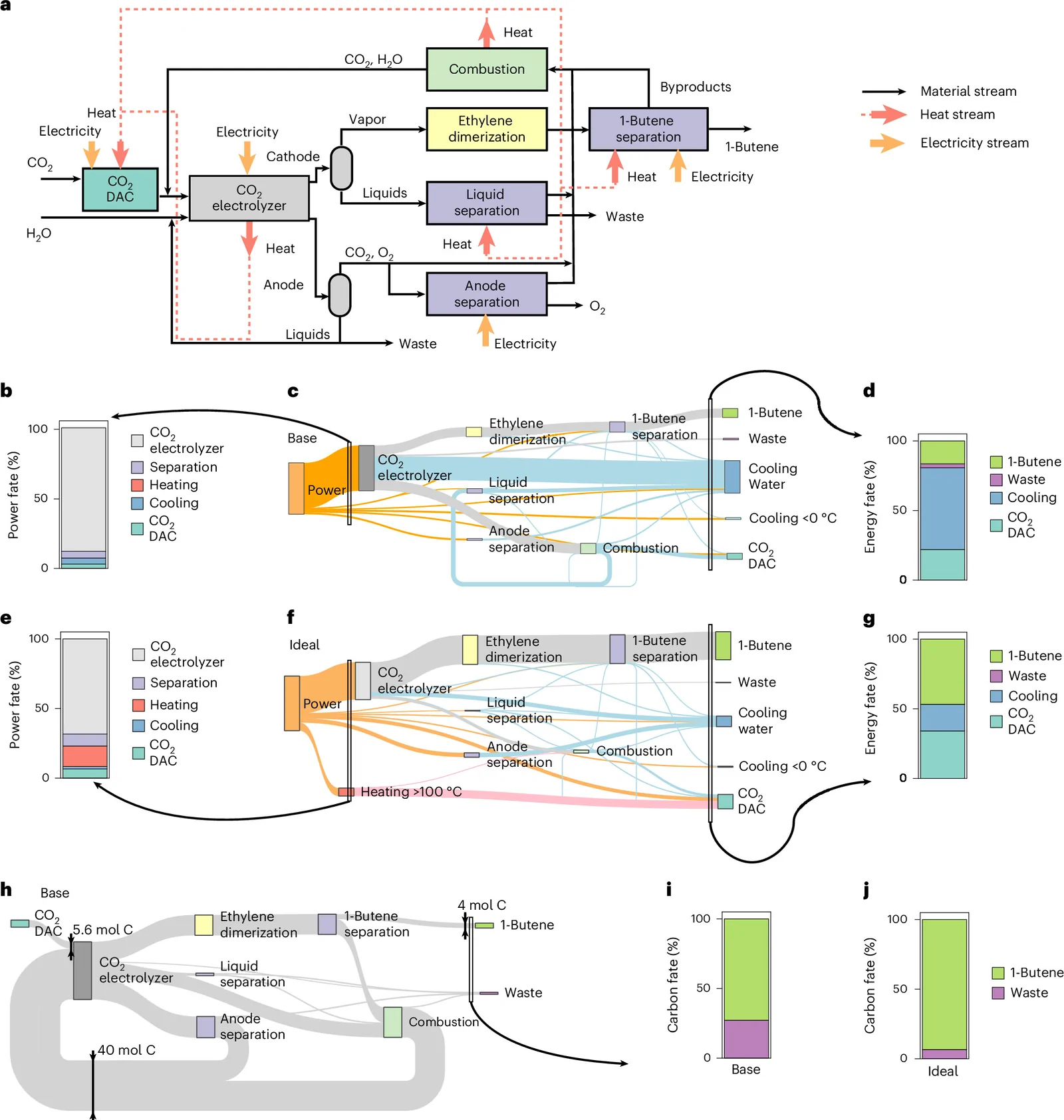
Flow diagrams for the electrochemical reduction of CO_2_ to ethylene and dimerization to 1-butene. **a**, Process flow diagram for the electrochemical CO_2_ reduction to ethylene and dimerization to 1-butene, including separation units. CO_2_ is supplied via DAC, and byproducts are combusted to CO_2_ and H_2_O, which are recycled to the electrolyzer. Heat integration is used to recover waste heat. **b**–**g**, Sankey diagram of the energy flows in the process (**c** and **f**), relative share of power input to the process (**b** and **e**) and energy end flow leaving the process (**d** and **g**) for the base case (for **b**, **c** and **d**) and ideal case (for **e**, **f** and **g**). The energy flows comprise electrical energy from the power input, chemical energy stored in products and waste streams, and thermal energy (heating and cooling, internal heat recovery) in the case of ambient-temperature operation of the electrolyzer. **h**, Sankey diagram of the carbon flows in the process for a base case considering current experimental developments (for example 50% ethylene Faradaic efficiency and 2.5 V in the electrolyzer. **i**,**j**, Relative share of carbon in 1-butene product stream and waste streams for the base case (**i**) and ideal case (**j**) (100% ethylene Faradaic efficiency and 1.4 V in the electrolyzer). Source data

In the tandem process studied herein, direct air capture (DAC) supplies CO_2_, which is converted to ethylene in the electrolyzer unit (Fig. 1a). Afterward, the produced ethylene is directly dimerized to the target product 1-butene without intermediate purification. The two tandem reactors, CO_2_ electrolyzer and ethylene dimerization, are followed by separation units for product purification. By directly targeting 1-butene, a challenging C1/C2 separation is circumvented by a final C1/C4 separation, which allows for the recovery of the reaction byproducts hydrogen, carbon monoxide, ethanol, acetate, n-propanol and butane. Furthermore, distillation-based C4 separation is established in industry^47,48^, whereas most other electrolyzer downstream separation technologies are still in development^27^.

Analyzing the fully integrated process shows two opportunities for internal heat recovery around the CO_2_ electrolyzer: (1) the combustion of byproducts for thermal energy generation and (2) the utilization of electrolyzer thermal losses. Although combustion enables thermal energy recovery at an elevated temperature independent of the electrolyzer operation, the waste heat utilization is bound to the electrolyzer operating temperature. In our process, the byproducts in the reaction units are combusted to CO_2_ and H_2_O, which are electrolyzer feedstocks. Combusting small byproduct streams offers the combined benefits of recycling feedstock, reducing gas separation needs and providing heat for processes such as CO_2_ capture at sufficiently high temperatures.

If the internal heat integration is not sufficient to supply all thermal energy, we consider two scenarios for external heating utilities: In the first scenario, thermal energy is supplied via heat pumps for low-temperature heat, resistance heating for higher temperatures and electrified cooling. However, current chemical parks usually provide utilities such as heat by natural gas, which is therefore typically considered in techno-economic analyses of electrochemical systems^21,24,49^ and considered in our analysis for comparison as a second scenario. To explore the full range of electrolyzer performance, we define a base case based on an electrolyzer performance that can be achieved experimentally today^23,32^. To provide an upper bound for the system performance, an ideal case is considered a theoretical benchmark. This ideal case reflects a 100% ethylene Faradaic efficiency and a 1.4 V cell voltage assumption. While we acknowledge that this benchmark may be out of practical reach for a complex 12-electron product, such an ideal case often serves as an important reference point in techno-economic or system-level literature^3,21,32,50^: if the ideal case does not outperform the benchmarks, there is no need for its further development. Its benefits indicate the maximum potential gains and thus a budget to be invested.

The main process energy demands are for the electrolyzer, the separation units and DAC (Fig. 1b). The electrolyzer consumes most of the electricity provided to the process (Fig. 1c). However, only 22% of the electrical input is chemically stored in 1-butene (Fig. 1d). In the base-case scenario, 50% of the energy dissipates as waste heat, resulting in a substantial cooling demand for the electrolyzer^51,52^. The additional electrical energy supplied to the electrolyzer is attributed to the overpotential caused by losses and inefficiencies, increasing electricity demands and cooling requirements. Reducing the overpotential from losses and inefficiencies is crucial to minimizing energy losses in low-temperature electrolysis. The ideal case improves the energy efficiency to 1-butene to 62% (Fig. 1g). The dimerization reaction is exothermic, thus requiring no energy input. After the electrolyzer, the main energy consumer is CO_2_ capture, driven by both electricity and heat.

Thanks to heat integration, the base case requires no external heat, as the combustion of byproducts generates sufficient thermal energy to operate other process units, including CO_2_ capture (Fig. 1c). However, energy losses occur via the energy demands of separation and via byproduct loss to waste. The ideal case improves the electrolyzer performance, but the heat generated from combustion is insufficient to drive the DAC due to the reduced amount of byproducts at high ethylene Faradaic efficiency. Consequently, additional heat is required. Thus, the ideal case requires external heat, while the base case can rely on internally recovered heat (Fig. 1e). However, the reduction in the electrolyzer energy demand reduces the overall electricity demand for the process and utilities from $258\,{\rm{GJ}}\,{{\rm{t}}}_{1\mathrm{-}{\rm{butene}}}^{-1}$ to $93\,{\rm{GJ}}\,{{\rm{t}}}_{1\mathrm{-}{\rm{butene}}}^{-1}$.

Due to incomplete CO_2_ conversion in the electrolyzer, recycle streams are substantially larger than the desired product flow, resulting in large internal carbon flows relative to the product for the base case (Fig. 1h). Specifically, the recycled carbon stream to the electrolyzer is seven times greater than the fresh CO_2_ feed stream. This disparity is due to incomplete conversion in the electrolyzer, the formation of byproducts and carbon crossover to the anode. The cathode byproduct mixture of CO_2_, carbon monoxide, hydrogen and other trace gases is combusted with the anode CO_2_ and O_2_ mixture. The resulting pure CO_2_ and H_2_O stream is then recycled as the electrolyzer feed. Despite the large streams, the low separation energy ensures these internal flows do not dominate the overall energy demand^26^.

However, approximately 30% of carbon is lost to waste, mainly as byproducts or within recycle streams, assuming a 1% loss in every separation unit^24,28,53^. The loss is reduced to 6% in the ideal case, assuming a high ethylene Faradaic efficiency (Fig. 1i,j). Although the ideal case demonstrates lower carbon loss, it still involves large recycle streams, amounting to more than four times the feed stream, primarily due to anode crossover. These large recycle streams increase efforts for fluid handling and equipment in industrial plants^26^. The carbon loss also increases energy demands for CO_2_ capture. Additional results and plots for the ideal case are provided in the Supplementary Information.

The liquid electrolyzer outlet stream contains water losses from CO_2_ humidification and byproducts such as ethanol, acetate and n-propanol. These liquid components are separated from the gas product stream, which mainly contains CO_2_, ethylene, carbon monoxide and hydrogen. Typically, the Faradaic efficiencies to liquid byproducts are low compared to the Faradaic efficiencies to ethylene, carbon monoxide and hydrogen^54^. However, even small Faradaic efficiencies to liquid byproducts can result in high carbon losses, reducing performance. In the literature, liquid byproducts are frequently neglected and deemed unimportant, especially when CO_2_ supply costs are low (for example, $\mathrm{US}{\rm{\$}}0\,{{\rm{t}}}_{{\mathrm{CO}}_{2}}^{-1}$)^24,38^. However, the mixed stream of liquid byproducts can be costly to separate and utilize. The separation challenge is compounded by the fact that some of the components, such as n-propanol, form azeotropes with water.

### Thermal energy demands and the potential for heat integration

**Fig. 2:**
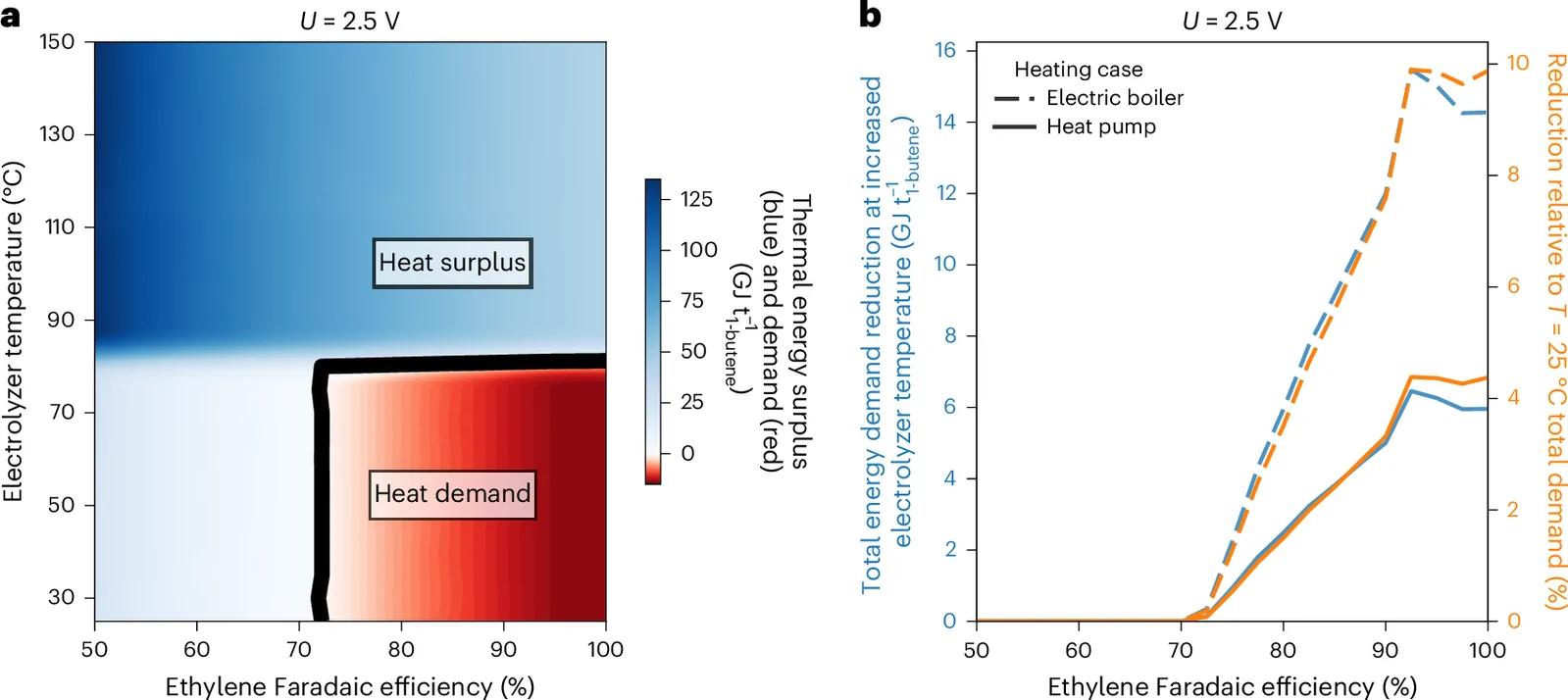
Thermal energy supply and demand—electrolyzer temperature, cell voltage and Faradaic efficiency. **a**, Overall process thermal energy supply and demand for varying Faradaic efficiency and electrolyzer temperature at a constant cell voltage of 2.5 V. **b**, Total energy demand reduction for process and utilities via heat integration enabled by a higher electrolyzer temperature of varying Faradaic efficiency at a constant cell voltage of 2.5 V. The energy demand reduction is given for the two heating cases of an electric boiler and a heat pump. The nonsmoothness occurs because the heat integration is energetically, not economically, optimized. Source data

While electricity demands have been the focus of studies on electrochemical processes, the absolute thermal energy demand of downstream units can exceed the electrical energy demand of the electrolyzer^23^. In our process, thermal energy is mainly consumed by the CO_2_ capture unit, with minor demands from the other separation units (for example, distillation columns). If the electrolyzer is operated at ambient temperature (Fig. 2a), external heat is required above ethylene Faradaic efficiencies of about 70%. Increasing electrolyzer performance toward more ethylene strongly reduces the electricity demand (see above), but reduces the heat integration potential. Without any heat integration or byproduct energy recovery, the thermal energy demand would range from $19\,{\rm{GJ}}\,{{\rm{t}}}_{1\mathrm{-}{\rm{butene}}}^{-1}$ to $30\,{\rm{GJ}}\,{{\rm{t}}}_{1\mathrm{-}{\rm{butene}}}^{-1}$. Crucially, maximizing target product efficiency remains the primary thermodynamic priority, as avoiding byproduct formation saves substantially more electrical energy than byproduct combustion can recover as heat. Byproduct combustion is therefore not the primary design objective but a waste-heat recovery measure to be considered.

Higher electrolyzer temperatures enable the utilization of electrolyzer waste heat for CO_2_ capture (Fig. 2a), thereby reducing the total energy demand of the 1-butene process by up to 10% in the direct electric heating case (Fig. 2b). Such a decrease in the total electricity demand for electrolyzer, down- and upstream units, as well as heating utilities, would correspond to a Faradaic efficiency increase of 10%. When the electrolyzer operates at temperatures above 90 °C, heat utility demands are fully eliminated (Fig. 2a). At about 90 °C, the electrolyzer waste heat can be directly utilized by the DAC system^55^. Only at ethylene Faradaic efficiencies above 95% and cell voltage below 1.5 V, heating utility is still required for electrolyzer temperatures above 90 °C (Supplementary Information).

In contrast to these findings, temperature is often overlooked in CO_2_ electrolyzer development, as indicated by current laboratory practices predominantly conducted at ambient temperatures^5,7,8,56^. Reasons for ambient-temperature operation are reduced experimental complexity and a lower entry barrier compared to temperature-controlled systems^51,52^. In the ambient-temperature case, the overpotential in the cell primarily dissipates as waste heat, which can not be integrated and even necessitates substantial cooling in large-scale electrolyzers^36^. However, commercial PEM water electrolysis routinely operates at 80 °C, establishing a precedent for higher-temperature feasibility^57^. To bridge this gap for CO_2_ electrolyzers, future research must evaluate membrane degradation, crossover and catalyst stability at elevated temperatures^58,59^. Transitioning to 80–90 °C operation is a key recommendation of this work to exploit favorable local reaction environment effects^60^ and to establish cell reliability, as supported by the environmental and economic margins quantified through process integration.

### Link between electrolyzer KPIs and climate change impacts

The climate change impacts (expressed in CO_2_ equivalents, CO_2_eq.) of the 1-butene process depend on the electrolyzer characteristics, such as its temperature and Faradaic efficiencies, and the integration into the electrochemical tandem process leads to counter-intuitive behavior. In general, we would expect that low temperatures and high ethylene Faradaic efficiency decrease climate change impacts. However, this behavior is not always observed, demonstrating the strong impact of process design on electrolyzer design targets.

**Fig. 3:**
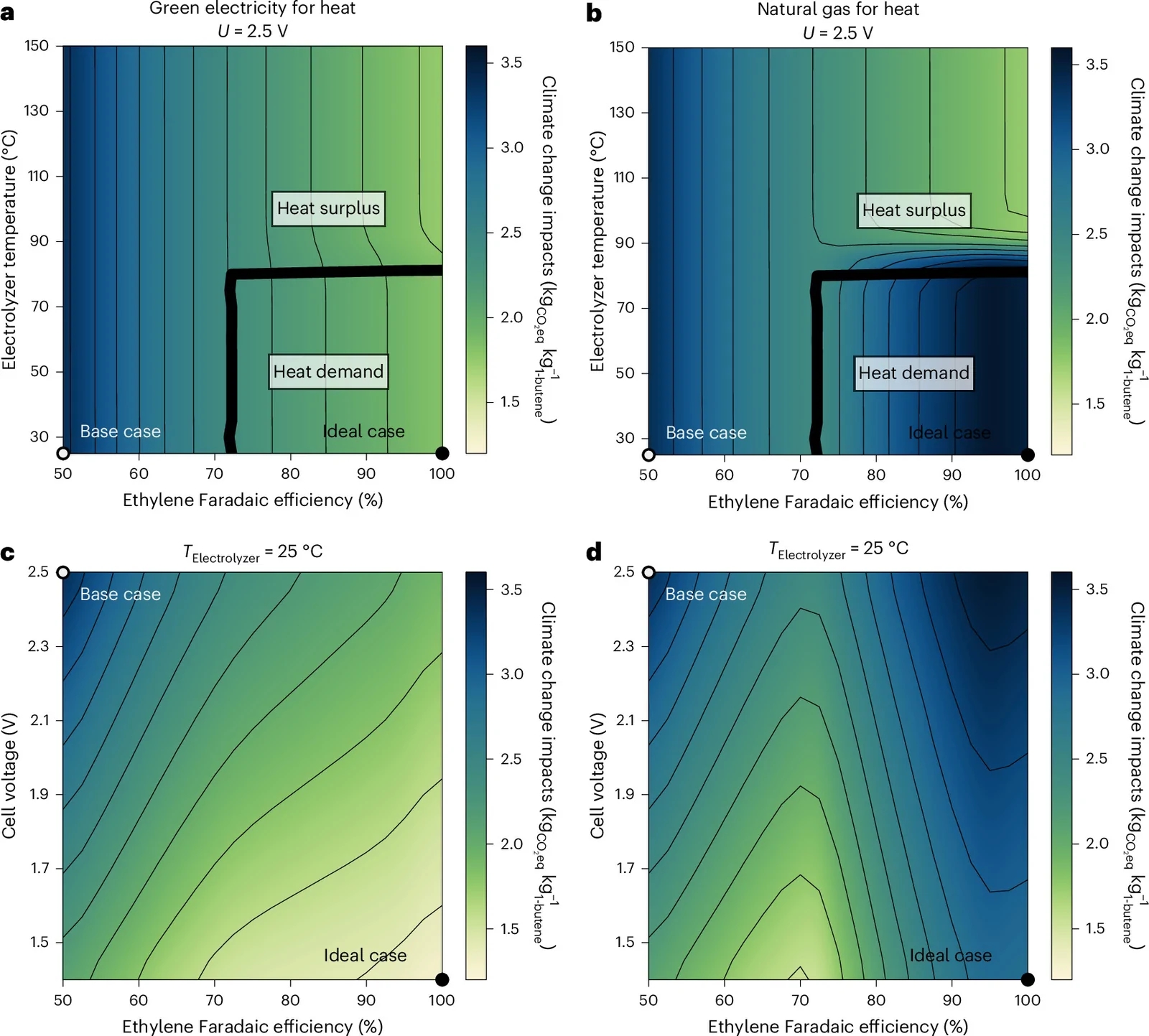
Process performance in climate change impacts for varying electrolyzer KPIs. **a**,**b**, Climate change impacts for varying electrolyzer temperature and ethylene Faradaic efficiency (FE) for heating via green electricity (**a**) and natural gas heating (**b**) (cell voltage of 2.5 V). The base case and the ideal case from Fig. 1 are marked as points. **c**,**d**, Climate change impacts for varying cell voltage and ethylene FE for heating via green electricity (**c**) and natural gas heating (**d**) (electrolyzer temperature of 25 °C). The base case and the ideal case from Fig. 1 are marked as points.

Higher electrolyzer temperatures reduce the climate change impacts of the electrochemical 1-butene production (Figs. 3a,b), and yet much electrolyzer research today focuses on near-ambient-temperature operation^11,61^. Higher electrolyzer temperatures enable the utilization of electrolyzer waste heat for CO_2_ capture. The effect on climate change impacts is particularly pronounced for the current natural gas-based heat supply (Fig. 3b). Prior techno-economic analyses and life cycle assessments of electrochemical processes have focused on decarbonized electricity for the CO_2_ electroreduction processes but still relied on inexpensive thermal energy^4,15,21,24,28,31,36,62–64^. These utility systems reflect the current transition efforts of the chemical industry with increasing procurement of renewable electricity, while heat and steam generation still rely on on-site natural gas combustion^65–69^. Increasing Faradaic efficiencies indeed improves climate change impacts of the 1-butene process if utilities are supplied from decarbonized electricity (Fig. 3a). However, with current utility systems, increasing ethylene Faradaic efficiency can increase climate change impacts (Fig. 3b). Using a fossil-based heat source exceeds the gain of electricity demand reduction by higher Faradaic efficiency, such that the climate change impacts increase with higher ethylene Faradaic efficiency (Fig. 3b). Our study underscores the importance of decarbonizing not only the electricity supply but also the heating system.

Decreasing cell voltage and increasing ethylene Faradaic efficiency reduces the electricity demand toward the minimal required energy to drive the electrochemical conversion of CO_2_ to ethylene (${E}_{\min }=47.5\,{\rm{GJ}}\,{{\rm{t}}}_{1\mathrm{-}{\rm{butene}}}^{-1}$)^23^. Decreasing cell voltage and increasing ethylene Faradaic efficiency are, thus, clear targets for future research. The process assessment now allows us to quantify these benefits and their trade-offs: increasing Faradaic efficiency by 10 percentage points starting at the base case equates to reducing cell voltage by approximately 0.3 V for the scenario of a decarbonized heating system (Fig. 3c). A similar trend holds for the nondecarbonized heat supply scenario if the Faradaic efficiency is below 70% (Fig. 3d). Above 70%, the high heat-related climate change impacts dominate the gain by cell voltage reduction, indicating again the importance of the heating system.

### Climate change and cost benefits of heat integration

**Fig. 4:**
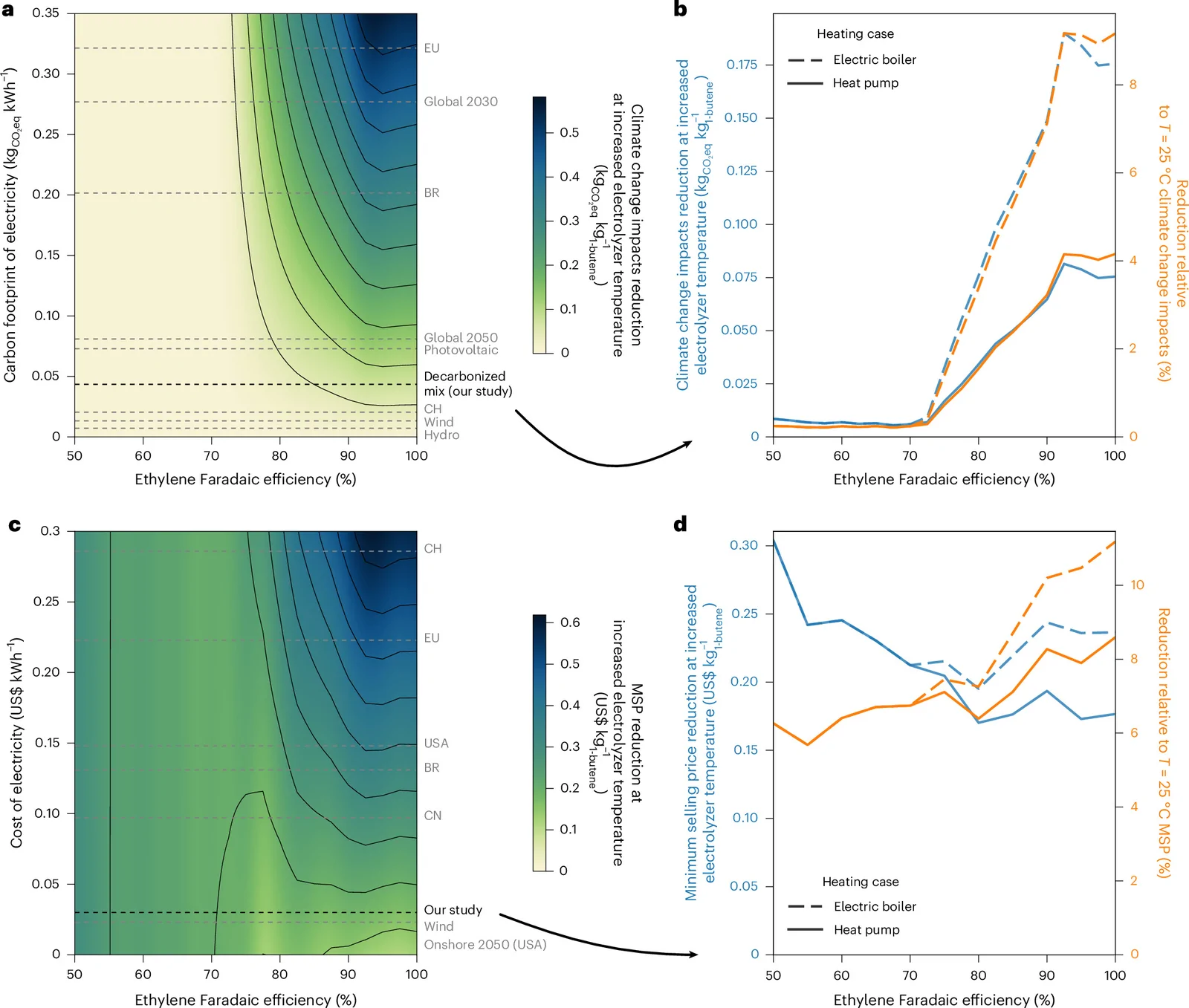
Climate change impacts and cost reduction potential via elevated electrolyzer temperature. **a**, Climate change reduction potential via electrolyzer waste heat utilization over Faradaic efficiency and electricity carbon footprint. Without waste heat utilization, a heat pump is used (constant cell voltage of 2.5 V). For reference, the electricity supply from renewables (photovoltaic, wind and hydropower), countries (European Union (EU), Brazil (BR) and Switzerland (CH)) and two global forecasts for 2030 and 2050 are given. **b**, Climate change impacts reduction via heat integration enabled by a higher electrolyzer temperature of varying Faradaic efficiency at a constant cell voltage of 2.5 V. The reduction is given for the two heating cases of an electric boiler and a heat pump. **c**, MSP reduction potential via electrolyzer waste heat utilization over Faradaic efficiency and electricity cost. Without waste heat utilization, a heat pump is used (constant cell voltage of 2.5 V). For reference, the electricity supply from renewables (wind), countries (Switzerland (CH), European Union (EU), United States of America (USA), Brazil (BR) and China (CN)) and a forecast (wind onshore 2050 (USA)) are given. **d**, MSP reduction via heat integration enabled by a higher electrolyzer temperature of varying Faradaic efficiency at a constant cell voltage of 2.5 V. The nonsmoothness occurs because the heat integration is energetically, not economically, optimized.

The reduced energy demand from elevated electrolyzer temperature (Fig. 2) also translates into reduced climate change impacts and costs (Fig. 4). We find that the heat-integrated tandem system can reduce climate change impacts from today’s fossil production by more than 30%; the ideal system would reduce climate change impacts even by 75% (base case and ideal case with electrified heating). Thus, even current state-of-the-art technology could already substantially reduce climate change impacts compared to the fossil benchmark. The estimated minimum selling price (MSP) to produce 1-butene ranges from $5.6\,{\mathrm{US}} {\rm{\$}}_{2023}\,{{\mathrm{kg}}_{1{\mathrm{-}}{\mathrm{butune}}}^{-1}}$ to $1.3\,\mathrm{US}{{\rm{\$}}}_{2023}\,{\mathrm{kg}}_{1\mathrm{-}\mathrm{butune}}^{-1}$ (base case and ideal case, electric heating). Thus, the ideal case is in the range of the fossil reference price of $1.09\,\mathrm{US}{{\rm{\$}}}_{2023}\,{\mathrm{kg}}_{1\mathrm{-}\mathrm{butune}}^{-1}$ to $1.87\,\mathrm{US}{{\rm{\$}}}_{2023}\,{\mathrm{kg}}_{1\mathrm{-}\mathrm{butune}}^{-1}$ (ref. ^70^). Noteworthy, the capital expenditures (CAPEX) for CO_2_ electrolyzers are highly uncertain because the technology remains at a low technology readiness level and economic data in the literature vary widely^40^ (Supplementary Information).

Elevating the electrolyzer temperature could reduce climate change impacts and cost by up to 9% and 11%, respectively (Fig. 4b,d). The benefits in climate change impacts result from and thus follow the behavior of energy savings qualitatively, with savings occurring above Faradaic efficiencies of 70% (compare Fig. 4b versus Fig. 2b).

By contrast, elevating the temperature reduces costs for all Faradaic efficiencies (Fig. 4d). In addition to the OPEX benefits from the reduced thermal energy demand, higher electrolyzer temperatures also reduce CAPEX. These savings result from increased temperature differences across heat exchangers, reducing their required area and installation costs.

The system-wide savings must offset the trade-offs of high-temperature operation, which has been observed to accelerate membrane degradation^58,71,72^. However, process integration benefits mostly outweigh the economic and environmental impacts of more frequent stack maintenance (Supplementary Information). From a climate change perspective, the frequency of stack component replacement could increase by a factor of 4 while remaining environmentally beneficial. From a cost perspective, doubling the frequency of stack maintenance still yields net economic savings. Overcoming these degradation mechanisms is therefore a vital research priority, where improving high-temperature stack reliability directly amplifies the economic and environmental margins quantified in this work.

### Implications for other CO_2_ electroreduction processes

**Fig. 5:**
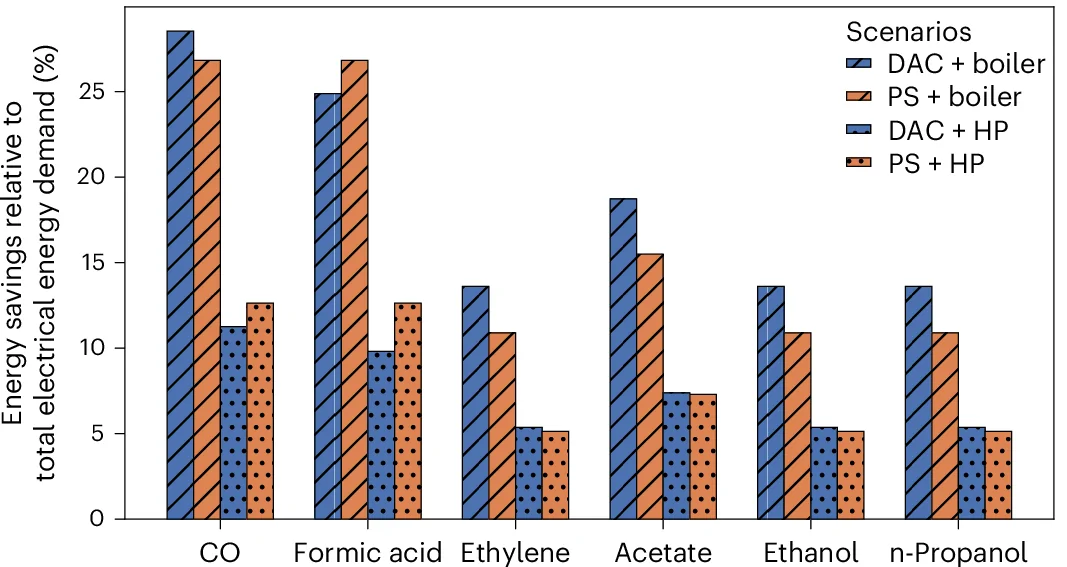
Maximum electrical energy savings potential via electrolyzer waste heat utilization. Relative electrical energy savings potential (electricity and heat supply) for the CO_2_ electroreduction products CO, formic acid, ethylene, acetate, ethanol and n-propanol for a cell voltage of 2.5 V and Faradaic efficiency of 100%. The utilization of electrolyzer waste heat and the savings in heating utility are compared to the total electricity demand. For the CO_2_ capture, a DAC system^55^ and a solvent-based point-source capture (PS)^117,120,121^ are considered. For the heat supply, an electric boiler (Boiler) and a heat pump (HP) are compared. Source data

The prior results are specific to the 1-butene case study. However, the energetic benefit of elevated CO_2_ electrolyzer temperatures generalizes to other CO_2_ electroreduction processes. Comparing the electrolyzer waste heat with the stoichiometric thermal energy demand for the CO_2_ supply indicates the potential for saving between 5% and 27% of the total electricity demand to drive the electrified process utilities (Fig. 5). The savings potential is even higher for small molecules such as CO and formic acid because the relative energy demand for CO_2_ capture is high, as fewer electrons are transferred during electrochemical conversion. The results match those from previous sections, accounting for differences in model detail and additional energy demands for separation, CO_2_ losses and inefficiencies. Thus, increasing the electrolyzer temperature generally unlocks heat integration of electrolyzer waste heat to drive thermally driven CO_2_ capture processes and potentially other separations. The findings hold for both DAC and point sources of CO_2_. The difference between DAC and point sources on the energy system is much smaller than the switch from an electric boiler to heat pumps, emphasizing again the importance of the heating system for integrated CO_2_ electrochemical conversion processes. Furthermore, because the available waste heat exceeds the thermal demand for CO_2_ capture, the surplus energy can be exported to adjacent processes. Consequently, elevated-temperature CO_2_ electrolyzers evolve into active thermal utilities to be integrated within site-wide industrial networks.

## Discussion

Process integration determines the trade-offs and targets for CO_2_ electroreduction development. In this context, the electrolyzer temperature is an often-overlooked key design variable, which can enable heat integration. By achieving higher operating temperatures, energy losses can be utilized in other process units. However, pushing electrolyzers toward waste heat utilization requires overcoming challenges associated with higher temperatures, such as additional equipment for temperature management, thermal stability (for example, membranes), and adjusted CO_2_ humidification. These considerations are critical for industrially relevant systems.

Operation at higher temperatures may impose additional safety and operability requirements. Especially, the assumed byproduct combustion can be challenging^73^. However, the process can benefit from existing oxy-fuel processes^74^. To prevent too high flame temperatures, large streams of inert CO_2_ support the safe operability of the combustion^75,76^. Furthermore, catalytic combustion could support operability^77^.

During operation, substantial cooling is required for both CO_2_ electrolyzers as well as water electrolyzers for hydrogen production^36^. Heating the feed and maintaining stable operating conditions require heat management capabilities^78^. In our process, the feed is heated by cooling the electrolyzer outlet streams and integrating both streams via heat exchangers. Furthermore, a temperature gradient is expected across the membrane and along the length of the electrolyzer plate. Hence, actively heating the electrolyzer to operating temperature can be omitted due to the ohmic waste heat generated in the electrolyzer. For active cooling, the liquid anode medium has been considered^51^. Furthermore, ref. ^36^ investigated cooling plates internally between the electrolyzer cells^36,79^. Both options could also be used for heat integration and would demand only little energy for pumping and control, which should still be quantified in detailed design studies. Practical challenges arise from the more complex system integration of, for example, CO_2_ capture, electrolyzer and heat. A heating utility system would have to balance fluctuations in the heat supply and in the concentrations of feedstock and product. Here, buffer systems and alternative heat supply might be required.

The implementation of elevated temperature electrolyzers and waste heat utilization is more mature for water electrolysis^80–82^. Arthur et al.^83^ demonstrated a thermally integrated system for water electrolysis. Due to lower overvoltage, the waste-heat potential is lower for water electrolysis than for CO_2_ electrolysis. For CO_2_ electrolysis, the potential to recover waste heat using a heat pump has been discussed qualitatively^52^. Recent efforts have experimentally demonstrated CO_2_ electrolysis at elevated temperatures^84^ and through detailed device-scale simulations^51^, confirming the potential for realizing waste heat utilization.

Current CO_2_ electroreduction processes are more expensive than fossil-based chemical production, but they can reduce climate change impacts when powered by green electricity (‘Link between electrolyzer KPIs and climate change impacts’). However, the infrastructure and electrolyzers still present challenges: High costs may drive the development of new catalyst materials^85,86^. But reductions in catalyst loading of metals and economies of scale might reduce costs^38,87^. In addition, the low technology-readiness level of large-scale CO_2_ electrolyzers might indicate further potential for improvement.

CO_2_ electrolyzer development involves trade-offs in multiple KPIs. Based on our analysis, we identify four primary future directions for the research community:

1. Experimental investigation of increased temperature for CO_2_electrolyzers: an understanding of how increased temperatures affect the efficiency and performance of CO_2_ electrolyzers influences process design. This task involves experimental studies to optimize operating conditions and improve overall process integration.
2. Upscaling CO_2_electrolyzers for more field data on both cost and environmental impacts of equipment: reliable quantification is needed of both the cost and environmental impacts associated with the installation and operation of large-scale CO_2_ electrolyzer equipment to guide further development.
3. Integrated energy and synthesis systems around CO_2_ electrolyzers: investigating the integration of cooling systems and waste heat utilization with CO_2_ electrolyzers is essential. This includes exploring the decarbonization of utility systems, connecting electroreduction processes with CO_2_ capture and utilizing (internal) heat pumps to enhance energy efficiency. Integrated systems pose challenges in terms of start-up, dynamics and flexibility^78^, which should be investigated. Furthermore, the synthesis of chemicals using CO_2_ electroreduction should be seen in context with other low-carbon alternatives to identify the most promising synthesis pathway.
4. Separation of mixtures in electrochemical plants: historically, the separation of mixtures in CO_2_ electroreduction systems has not been a major focus, but separation will become increasingly important. Separation design involves developing methods to efficiently separate mixtures, such as anode CO_2_ and O_2_, or ethylene, from high-impurity streams containing CO_2_, carbon monoxide, hydrogen and other substances. Moreover, the loss or utilization of liquid product streams in CO_2_ electrolyzers should be addressed. Depending on the Faradaic efficiency, substantial carbon and energy losses can be observed in liquid streams.

This research agenda should help advancing CO_2_ electrolyzers toward implementation.

## Methods

The process for CO_2_ electroreduction to ethylene and further dimerization to 1-butene is described in the following. The individual modeling steps are outlined here and in the Supplementary Information. The process concept is built upon early-stage experimental work by refs. ^13,14^ and process development by ref. ^23^ and ref. ^26^. Although the synthesis of 1-butene via the described route has been experimentally proven^13,14^, while the process design and upscaling are still at a low technology readiness level. Therefore, this study and the methods are of an explorative nature, assuming a higher technology readiness level of the process. The methodological details and assumptions are provided below.

### Process modeling of two-step 1-butene synthesis from CO_2_

The investigated process produces 1-butene from CO_2_ and H_2_O in a two-step process^13,14^: first, an electrolyzer produces ethylene from CO_2_ and H_2_O. Afterward, ethylene is dimerized to produce 1-butene (Fig. 1a).

The main unit of the ethylene section is the electrolyzer. Humidified CO_2_ is electrochemically reduced to ethylene in a membrane electrode assembly electrolyzer. On the cathode side, CO_2_ and H_2_O enter and react to ethylene and byproducts (hydrogen, carbon monoxide, ethanol, acetate and n-propanol)^23^. On the anode side, O_2_ is formed due to mass transfer over the membrane to the anode side. Furthermore, CO_2_ transfers through the membrane for charge exchange^88^. The crossover of liquid products from the cathode to the anode side is not considered here, acknowledging the unsolved but addressable problem^89,90^. The influence of temperature on product crossover and selectivity is governed by competing effects^58,60,90^: higher temperatures can promote vaporization of volatile products, decreasing their solubility in the electrolyte, but they also increase diffusion through the membrane. The net outcome depends on the specific system configuration, catalyst and operating conditions and is, therefore, not modeled in detail here. We consider a replacement of the liquid anolyte, which might be contaminated with traces of liquid products. At both the anode and cathode, the outlet streams are separated into a liquid and a gas stream in flash drums.

The second step is the ethylene dimerization to 1-butene^13,14^. In the dimerization reactor, ethylene is converted to 1-butene, and n-butane is formed as a byproduct. The dimerization outlet consists of unreacted CO_2_, 1-butene and byproducts.

Beyond the two reaction steps, the process requires separation for both the 1-butene purification and the recycling of unreacted CO_2_. The separated byproducts are combusted to CO_2_ and H_2_O, and then recycled. The recycle stream eliminates the need for separating mixed streams, for example, a CO_2_/carbon monoxide split, to obtain pure CO_2_. In addition, the demand for the CO_2_ source is decreased. Furthermore, heat is generated by the combustion, which can provide heat for the CO_2_ capture and the separation units, such as distillation columns. If azeotropes occur in the distillation columns, only a defined purity below the azeotrope is reached. The remaining water/impurities in the recycle stream are accepted in the combustion process, and carbon losses to wastewater are accepted.

The whole process is modeled in Aspen HYSYS v11^91^ to calculate all mass and energy balances needed for process assessment. Details on the modeling and individual process sections are provided below and in the Supplementary Information.

The model for the CO_2_ electrolyzer is based on ref. ^23^ for a membrane electrode assembly electrolyzer. The model and its implementation in Aspen HYSYS meet the need for closed mass and energy balances and for independent variation of all electrolyzer KPIs, while remaining computationally simple and implementable in commercial flowsheet simulators focused on flowsheet-level flows. A cathodic CO_2_ conversion is defined to make the KPIs single-pass conversion to products, Faradaic efficiency and CO_2_ crossover independent variables. The cell voltage includes the required thermodynamic minimum voltage (approximately 1.15 V for ethylene^23^) and an overvoltage^8^.

The base-case ethylene Faradaic efficiency is based on current literature^23^. Higher ethylene Faradaic efficiencies and lower voltages have been demonstrated^32,92,93^. However, other parameters vary, such as the current density or voltage. Therefore, we assume a 50% Faradaic efficiency to ethylene coupled with a voltage of 2.5 V as a base case, which has been achieved repeatedly in literature^11,93–95^. For benchmarking reasons, all other parameters from the base case are based on ref. ^23^. For an ideal case, we assume an ideal 100% Faradaic efficiency to ethylene and reduce the cell voltage to 1.4 V, which is close to the theoretical minimum but incorporates overpotential to drive the reaction and serves as a theoretical upper bound. The ideal voltage aligns with development targets of below 2 V, as suggested for CO_2_ electroreduction^21,94,96,97^. The ideal case energy efficiency of 81% is in line with current targets of 80% (refs. ^98,99^) for water electrolyzers^100^. As the catalytic steps in CO_2_ electroreduction are more challenging than those in water electroreduction, we regard the ideal case as upper bound on the achievable performance, allowing us to establish if the process has potential benefits and their maximum size.

All Faradaic efficiencies to byproducts are decreased linearly when ethylene Faradaic efficiency increases to keep the overall Faradaic efficiency constant. All other KPIs are kept constant, as they are not the focus of the analysis and only affect the electrolyzer, without impacting system integration directly (for example, current density affects area and cost of the electrolyzer only). While current density and cell voltage are kinetically coupled (for example, via the Tafel slope^50^), assuming constant other KPIs enables a direct comparison across temperature and voltage scenarios without introducing additional variations in electrochemical performance. In practice, electrolyzer performance improvements can arise from changes in either cell voltage or current density-or from concurrent advances in both. The present model thus lumps the description of these interacting variables, which could be further decomposed in more detailed electrochemical models. We maintain the same current density and CO_2_ single-pass conversion, as increases beyond certain thresholds offer minimal system performance gains and diminishing economic returns due to higher energy costs outweighing investment cost reductions^26,61^. The base-case cathodic conversion and crossover correspond to approximately 25% overall single-pass CO_2_ conversion. Further details are provided in the Supplementary Information.

As previously described by ref. ^26^, the separation energy of electrolyzer product streams is minor compared to the electrolyzer energy demand. Furthermore, the CO_2_ partial pressure decreases with high CO_2_ conversion, and the ethylene Faradaic efficiency decreases with decreasing CO_2_ partial pressure^19,26^. Nevertheless, economic viability imposes a strict lower bound on single-pass conversion, typically around 10%. Beyond this economic minimum, overall system performance is governed entirely by the electrolyzer’s energy efficiency rather than further gains in single-pass conversion^15,32,61,101^. Because maximizing conversion yields no systemic energetic benefit and actively penalizes target selectivity^26^, it is not a rational objective for process optimization. To rigorously evaluate the primary energetic drivers, we therefore constrain the CO_2_ conversion to a constant baseline value throughout our analysis (Supplementary Information).

CO_2_ is supplied via DAC using temperature-vacuum swing adsorption, a process that requires both electricity and heat as inputs^55^. In addition, H_2_O is provided as utility, and the CO_2_ is humidified before entering the electrolyzer^7^. The operation of CO_2_ capture, electroreduction and separation also demands electricity, heating and cooling. Heating and cooling are provided via natural gas steam, electric boilers, heat pumps for low-temperature heat, cooling water and refrigeration, depending on the investigated case study. The temperature levels of the utilities are taken from default utilities provided in Aspen HYSYS v11^91^.

For the dimerization of ethylene to 1-butene, we assume a constant ethylene conversion of 93.3% to 1-butene (equation (1)) and 5.3% to n-butane (equation (2)) as byproduct based on experimental results^13,14^. As the focus of this study is the CO_2_ electrolyzer, the dimerization is not investigated in detail. We refer to established processes^102^ and experimental work^13,14,103,104^. However, we provide a sensitivity analysis on the ethylene conversion in the Supplementary Information.

1 $$1\mathrm{-}{\rm{Butene}}:2{{\rm{C}}}_{2}{{\rm{H}}}_{4}\longrightarrow {{\rm{C}}}_{4}{{\rm{H}}}_{8}$$

2 $${\rm{n}}\mathrm{-}{\rm{Butane}}:2{{\rm{C}}}_{2}{{\rm{H}}}_{4}+{{\rm{H}}}_{2}\longrightarrow {{\rm{C}}}_{4}{{\rm{H}}}_{10}.$$

The downstream separation system is modeled for each unit operation individually. Due to missing information and pre-commercial technologies, assumptions are made. Generally, a 99% and 1% split of components in separation units is used if not specified differently. An adsorptive dehumidification after the electrolyzer is used based on the experimental work by refs. ^13,30,105^. Liquid streams are separated using distillation. The 1-butene separation is similar to industrial separations with distillation units for the separation of lighter gases and heavier byproducts via two distillation columns^47,48^. For all other separations, we assume 7% entropic efficiency, as this seems to be a conservative approximation for the electrolyzer downstream separation^26^. The downstream separation is not the main energy consumer. Hence, we consider the assumption of 7% entropic efficiency for separation sufficient for the focus of our analysis and in line with the literature^26,27,32,106^.

All separated byproducts are combusted to CO_2_ and H_2_O and recycled, following ref. ^28^. O_2_ from the anode is used for the combustion. The anode stream allows for both controlling the combustion via dosing of O_2_ and for increasing safety via dilution with CO_2_. Alternative use of the O_2_ byproduct is not considered, and the remaining O_2_ is vented^30,107^ as the market demand is too low. Also, further purification would be needed^107^, which is outside the scope of this study.

### Heat integration

The energy demand of the process is reduced via heat integration. Full heat integration is achieved for all heating and cooling streams in the process via solving a mixed-integer optimization problem for a heat exchanger network design^108,109^. The heating system is either driven by green electricity for heat pumps for low-temperature heat and resistance heating for high-temperature heat or driven by natural gas to generate steam at higher temperatures. The cooling system relies on electricity to drive refrigeration cycles and cooling water flow. These electrification measures could also be applied to fossil-based production but would not lead to net-zero chemicals due to the fossil carbon source. Further details are given in the Supplementary Information.

### Techno-economic analysis

A techno-economic analysis is performed based on the flowsheet simulation and heat integration results. The used information includes material and energy flows and estimations of equipment size for calculating operating and investment costs for a chemical plant. The MSP for the production of a 1-butene in ${\mathrm{US}}{\rm{\$}}{{\mathrm{kg}}^{-1}_{{1}-{\mathrm{butene}}}}$ is the objective, and the method is based on ref. ^110^ (sometimes referred to as total annualized cost), where CAPEX represents the capital expenditures and OPEX the operational expenditures. CAPEX is estimated using cost correlations from literature^24,110^, and ACCR is the annual capital charge ratio. OPEX is estimated based on feedstock costs, utility costs and overhead costs. *m*_1-butene_ is the mass of 1-butene produced.

3 $${\mathrm{MSP}}=\frac{{\mathrm{ACCR}}\times{\mathrm{CAPEX}}+{\mathrm{OPEX}}}{{m}_{{1{\mbox{-}}{\rm{butene}}}}},$$

A plant size of 40 kilotons (kt) 1-butene annual production^111^ is assumed, which is in the range of smaller ethylene plants and large enough that economies of scale are expected to be utilized^24^.

The assumed optimistic electricity price is 0.03 US$_2023_ kW^−1^ h^−1^(refs. ^22,26,61,112^), which is below US$0.04 kW^−1^ h^−1^ suggested by ref. ^20^ for cost competitiveness of electrolyzers. While current electricity prices are often higher, we assume a future-reachable low price necessary^112^ to unlock the potential of CO_2_ electroreduction and to reach the range of cost competitiveness. The same applies to the CO_2_ electrolyzer investment cost assumptions, which are often extrapolated from water electrolyzers in the literature^4^. Electrolyzer stack lifetime and investment cost assumptions vary widely in the literature; we adopt optimistic values and highlight the existing gap in reported data (Supplementary Information). Due to lower technology readiness levels, learning rates are assumed for the investment cost of the CO_2_ capture equipment and the CO_2_ electrolyzer^8,113,114^. Further details on the techno-economic analysis assumptions are provided in the Supplementary Information.

### Life cycle assessment

A life cycle assessment is performed to estimate the environmental impact of the electrochemical 1-butene route^42,43^. The system boundaries include feedstock provision, the investigated process, utilities and 1-butene end-of-life combustion for a cradle-to-grave assessment, neglecting the use phase. The functional unit is the production and end-of-life combustion of 1 kg of 1-butene. For the background system, the ecoinvent database version 3.10 with the cutoff system model is used with data for the USA or global averages, depending on the availability^115^.

The CO_2_ feedstock is provided via DAC, with the life cycle inventories taken from ref. ^55^. The electricity is assumed to be provided via a decarbonized electricity mix (45% wind, 34% solar, 13% nuclear, 5% hydro and 3% other sources, according to the ReEDS Mid-Case 100% by 2035 scenario)^116^. The process utilities providing all power, heat and cooling are driven by the assumed electricity or natural gas, depending on the investigated case. Further data on equipment and waste treatment are assumed as global averages or US data if available (Supplementary Information).

### Generalization to other CO_2_ electroreduction processes

We assess the potential of thermal energy integration for other products aside 1-butene via a simplified energy analysis. The process is simplified to its key energy demands: energy for CO_2_ capture (thermal and electricity) and the electrolyzer. The electrolyzer converts the electrical energy input into chemical energy in product and byproducts, as well as ohmic waste heat.

To utilize the ohmic waste heat, the electrolyzer is assumed to operate at a temperature elevated sufficiently high to supply heat directly to the CO_2_ capture unit. We assume a sorbent-based DAC case (same as above)^55^ and a solvent-based point-source capture^117^.

The total electrolyzer energy demand is estimated via

4 $${w}_{\mathrm{electrolyzer},\mathrm{total}}=\frac{UF{z}_{\mathrm{product},i}}{{\mathrm{FE}}_{\mathrm{product},i}{M}_{\mathrm{product},i}},$$

where the variables describe the total cell potential *U*, Faraday constant *F*, the number of electrons transferred for the synthesis of the product from CO_2_ *z*_product,*i*_, the Faradaic efficiency FE_product,*i*_, the molar mass *M*_product,*i*_ and energy demand *w*. The electrochemical parameters are taken from literature^22,23,92,118^, and we assume *U* = 2.5 V and Faradaic efficiency of 100% for the analysis (Fig. 5). Because the minimum cell potentials ${U}_{\min ,i}$ are comparable across all target products, we assume a uniform total cell potential to isolate the primary system variables. The energy demand of the CO_2_ capture is scaled to the product mass (Supplementary Information).

## Supplementary information

Supplementary Information Supplementary Discussion and References.

## Source data

Source Data Fig. 1 Statistical source data.

Source Data Fig. 2 Statistical source data.

Source Data Fig. 5 Statistical source data.

## Data Availability

All data are publicly available. However, some cases require a user license from ecoinvent or IHS Markit to access the underlying data. All data that does not require an additional license is provided within the Article and its Supplementary Information, as well as in the Code availability.

## Code availability

All codes associated with this study are available via GitLab at https://gitlab.ethz.ch/epse/process-design-public/electrolyzer-process-integration. Furthermore, a copy is archived via Zenodo at https://doi.org/10.5281/zenodo.21477221 (ref. ^119^). However, commercial licenses for the flowsheet simulator Aspen HYSYS, the optimization solver Artelys Knitro and life cycle inventory data are required to execute the codes.
