# Supplementary material for: Elevating CO2 electrolyzer temperature accelerates the viability of chemical electrification
Source: Nat Chem Eng. 2026 Oct 19;3(10):597–610. doi: 10.1038/s44286-026-00444-6 (PMC13680281; doi:10.1038/s44286-026-00444-6)
Supplement: Supplementary file 1 [file 44286_2026_444_MOESM1_ESM.pdf]

nature chemical engineering

Supplementary information https://doi.org/10.1038/s44286-026-00444-6

Elevating CO_2 electrolyzer temperature accelerates the viability of chemical electrification

In the format provided by the authors and unedited

Contents

S1 Case study and process model **2**

S1.1 Case study: 1-Butene via CO_2 electroreduction and ethylene dimerization . 2

S1.2 CO_2 Electrolyzer . . . 4

S1.3 Ethylene dimerization to 1-butene . . . 8

S1.4 Separation . . . 8

S1.4.1 Anode separation . . . 11

S1.4.2 H_2O separation via adsorptive dehumidification . . . 11

S1.4.3 Cathode liquid separation . . . 11

S1.4.4 1-Butene separation . . . 11

S1.4.5 Combustion . . . 12

S1.5 O_2 byproduct . . . 13

S1.6 Heat integration . . . 13

S2 Details on the implementation and simulation **16**

S3 Methodological details on the techno-economic analysis **16**

S4 Methodological details on the life cycle assessment **22**

S5 Utility systems in literature **41**

S6 Results **44**

S6.1 Investment cost and climate change impacts of the electrolyzer construction in the literature. . . . 44

S6.2 Sankey diagrams for the ideal case . . . 48

S6.3 Thermal energy . . . 49

S6.4 Main contributors to climate change impacts and cost . . . 51

S6.5 CO_2 single pass conversion . . . 53

S6.6 Carbon efficiency . . . 55

S6.7 Byproducts and separation . . . 56

S6.8 Electrolyzer operation under pressure . . . 59

S6.9 Sensitivity analysis on ethylene dimerization . . . 60

S6.10 Flowsheet choices . . . 61

S6.11 Electrolyzer cell lifetime . . . 63

S6.12 Thermal energy saving potential estimation for other CO_2 electroreduction products . . . 66

S7 Discussion **79**

Bibliography **80**

S1. Case study and process model

The aim of this study is to provide a process perspective on electrolyzer development. Development targets for catalysts and electrolyzers are defined based on process models and performance assessments. The investigated case study is CO_2 electroreduction to ethylene (C_2H_4) and further dimerization to 1-butene.
The overall process for CO_2 electroreduction to ethylene and further dimerization to 1-butene is described in the following (see also section in main manuscript). A process performance assessment is performed via heat integration, techno-economic analysis, and life cycle assessment, and is described afterward. The impact of varying electrolyzer performance parameters on the overall process performance is evaluated by adapting the whole process to the electrolyzer performance. Additional results and discussions supplementing the main manuscript are provided.

The process is built upon experimental work by Lee et al. [1] and process development in the literature by Alerte et al. [2] and Moore et al. [3].

S1.1. Case study: 1-Butene via CO_2 electroreduction and ethylene dimerization

The investigated process produces 1-butene from CO_2 and H_2O in a two-step process [1]. First, an electrolyzer section produces ethylene from CO_2 and H_2O. Afterward, 1-butene is produced via ethylene dimerization. The overall process is shown in Figure S1 and is described below.

The main part of the ethylene section is the electrolyzer. Humidified CO_2 is electrochemically reduced to ethylene in a membrane electrode assembly (MEA) electrolyzer. The electrolyzer consists of a cathode side and an anode side separated by a membrane. An electrical current is applied to drive the desired chemical reactions. On the cathode side, CO_2 and H_2O enter and react to ethylene and byproducts (H_2, carbon monoxide, ethanol, acetate, n-propanol) [2]. On the anode side, a potassium anolyte solution is circulated, and O_2 is formed due to mass transfer over the membrane to the anode side. Furthermore, CO_2 transfers through the membrane for charge exchange. At both the anode and cathode, the outlet streams are separated into liquid and gas streams in flash drums.

The second step is the dimerization of ethylene to 1-butene [1]. The feed stream is the cathode gas outlet from the electrolyzer, dehumidified before entering the dimerization reactor. In the dimerization reactor, ethylene is converted to 1-butene, and n-butane is formed as

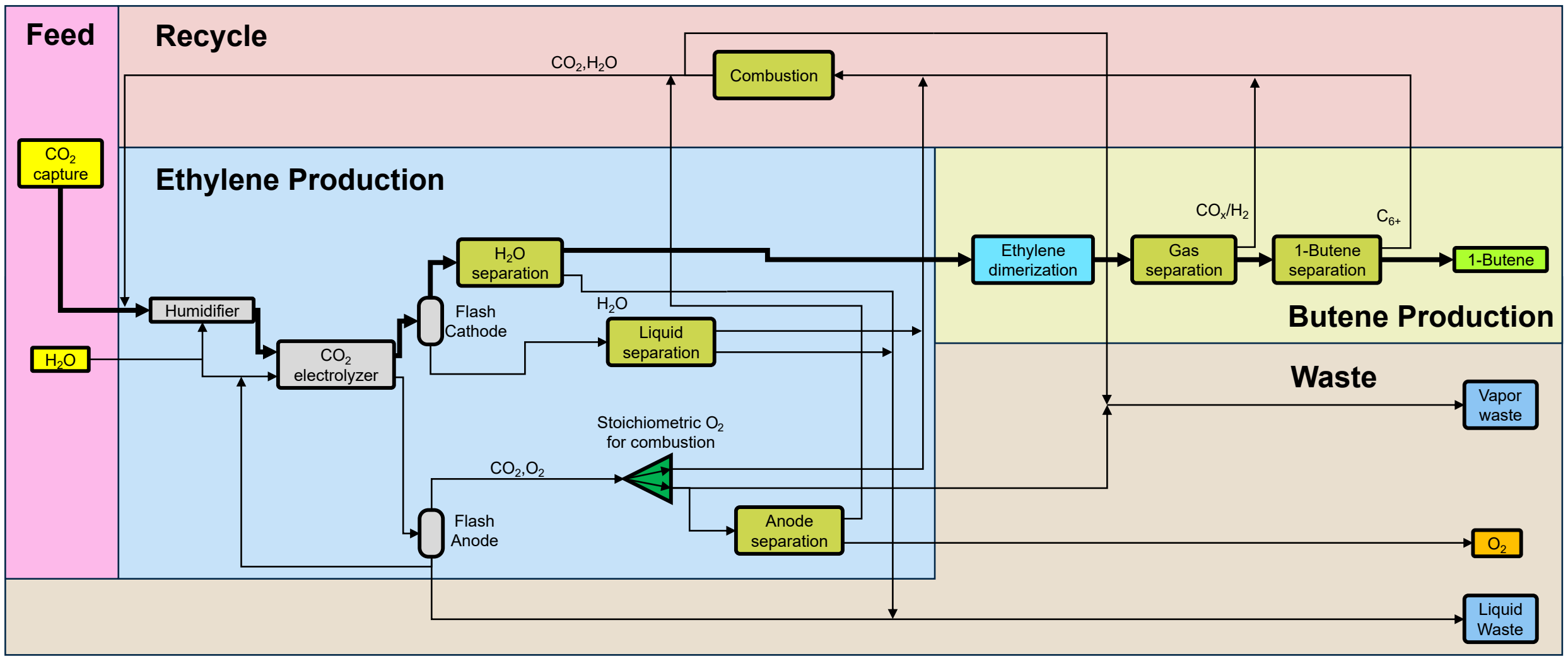

Figure S1: Process flow diagram for the CO_2 utilization via electroreduction and dimerization to 1-butene. The process comprises CO_2 capture (adsorbent-based direct air capture) and water feed, CO_2 electroreduction to ethylene (AEM electrolyzer) and downstream separation (entropic efficiency separation and distillation), ethylene dimerization to 1-butene with 1-butene separation (cryogenic distillation), and CO_2 recycle including combustion of byproducts.

a byproduct. The dimerization outlet consists of unreacted CO_2, 1-butene, and byproducts.

In addition to the two reaction steps, a separation step is required to obtain both the product and recycle the unreacted feedstock. 1-Butene needs to be purified to reach a product purity of 99.9 %. This purification includes first separating light gases and then heavier components.

In both reaction steps, byproducts are formed (H_2, carbon monoxide, ethanol, acetate, n-propanol, butane). The byproducts are separated from 1-butene, and the carbon is reused as feedstock for the electrolyzer. The separated byproducts are combusted to produce CO_2 and H_2O, which are then recycled. Via combustion, a separation of mixed streams, e.g., a CO_2/carbon monoxide split to obtain pure CO_2, is eliminated. Additionally, the provision of CO_2 from a CO_2 source is decreased. Furthermore, heat generated during combustion can be used to supply heat to the CO_2 capture unit and the separation units, such as distillation columns.

The electrolyzer anode side is relevant because its byproduct, O_2, is not involved in the reaction and separation chain for 1-butene production. On the electrolyzer anode side, O_2 and CO_2, which cross over the membrane (details in section S3), are separated to recycle the pure CO_2 to the electrolyzer. A part of the O_2/CO_2 mixture is fed to the combustion of

byproducts to provide the required O_2. The remaining O_2 is a byproduct.

In the recycle streams of feedstock to the electrolyzer, purge streams of 1 % are introduced to prevent byproduct accumulation and to account for losses. The 1 % is an assumption based on assumptions and suggestions in the literature [4–8]. Furthermore, liquid streams are wasted because they contain multiple components and high levels of water, which require high energy for separation.

In this work, we investigate the potential of CO_2 electroreduction and ethylene dimerization to 1-butene. Therefore, the described process is modeled, and its potential is assessed through techno-economic analysis and life cycle assessment. The purification of ethylene from byproducts after the electrolyzer is skipped, as experimental studies indicate the potential for direct dimerization of the ethylene stream with byproducts from CO_2 electroreduction [1].

S1.2. CO_2 Electrolyzer

The model for the CO_2 electrolyzer (CO_2 electrolyzer in Figure S1) is based on Alerte et al. [2] and applies to a membrane electrode assembly (MEA) zero-gap electrolyzer. For CO_2, the molar flows in the electrolyzer are split into an unconverted stream, a converted stream, and a crossover stream from the cathode side to the anode side (Figure S1.2).

The converted stream is calculated via the electrical current and the reaction equations (Eq. S5-Eq. S10).

$$\dot{n}_{\text{converted}} = \sum_{\text{Eq. S5−S10}} \frac{a_i \text{FE}_i}{b_i F} I \tag{S1}$$

The crossover stream is calculated via

$$\dot{n}_{\text{crossover}} = \frac{\tau}{F} I \tag{S2}$$

The outgoing stream on the cathode side is calculated via the cathodic CO_2 conversion

$$\dot{n}_{\text{unconverted}} = \dot{n}_{\text{converted}} \cdot \left(\frac{1}{X_{\text{CO2}}} - 1 \right) \tag{S3}$$

The entering cathode stream is the sum of all cathode outgoing streams

$$\dot{n}_{\text{c,in}} = \dot{n}_{\text{converted}} + \dot{n}_{\text{crossover}} + \dot{n}_{\text{unconverted}} \tag{S4}$$

a_i is stoichiometric number of CO_2 molecules per molecule of product i. FE_i is Faradaic efficiency to product i. b_i is the stoichiometric number of electrons transferred per molecule of product i. I is the total current. F is Faraday's constant. τ stoichiometric amount of CO_2 crossover per electron.

The separation of the crossover stream and the converted stream, together with the definition of cathodic CO_2 conversion and anodic crossover, allows for independent variation of conversion and crossover, which would otherwise be coupled. All reactions are given below. The variables of the model are provided in Table S1

Table S1: Parameters and variables in the electrolyzer model adapted from Alerte et al. [2].

Parameter/Variable	Unit
Input Parameters	
Cell voltage	V
Current density	$\frac{A}{m^2}$
$FE_{Ethanol}$	%
$FE_{Acetate}$	%
$FE_{n-Propanol}$	%
$FE_{Ethylene}$	%
$FE_{Carbonmonoxide}$	%
$FE_{Hydrogen}$	%
Cathodic CO_2 conversion X_{CO2}	−
Anodic CO_2 crossover	$\frac{mol_{CO2}}{mol_{e-}}$
Electrolyzer operating temperature	°C
Process variables	
CO_2 cathode feed stream	$\frac{mol}{s}$
Output variables	
Electrolyzer power	W
Electrolyzer current Electrolyzer area	m^2
CO_2 single pass conversion	−

Cathode reactions

$$\text{Hydrogen}: \quad 2H_2O + 2e^- \longrightarrow H_2 + 2OH^- \tag{S5}$$

$$\text{Carbon monoxide}: \quad CO_2 + H_2O + 2e^- \longrightarrow CO + 2OH^- \tag{S6}$$

$$\text{Ethylene}: \quad 2CO_2 + 8H_2O + 12e^- \longrightarrow C_2H_4 + 12OH^- \tag{S7}$$

$$\text{Ethanol}: \quad 2CO_2 + 9H_2O + 12e^- \longrightarrow CH_3CH_2OH + 12OH^- \tag{S8}$$

$$\text{Acetate}: \quad 2CO_2 + 6H_2O + 8e^- \longrightarrow CH_3COOH + 8OH^- \tag{S9}$$

$$\text{n-Propanol}: \quad 3CO_2 + 13H_2O + 18e^- \longrightarrow C_3H_8 + 18OH^- \tag{S10}$$

Anode reaction

$$4OH^- \longrightarrow 2H_2O + O_2 + 4e^- \tag{S11}$$

As indicated in Eq. S5 to S10, one OH^- is produced per electron e^-. The CO_2 in the alkaline cathode environment can react with the OH^- as given in Eq. S12 and S13. For electroneutrality and charge exchange, a negative charge transfer from the cathode to the anode is required to balance the electrical current from the anode to the cathode. The charge transfer can be OH^-, ${HCO_3}^-$, or ${CO_3}^{2-}$ flowing from the cathode through the anion exchange membrane to the anode. Depending on the species (OH^-, ${HCO_3}^-$, or ${CO_3}^{2-}$), there is a carbon/CO_2 crossover. For ${HCO_3}^-$, one CO_2 molecule per electron is transferred and $\tau = 1\,\frac{\mathrm{mol_{CO2}}}{\mathrm{mol}}$ (Eq. S12). Correspondingly for ${CO_3}^{2-}$, $\tau = 0.5\,\frac{\mathrm{mol_{CO2}}}{\mathrm{mol_{e-}}}$ (Eq. S12 + S13). For OH^-, $\tau = 0\,\frac{\mathrm{mol_{CO2}}}{\mathrm{mol_{e-}}}$. The dominant charge carrier can be determined experimentally by the molar ratio of CO_2 and O_2 in the anode outlet. We refer to Alerte et al. [2] for further details on the electrolyzer model and carbon crossover.

$$CO_2 + OH^- \rightleftharpoons {HCO_3}^- \tag{S12}$$

$$ {HCO_3}^- + OH^- \rightleftharpoons {CO_3}^{2-} + H_2O \tag{S13}$$

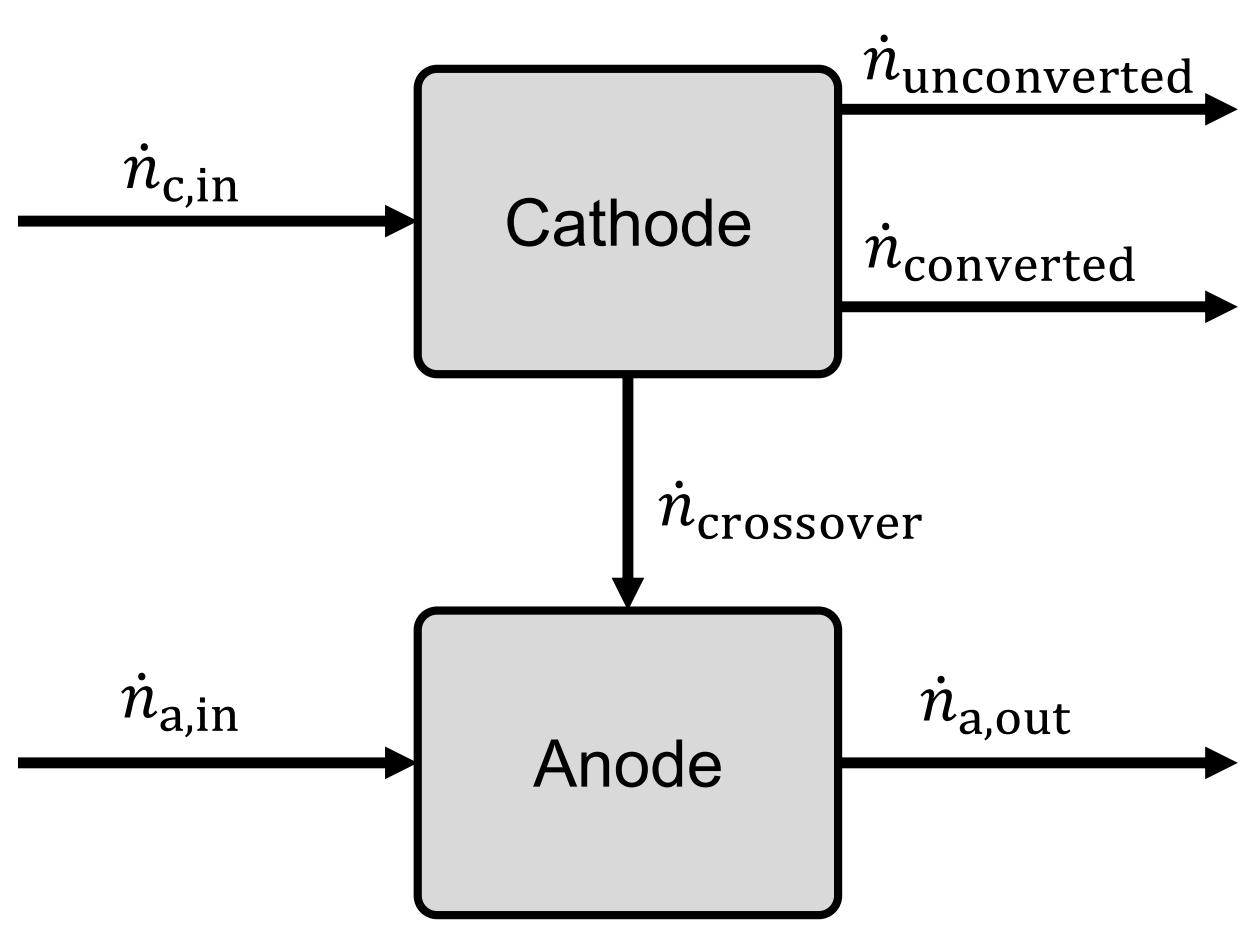

Figure S2: Electrolyzer model for CO_2 flows based on Alerte et al. [2].

We define a base case and an ideal case for assumptions on the electrolyzer performance. All parameters are provided in Table S2, where the base case is adopted from Alerte et al. [2]. The base case cathodic CO_2 conversion (63 %) and crossover ($0.5\,\frac{\mathrm{mol_{CO2}}}{\mathrm{mol}}$) correspond to about 25 % overall single pass CO_2 conversion (combined with the other assumptions on Faradaic efficiencies). The cell voltage includes the required thermodynamic minimum

voltage (about 1.15 V for ethylene [2, 9]) and an overvoltage [10]. In line with Alerte et al. [2], an anodic Faradaic efficiency of 100 % is assumed. The current density is kept constant for the base case and ideal case to limit degrees of freedom and is in the suggested range for optimal current densities (2000 $\frac{A}{m^2}$ to 5000 $\frac{A}{m^2}$ [11–13]). In the literature, lower voltages are reported than those cited by Alerte et al. [2] (3.9V) ($FE_{Ethylene}$ = 83.2 %, $FE_{Hydrogen}$ = 8.3 %, $FE_{Carbon\ monoxide}$ = 6.3 %, $FE_{Ethanol}$ = 2.5 %, 3.55 V cell voltage, 2500 $\frac{A}{m^2}$[14])($FE_{Ethylene}$ = 66 %, $FE_{Hydrogen}$ = 5 %, $FE_{Acetate}$ = 7 %, $FE_{Ethanol}$ = 12 %, 2.4 V cell voltage, 2750 $\frac{A}{m^2}$[15]). Hence, we decreased the voltage for the base case.

Table S2: Key assumptions on CO_2 electrolyzer performance. The values for the base case are oriented to the study by Alerte et al. [2].

Parameter	Unit	Base case	Ideal case
Cell voltage	V	2.5	1.4
Current density	$\frac{A}{m^2}$	2060	2060
$FE_{Ethanol}$	%	17	0
$FE_{Acetate}$	%	7	0
$FE_{n-Propanol}$	%	2	0
$FE_{Ethylene}$	%	50	100
$FE_{Carbon\ monoxide}$	%	16	0
$FE_{Hydrogen}$	%	5	0
Cathodic CO_2 conversion	%	63	63
Anodic CO_2 crossover	$\frac{mol_{CO2}}{mol_{e^-}}$	0.5	0.5
Ethylene to 1-butene conversion	%	93.3	93.3
Ethylene to n-butane conversion	%	5.3	5.3

The entering CO_2 stream to the electrolyzer is humidified. We assume 100 % CO_2 humidification [1, 16, 17] and referring to the importance of the humidification [18, 19]. Water formation is often neglected [20] at the electrolyzer outlet, but it is crucial because the outlet is in vapor-liquid equilibrium with the formed water, which influences downstream separation. Furthermore, Choi et al. [21] indicated that the origin of protons in ethylene can come from the anolyte and not from the humidification of the cathode inlet stream. Therefore, we assume that water is present in sufficient amounts and is in vapor-liquid equilibrium on the cathode and anode sides [21].

On the anode side, a potassium hydroxide solution is circulated as anolyte. To account for losses, we assume 100 $\frac{L}{m^2}$ and year for a 0.1 M solution [22]. The results on potassium demands are then also in order of magnitude as for the H_2 electrolyzer by Lee et al. [23].

S1.3. Ethylene dimerization to 1-butene

For the dimerization of ethylene to 1-butene (Ethylene dimerization in Figure S1), we assume an ethylene conversion of 93.3 % to 1-butene (Eq. S14) and 5.3 % to n-butane (Eq. S15) as byproduct based on experimental results [1]. As the focus of this work is the CO_2 electrolyzer, the dimerization is not investigated in detail. The dimerization reactor equipment is assumed to be negligible relative to the electrolyzer in the techno-economic analysis and the life cycle assessment. We refer to established processes [24, 25], experimental work [1, 26, 27], and further literature [27–38] for more details on the dimerization.

$$\text{1−Butene}: \quad 2C_2H_4 \longrightarrow C_4H_8 \tag{S14}$$

$$\text{n−Butane}: \quad 2C_2H_4 + H_2 \longrightarrow C_4H_{10} \tag{S15}$$

S1.4. Separation

Gas separation is an integral part of electrochemical CO_2 reduction processes, as the electrolyzer outlet streams contain unreacted CO_2, products, and multiple byproducts. The technology development for separating these streams is still ongoing [2, 5, 20, 39–42]. Early studies assessed potential technologies and calculated significant energy demands [2, 39, 41], however, the energy demand for separation has to be reduced for environmental and economic feasibility [39].

To the best of our knowledge, no fully established technology exists, but multiple technologies and models for such have been discussed. Here, we provide a short overview of technologies to explain the choices made in this work. An overview of separation technologies is given in Figure S3 and Table S3. Previous works used multiple approaches to estimate the cost and energy demands of gas separation systems. The approaches include assumptions of fixed separation cost based on correlations from literature [43, 44], the adaption of separation cost from bio-methane upgrade [12, 45], a combination of absorption, extraction, and distillation models [2, 7, 46–48], or the energy demand estimation based on minimum thermodynamic energy demand for separation [3, 49].

A well-established model assumes a fixed energy demand of $0.25\,\frac{\mathrm{kW\,h}}{\mathrm{m^3}}$ for the gas volume treated. A capital cost of 1 989 043 \$ for a capacity of $1000\,\frac{\mathrm{m^3}}{\mathrm{h}}$ is assumed and is scaled up with an exponent of 0.7 [45]. The foundation is the data from Paturska et al. [45] for biogas upgrade. Jouny et al. [12] applied assumptions from pressure-swing adsorption to electrolyzer systems, and the model is now widely used. However, currently no adsorbents are available

for the challenging CO_2/ethylene separation [20].

Sarswat et al. [5] fitted adsorption isotherms for the separation of gas mixtures. The authors combined single adsorption isotherms from literature for a specific separation task and estimated energy demands based on the heat of adsorption. To our knowledge, the model is the most detailed adsorption separation model in the field of process design for CO_2 electrolyzers. However, the application of the isotherms to varying compositions of the electrolyzer outlet steam is limited.

Membranes are also discussed in the literature for gas separation [5, 40]. Heßelmann et al. [40] describe a CO_2/carbon monoxide/H_2 membrane separation in detail. However, membrane separation is often designed for a specific separation task. The performance of multi-product electrolyzer outlet streams is not investigated in detail, which limits the application to the separation task in this work.

A higher technology readiness level is seen for absorption. CO_2 flue gas capture via absorption is an established process, and CO_2 can be captured via solvents like monoethanolamine [2]. The adaptation to CO_2 electrolyzers has been rigorously modeled by Alerte et al. [2] and Alerte et al. [7]. Current limiting factors are the potentially high solvent and energy demands for solvent recovery in distillation. The same energy limitations apply for low-temperature (cryogenic) distillation [2].

Due to limited data, the energy demand and cost intensity of gas separations have also been estimated based on fixed correlations. The simplest assumption is a fixed value for the separation of, e.g., $0.001\,\frac{\$}{\text{kg}}$ [43]. Fixed cost [44, 50, 51] and other correlations are also used in the literature [13]. However, this simplification is not considered in this work, as it might not reflect the correct order of magnitude for the cost of a multi-component separation, and the integration of the separation processes into heat integration, TEA, and LCA is not possible.

Motivated by thermodynamics, Wyndorps et al. [49] assumed the energy demand for separation as the minimum energy demand based on mixing entropy and the ideal gas law. Additionally, Moore et al. [3] reviewed the entropic efficiency of gas separation technologies and came up with an estimation of 7 % entropic efficiency as a conservative estimate [3].

The downstream separation system in this work is modeled for each individual unit operation. Due to missing information and the use of established technologies, assumptions are made. Generally, a 99 % and 1 % split of components in separation units is used if not

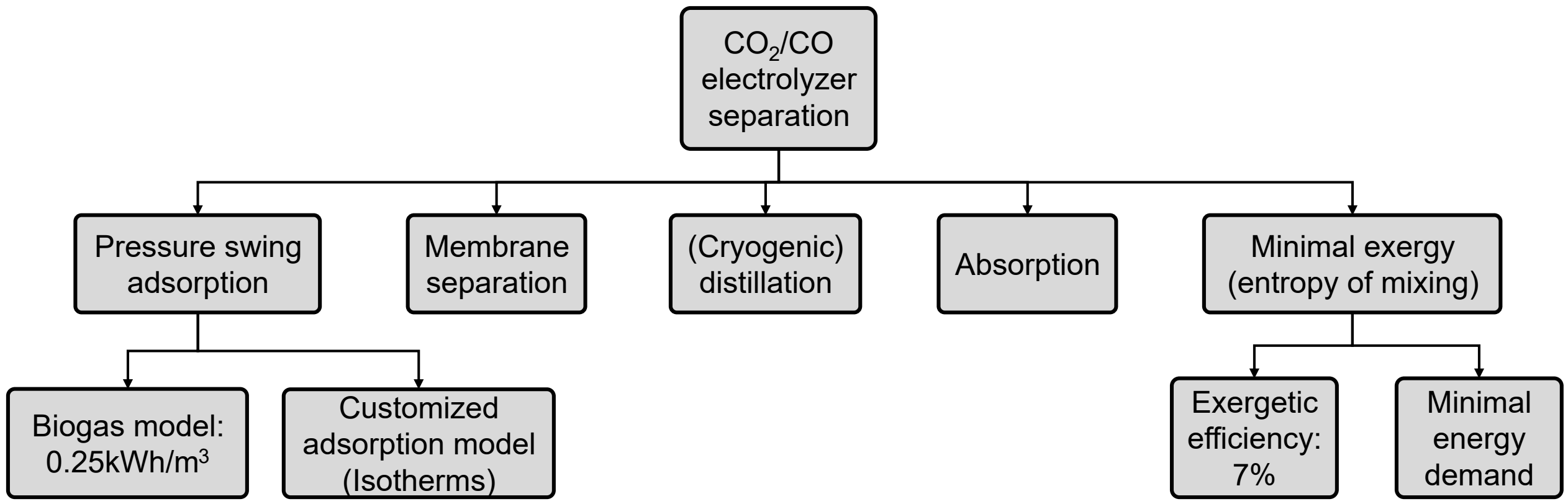

Figure S3: Separation technologies used in the literature for the downstream separation of gas mixtures from CO_2/carbon monoxide electrolyzers.

Table S3: Separaton technologies for the downstream separation of gas mixtures from CO_2/carbon monoxide electrolyzers.

Technology	Reference
Biogas PSA model [12, 45]	[52–55]
Customized adsorption models	[5, 20]
Membrane separation	[5, 20, 40]
(Cryogenic) distillation	[2, 5, 7, 20, 52]
Absorption	[2, 7, 20]
Entropic efficiency model [3]	[56]
Minimal energy demand	[49]

specified differently (97 % overall recycle efficiency [46]). Adsorptive dehumidification after the electrolyzer is used, based on experimental work by Lee et al. [1] [40, 57]. Liquid streams are separated using distillation. The 1-butene separation is similar to industrial separations with distillation units for the separation of lighter gases and heavier byproducts via two distillation columns [24, 33, 34]. For all other separations, we assume 7 % entropic efficiency for separation, as the assumption appears to be a conservative approximation for the electrolyzer downstream separation [3].

All separated byproducts are combusted to CO_2 and H_2O and recycled, as also done by Sarswat et al. [5]. O_2 from the anode is used for the combustion. Alternative use of the O_2 byproduct is not considered, and O_2 is vented [8, 40, 58–60] as the market demand too low. Further purification would also be needed, which is outside the scope of this study [59]. Details on the individual separations are provided below.

S1.4.1. Anode separation

For this work, we adopt the entropic separation model of Moore et al. [3] for the anode separation of CO_2 and O_2 (Anode separation in Figure S1). The separation is modeled as isothermal and isobaric, considering one inlet and two outlet streams. The energy demand is calculated as

$$W = \frac{RT}{\eta}\left[-\dot{n}_{\mathrm{in}}\left(\sum_i x_{\mathrm{in},i}\ln(x_{\mathrm{in},i})\right) + \sum_{j=\mathrm{out1,out2}} \dot{n}_{\mathrm{j}}\left(\sum_i x_{j,i}\ln(x_{j,i})\right)\right] \tag{S16}$$

with power demand W, entropic efficiency $\eta = 7\,\%$, molar flow $\dot{n}$, mole fraction x_i of component i. We assume the feasibility of the CO_2/O_2 separation based on multiple separation studies in the literature (air separation [61, 62], H_2/CO_2 separation [62], CO_2/carbon monoxide/O_2 separation [63], biogas upgrade [64], $CO_2/N_2/O_2$ separation [65]).

S1.4.2. H_2O separation via adsorptive dehumidification

Adsorptive dehumidification is used after the electrolyzers [40] with parameters by [57], which reflects the experimental setup by Lee et al. [1] (H_2O separation in Figure S1). The technology is seen as established [7, 66–75] and we assume an energy demand of 0.022 $\frac{\mathrm{kW\,h}}{\mathrm{m}^3}$ based on recalculation from cost from Heßelmann et al. [40] and Scholz et al. [57]. Only water is removed, since adsorptive dehumidification is also used in water-ethanol separation [68], with ethanol as the main byproduct in the electrolyzer, which is liquid under ambient conditions.

S1.4.3. Cathode liquid separation

To purify the cathode liquid stream, one distillation column is modeled (Liquid separation in Figure S1). The feed to the column is a liquid mixture of water, ethanol, 1-propanol, acetate, and trade of all other components in the process. The carbon-containing components are separated from water and fed into the combustion unit. A complete separation of water is not carried out due to the water-1-propanol azeotrope. Hence, some carbon losses occur with the water stream.

S1.4.4. 1-Butene separation

The 1-butene separation after the dimerization reactor consists of a 2-stage cryogenic distillation process, which is the standard in industry (Figure S4, Gas separation and 1-Butene separation in Figure S1) [24, 29, 33, 34]. Other options, such as extractive distillation [36], exist but are not considered in this work.

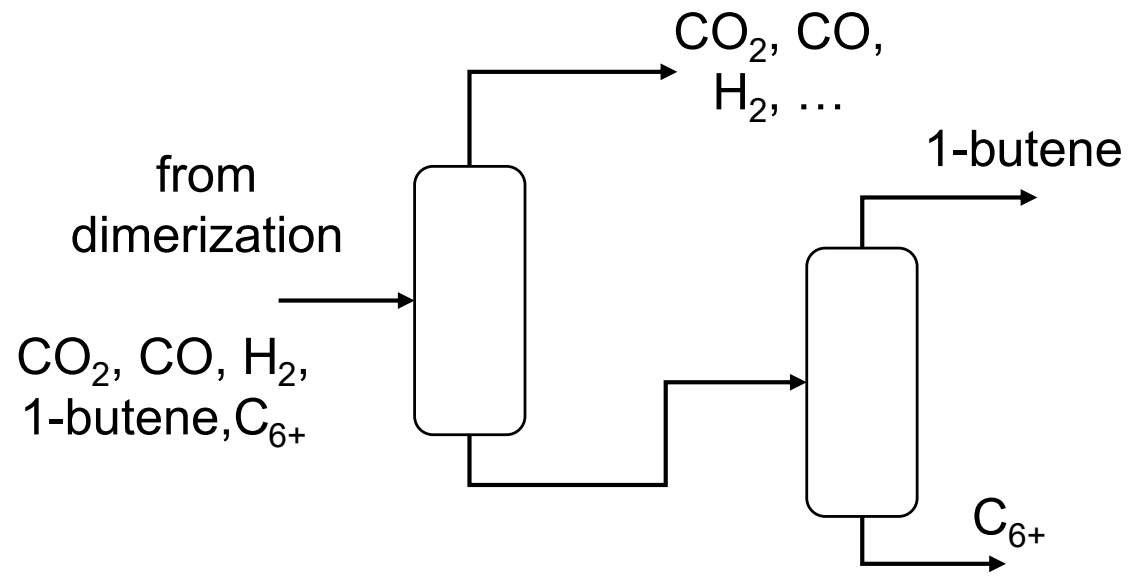

Figure S4: Distillation scheme for 1-butene separation based on the literature [24, 29, 33, 34].

S1.4.5. Combustion

All byproducts are combusted to CO_2 and H_2O (Combustion in Figure S1), as also suggested by Sarswat et al. [5] who feed methane to the combustion to achieve full utilization of the anode stream in the combustion. In this study, only part of the anode CO_2/O_2 stream is used for combustion while the other part is separated (Section S1.4.1). A stoichiometric amount of O_2 is provided for the combustion via the anode CO_2/O_2 mixture. This split of the anode stream is adjusted depending on combustion O_2 demand. The heat from the combustion is modeled as available at 250 °C or lower if the combustion outlet is cooled down to reach the electrolyzer input temperature.

The combustion mixture in this study is enriched with CO_2, prompting an evaluation of its flammability. As discussed by Li et al. [76] and Osterberg et al. [77], a minimum O_2 concentration of approximately 10 % is necessary for flammability, which is achieved in this study (10 mol % to 20 mol %). The combustion of mixtures with high inert gas content is feasible at elevated temperatures (1200 K–1800 K) [78, 79]. High CO_2 concentrations, furthermore, support the prevention of too high flame temperatures [80, 81]. Additionally, increased pressure reduces the limiting oxygen concentration [82], a trend observed across various hydrocarbons [77]. Beyond flammability, a catalyst within the combustion system can facilitate chemical conversion [83]. In this study, we assume the presence of a catalyst or increased pressure if necessary to overcome limitations posed by the limiting oxygen concentration [82].

The separate anode stream, which is mixed with the cathode stream and separation waste streams, allows control of the combustion by dosing O_2 as needed. Due to the low CO_2 conversion in the electrolyzer, the CO_2 concentration is high (57 mol % to 80 mol %), which acts as a buffer to prevent thermal runaway. Industrial applications of combustion with pure oxygen are established practice in oxyfuel combustion [84–86], and combustible mixtures are regularly handled in syngas, gasification, and purge gas treatment [87].

S1.5. O_2 byproduct

The O_2 from the anode separation is available as a purified stream but is not further considered in this study (O_2 in Figure S1). Typically, O_2 side streams are not considered and vented (for water electrolysis) [8, 40, 58–60]. For market usability, O_2 would need to be further purified and compressed (Market product as liquid from cryogenic separation with min. 99.6 wt% [88] compared to achieved purity in electrolyzer downstream 97.9 wt%). Furthermore, the production volume of oxygen might exceed market demand [59]. A comparison and credit for replacing fossil-based O_2 production can have a significant impact on the cost and climate change impacts of the process [7]. We observed significant climate change benefits when granting credit for replacing fossil-based O_2 production. However, such a credit appears to overestimate the benefit when the electrolyzer system is highly decarbonized relative to a fossil-based scenario. Therefore, we neglect the O_2 byproduct in subsequent economic and climate-change impact calculations.

S1.6. Heat integration

The energy streams and temperatures from the process simulation are used for the calculation of the utility demand of the process. By heat integration, the demand for heating and cooling utilities is reduced. The targets for heat integration are calculated using pinch analysis [89, 90]. Afterward, a heat exchanger network is designed to utilize the internal potential for heat integration.

The SYNHEAT formulation is used to mathematically formulate the heat exchanger design problem, resulting in a mixed-integer optimization problem [91]. The objective is to minimize utility demand and equipment costs for the process. The commercial solver KNITRO is used for solving the optimization problem [92, 93]. The problem is solved for every investigated flowsheet configuration.

The utility demand minimization is extended by adding an approximation of the equipment cost term to the objective function. By introducing a penalty for not meeting the pinch targets, the pinch targets are met first before minimizing the equipment cost (Equation S17).

$$
\begin{aligned}
\min_{\boldsymbol{x},\boldsymbol{y}} & \sum_{l \in \text{Utilities}} \left(Q_l \left(\text{price}_l + \text{Equip}_{\text{cost},Q,l} + M\right)\right) + \sum_{\substack{k \in \text{Internal} \\ Q \text{ Streams}}} \left(Q_k \text{Equip}_{\text{cost},Q,k}\right) \\
& + \sum_{l \in \text{Utilities}} \left(z_l \text{Equip}_{\text{cost},z,l}\right) + \sum_{\substack{k \in \text{Internal} \\ Q \text{ Streams}}} \left(z_k \text{Equip}_{\text{cost},z,k}\right) - M \left(Q_{\text{HU,Pinch}} + Q_{\text{CU,Pinch}}\right) \\
\text{s.t.} \; & \boldsymbol{h}(\boldsymbol{x},\boldsymbol{y}) = \boldsymbol{0} \\
& \boldsymbol{g}(\boldsymbol{x},\boldsymbol{y}) \leq \boldsymbol{0} \\
& \text{Equip}_{\text{cost},Q,l} = F_{\text{CAPEX}} \frac{b'}{U_{\text{heat}} \Delta T_l} \\
& \text{Equip}_{\text{cost},Q,k} = F_{\text{CAPEX}} \frac{b'}{U_{\text{heat}} \Delta T_k} \\
& \text{Equip}_{\text{cost},z,l} = F_{\text{CAPEX}} a \\
& \text{Equip}_{\text{cost},z,k} = F_{\text{CAPEX}} a \\
& \boldsymbol{x}_{\text{lb}} \leq \boldsymbol{x} \leq \boldsymbol{x}_{\text{ub}} \;\; \in \mathbb{R}^n \\
& \boldsymbol{y}_{\text{lb}} \leq \boldsymbol{y} \leq \boldsymbol{y}_{\text{ub}} \in \mathbb{N}_0^m
\end{aligned}
\tag{S17}
$$

In Equation S17, the variables describe the continuous decision variables x, binary decision variables y, utility and internal heat streams Q, utility prices price, equipment cost terms depending on the heat stream $\text{Equip}_{\text{cost},Q}$ or the a binary variable $\text{Equip}_{\text{cost},z}$, a penalty constant M, the utility pinch targets for heating $Q_{\text{HU,Pinch}}$ and cooling $Q_{\text{CU,Pinch}}$, a capital factor considering the Chemical Engineering Plant Cost Index and an annual capital charge ratio F_{CAPEX}, equipment cost parameters a and b', heat transfer coefficients U_{heat}, and temperature differences between utilities and process streams ΔT (compare Section S3). The equations $\boldsymbol{h}(\boldsymbol{x},\boldsymbol{y})$ and $\boldsymbol{g}(\boldsymbol{x},\boldsymbol{y})$ describe equality and inequality constraints on temperature feasibility, energy balances, and logical constraints from the SYNHEAT formulation [91]. A linear approximation of the equipment cost is used (Compare b' Equation S21).

For this work, a minimum approach temperature of $T_{\text{min}} = 10\,\text{K}$ is considered [94–96]. Furthermore, the heat demand for the direct air capture unit is assumed to be required at 79 °C (assumed based on Deutz and Bardow [97]). In scenarios where the required desorption temperature for direct air capture is elevated, a heat pump could provide the required thermal lift. The utilities and assumed temperatures are provided in Table S4. The temperature levels are based on the default utilities in Aspen HYSYS [98]. Additionally, a heat pump for

heat below 100 °C is considered [97].

The electrolyzer requires significant cooling due to overvoltage [99]. Cooling electrolyzers has been an industrially relevant topic for large-scale H_2 production electrolyzer [48, 100–102]. About 20 % to 40 % of electrical energy is waste heat in H_2 production [100, 103] which is even higher for CO_2 electroreduction. Hodges et al. [104] considered the cooling requirements in H_2 electrolysis at 80 °C in a techno-economic analysis, which is in line with our assumption.

For the cooling utilities, we assume an exergetic efficiency of 0.3 for the refrigeration system, which is on the order of magnitude reported in the literature [105, 106]. However, it is strongly dependent on the case study (but not the focus here). For electric boilers, an energetic efficiency of 99 % is considered [107].

Table S4: Utility information.

Utility	Supply temperature range	Electric efficiency
Heat Pump	100 °C to 100 °C	2.51 [97]
LP Steam	125 °C to 124 °C	0.99 [107]
MP Steam	175 °C to 174 °C	0.99 [107]
HP Steam	250 °C to 249 °C	0.99 [107]
Hot Oil	280 °C to 250 °C	0.99 [107]
Fired Heat (1000 °C)	1000 °C to 400 °C	0.99 [107]
Fired Heat (2000 °C)	2000 °C to 400 °C	0.99 [107]
Very high temperature	3000 °C to 2999 °C	0.99 [107]
Cooling water	10 °C to 15 °C	$25.25 \rightarrow 0.0396\,\frac{\mathrm{kW}}{\mathrm{kW}}$ [108]
Refrigerant 1	−25 °C to −24 °C	Exergetic eff. 0.3 [105, 106]
Refrigerant 2	−40 °C to −39 °C	Exergetic eff. 0.3 [105, 106]
Refrigerant 3	−65 °C to −64 °C	Exergetic eff. 0.3 [105, 106]
Refrigerant 4	−103 °C to −102 °C	Exergetic eff. 0.3 [105, 106]
Very low Temperature	−270 °C to −269 °C	Exergetic eff. 0.3 [105, 106]
Power	-	1

We calculate a minimum energy demand as a benchmark for comparison. The minimum voltage for CO_2 conversion to ethylene is $U_{\mathrm{min}} = 1.15\,\mathrm{V}$ [2, 9] . The corresponding minimum energy demand for the ethylene synthesis is $E_{\mathrm{min}} = 47.5\,\frac{\mathrm{GJ}}{\mathrm{t_{C2H4}}}$ (neglecting separation and CO_2 capture) [2]. As the dimerization reaction to 1-butene is exothermic, the minimum energy demand for the CO_2 electroreduction to ethylene can also be seen as a benchmark for the production of 1-butene via the proposed two-step process with $E_{\mathrm{min}} = 47.5\,\frac{\mathrm{GJ}}{\mathrm{t_{1-butene}}}$. For comparison, the minimum energy demand for the direct electrochemical 24-electron CO_2 reduction to 1-butene is $E_{\mathrm{min}} = 45.7\,\frac{\mathrm{GJ}}{\mathrm{t_{1-butene}}}$ [109–111].

S2. Details on the implementation and simulation

The calculations in this work are implemented in Aspen HYSYS and in Python. The process is simulated in Aspen HYSYS v11 [98], while the other calculations for heat integration, techno-economic analysis, and life cycle assessment are implemented in Python. The ENTRL-RK property model is used for all calculations except the 1-butene separation, for which the SR-POLAR model is used due to the higher pressure.[2] The energy streams in the electrolyzer (power demand, heat streams) are calculated in Aspen HYSYS [98] using mass and energy balances together with the fluid package. The electrolyzer is modeled using a reactor, separation, and spreadsheet units [112]. Modeling of KOH is neglected as numerical convergence of the trace component is challenging. The KOH demand is calculated separately based on the electrolyzer size (see Section S1.2 and [22]).

The automated data exchange between Aspen HYSYS and Python is facilitated by a software interface described in several examples [113–115]. The Pina package is used for the Pinch analysis [116]. The calculations for the life cycle assessment with the ecoinvent database [88] are implemented using the Brightway package [117] (Section S4).

S3. Methodological details on the techno-economic analysis

A techno-economic analysis (TEA) is performed based on the flowsheet simulation and heat integration results. The information used includes material and energy flows and estimates of equipment sizes to calculate operating and investment costs for a chemical plant. The TEA follows the economic estimation methods by Towler and Sinnott [96]. For some calculations, Towler and Sinnott [96] provide ranges for assumptions and parameters. In that case, the chosen values are adapted from Vázquez and Guillén-Gosálbez [94].

The minimum selling price MSP for the production of 1-butene is the economic key performance indicator in the TEA:

$$\mathrm{MSP} = \frac{\mathrm{ACCR} \cdot \mathrm{CAPEX} + \mathrm{OPEX}}{m_{1-\mathrm{butene}}} \tag{S18}$$

CAPEX describes the capital expenditures, which include investment costs for the process equipment. OPEX describes the operational expenditures, which include costs for raw materials, utilities, and fixed operational costs. $m_{1-\mathrm{butene}}$ is the mass of 1-butene produced. For this work, we refer to the minimum selling price, assuming no minimum profit margin. Therefore, the minimum selling price equals the total annualized cost, which is also used in the literature [96].

An annual capital charge ratio ACCR of 0.2 is assumed [94]. All capital expenditures are

estimated as the sum of inside battery limits ISBL, offsite cost OSBL, capital for engineering CE, capital for contingency CC, and working capital costs CW (Eq. S19).

$$\text{CAPEX} = \text{ISBL} + \text{OSBL} + \text{CE} + \text{CC} + \text{CW}, \tag{S19}$$

The sum of all unit costs UC_u of the set of process units U is the ISBL.

$$\text{ISBL} = \sum_{u \in U} \text{UC}_u \tag{S20}$$

Correlations from literature are used for the cost of each unit [96].

$$\text{UC}_u = k(a + b \cdot C^n)\frac{\text{CEPCI}^{\text{current}}}{\text{CEPCI}^{2010}} \quad \forall u \in U \tag{S21}$$

In Eq. S21, C is a characteristic design variable and the parameters k,a,b, and n depend on the unit type. The parameters for equation S21 are provided in Table S6 Towler and Sinnott [96]. The Chemical Engineering Plant Cost Index CEPCI is used to adjust the parameters for price increases relative to a reference year (Table S5). In this work, all economic calculations are based on the year 2023. The plant lifetime is estimated to 20 yr [118].

Table S5: Chemical Engineering Plant Cost Index.

Year	CEPCI	Reference
2023	803.4	[119]
2018	603.1	[120]
2010	532.9	[96]

Table S6: UC parameters from Towler and Sinnott [96].

Unit	Design variable	k	a	b	n
Heat exchanger	Area in m^2	1.0	28 000	54	1.20
Trays	Diameter in m	1.0	130	440	1.80
Compressor	Duty in MW	2.5	580 000	20 000	0.60

The CAPEX parameters are taken from Vázquez and Guillén-Gosálbez [94] [96].

$$\text{OSBL} = 0.35 \cdot \text{ISBL} \tag{S22}$$

$$\text{CE} = 0.20 \cdot (\text{ISBL} + \text{OSBL}) \tag{S23}$$

$$\text{CC} = 0.30 \cdot (\text{ISBL} + \text{OSBL}) \tag{S24}$$

$$\text{CW} = 0.15 \cdot (\text{ISBL} + \text{OSBL}) \tag{S25}$$

The operational expenditures OPEX are made up of variable costs of operation VOC and fixed costs of operation FOC. Furthermore, an annual operation time of 8600 $\frac{\text{h}}{\text{yr}}$ is assumed for the plant.

$$\text{OPEX} = \text{FOC} + \text{VOC} \tag{S26}$$

For the fixed cost of operation holds (Eq. S27)

$$\text{FOC} = C^{\text{Salary}} + C^{\text{Superv}} + C^{\text{SalOv}} + C^{\text{Maint}} + C^{\text{Land}} + C^{\text{Ins}} + C^{\text{GenOv}}. \tag{S27}$$

The fixed cost of operation comprises costs for salary, supervision, salary overhead, maintenance, land, taxes and insurance, and general overhead [94, 96]. The salary cost is estimated via labor costs for operators with the yearly operator salary S, the number of shifts for full-time operation NS, and the number of operators per shift OS (Table S7).

$$C^{\text{Salary}} = S \cdot \text{NS} \cdot \text{OS} \tag{S28}$$

All other fixed operating costs are scaled by the salary or investment costs (Eq. S31).

$$C^{\text{Superv}} = 0.25 \cdot C^{\text{Salary}} \tag{S29}$$
$$C^{\text{SalOv}} = 0.45 \cdot (C^{\text{Salary}} + C^{\text{Superv}}) \tag{S30}$$
$$C^{\text{Maint}} = 0.03 \cdot \text{ISBL} \tag{S31}$$
$$C^{\text{Land}} = 0.01 \cdot (\text{ISBL} + \text{OSBL}) \tag{S32}$$
$$C^{\text{Ins}} = 0.01 \cdot \text{ISBL} \tag{S33}$$
$$C^{\text{GenOv}} = 0.65 \cdot (C^{\text{Salary}} + C^{\text{Superv}} + C^{\text{SalOv}} + C^{\text{Maint}}) \tag{S34}$$

Table S7: FOC parameters [94].

Parameter	Value
S	$40\,000\ \frac{\$_{2023}}{\text{operator}}$
NS	3 shifts
OS	$5\ \frac{\text{operator}}{\text{shift}}$

Feedstocks, utilities, and waste treatment incur variable costs that scale with plant size (Eq. S35).

$$\text{VOC} = C^{\text{Feed}} + C^{\text{Util}} + C^{\text{Waste}} \tag{S35}$$

The variable costs contain the annual cost of feedstocks C^{Feed}, the annual cost of utilities C^{Util}, and the cost of waste treatment C^{Waste}. A summary of the main assumptions for the techno-economic analysis is provided in Table S8.

A plant size of 40 kt 1-butene annual production [108] is assumed which is in the range of smaller ethylene plants and large enough that economies of scale are expected to be utilized [7].

Electricity price of 0.03 $\frac{\$_{2023}}{\text{kW h}}$ in range or field with multiple references in electrolyzer TEA in range of 0.01 $\frac{\$_{2023}}{\text{kW h}}$ to 0.05 $\frac{\$_{2023}}{\text{kW h}}$ [3, 7, 12, 20, 22, 47, 54, 129]. Furthermore, the price is below the threshold 0.04 $\frac{\$}{\text{kW h}}$ needed to make electrolyzers competitive [43]. Electricity prices for technologies and countries are taken from Vatankhah Ghadim et al. [130] and Glo [131].

After the heat exchanger network design is completed, the required equipment is sized. The heat transfer area is calculated using a fixed heat transfer coefficient of 0.16 $\frac{\text{kW}}{\text{m}^2\,\text{K}}$ (Towler and Sinnott [96] for heat transfer between gases and water).

Furthermore, distillation columns are sized. The sizing is based in Towler and Sinnott [96] using the volumetric flows at the top and bottom and a stage height of 0.5 m.

The capital expenditures for the separation units, estimated using entropic efficiency, are neglected due to missing data. Furthermore, the capital expenditures of the utility system are neglected.

The following capital expenditures are assumed for the adsorptive dehumidification unit [40, 57].

$$\text{CAPEX}_{\text{ads.dehum.}} = \frac{7636.7\,\$}{0.8971\left(\frac{\text{m}^3}{\text{h}}\right)^{0.16}} \cdot \frac{\text{CEPCI}_{2023}}{\text{CEPCI}_{2013}} \cdot \dot{V}^{0.16} \tag{S36}$$

Both the CO_2 capture plant and the CO_2 electrolyzer are subject to intensive research, and technology development is expected [10, 132]. The technology development and corresponding reductions in investment costs are accounted for using learning rates [121, 132]. We assume the investment cost decreases with increasing ethylene Faradaic efficiency, defining the ethylene Faradaic efficiency as the design parameter, assuming a higher technology rollout with higher ethylene Faradaic efficiency. In the literature, the cumulative design capacity is often considered a design parameter for learning rate, but it is not available for this specific case study [121, 132].

The CAPEX_i for the DAC plant and the CO_2 electrolyzer are calculated as

$$\text{CAPEX}_i = \text{CAPEX}_{i,\text{FE}=50\,\%} \cdot \left(\frac{\text{FE}_{\text{Ethylene}}}{50\,\%}\right)^{-b_i} \tag{S37}$$

with the learning parameter b_i and the reference to the $\mathrm{CAPEX}_{i,\mathrm{FE}=50\,\%}$ for an ethylene Faradaic efficiency of 50 %, which corresponds to the base case of this study. The experience rate ER_i is calculated using the learning parameter b_i.

$$\mathrm{ER}_i = 1 - 2^{-b_i} \tag{S38}$$

We define capital expenditures for the base case of FE = 50 % and the ideal case of FE = 100 %. The learning parameters are then calculated to match both cost assumptions.

$$b_i = -\frac{\ln\left(\frac{\mathrm{CAPEX}_{i,\mathrm{FE}=100\,\%}}{\mathrm{CAPEX}_{i,\mathrm{FE}=50\,\%}}\right)}{\ln\left(\frac{100\,\%}{50\,\%}\right)} \tag{S39}$$

All other equipment considered in this study is treated as established and mature (heat exchangers, compressors, distillation columns, etc.), and specific learning rates are not considered.

Table S8: Main economic assumptions for the techno-economic analysis.

Variable	Value	Comment	Reference
CO_2 capture investment cost	$80\,\frac{\$_{2023}}{t_{CO2}}$ to $50\,\frac{\$_{2023}}{t_{CO2}}$	Direct air capture and projected CO_2 price from literature	Assumption based on Young et al. [121], Dan [122]
Electrolyzer investment cost	$450\,\frac{\$}{kW}$ to $200\,\frac{\$}{kW}$		[22, 123]
Electricity price	$0.03\,\frac{\$_{2023}}{kW\,h}$		[3, 12, 54, 124]
Cooling water price	$0.03\,\frac{€_{2018}}{m^3}$		[125]
Wastewater treatment	$1.5\,\frac{€_{2018}}{m^3}$		[125]
Potassium hydroxide	$1.5\,\frac{€_{2018}}{m^3}$		[125]
Plant lifetime	20 yr		
Annual operation time	$8600\,\frac{h}{yr}$		
Electrolyzer lifetime	10 yr	Assumption based on Moore et al. [3]	
Annual capital charge ratio	0.2		[94]
Price conversion from 2018 to 2023	$\frac{PPI_{2023}}{PPI_{2018}} = \frac{360.217}{290.158}$	Cooling water and wastewater treatment converted to $\$_{2023}$ via Producer Price Index (PPI) for Chemical Manufacturing (average for 2023 and 2018)	[126, 127]
Fossil 1-butene reference price	$1.09\,\frac{\$_{2023}}{kg_{1-butene}}$– $1.87\,\frac{\$_{2023}}{kg_{1-butene}}$		[128]

S4. Methodological details on the life cycle assessment

A life cycle assessment (LCA) is performed for the CO_2 electroreduction and ethylene dimerization process [133, 134]. Methodological details on the LCA are given in this section, which complements the description in the main manuscript.
The functional unit is 1 kg 1-butene. The system boundaries consider the provision of raw materials, process operation, and end-of-life combustion (Figure S5). Data for the background system is taken from the ecoinvent 3.10 database using the cutoff system model [88].

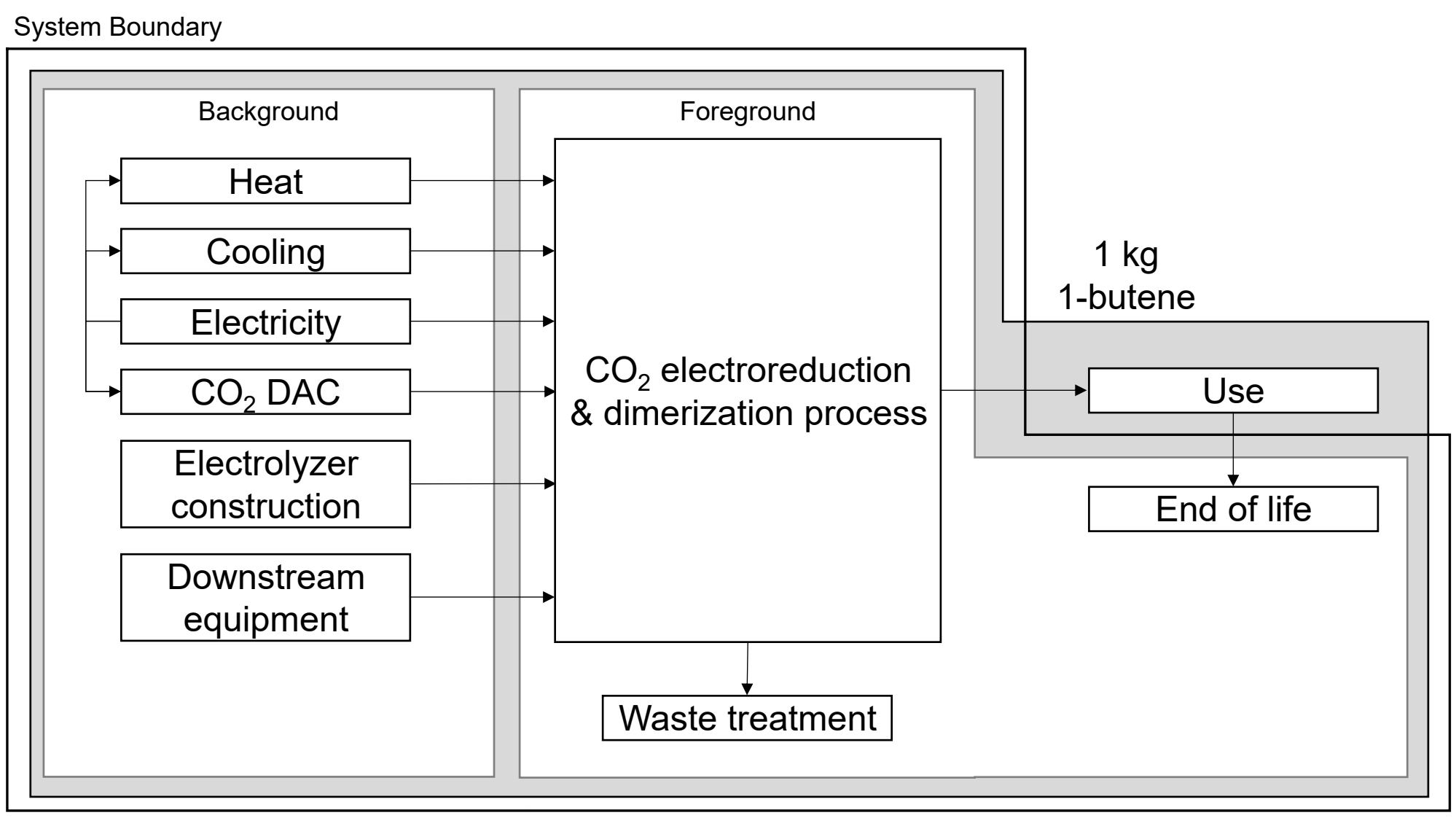

Figure S5: Cradle-to-gate and end-of-life system boundaries for the life cycle assessment system of CO_2 electroreduction and dimerization process. While the foreground system is modeled in detail, the data for the background system is taken from available databases and further from the literature.

The feedstock CO_2 is assumed to be provided via an adsorbent-based direct air capture plant. The reference is a 4 $\frac{\text{kt}}{\text{yr}}$ plant by Deutz and Bardow [97], which is scaled linearly to the required demand of the CO_2 electroreduction process. The adsorbent is approximated using inventory data from Madhu et al. [135]. The heat and power demand for direct air capture is met via heat integration and assumed utilities.
The electricity supply as a process utility is assumed to be provided by a decarbonized electricity mix (45 % wind, 34 % solar, 13 % nuclear, 5 % hydro, and 3 % other sources, according to the ReEDS Mid-Case 100 % by 2035 scenario) located in the US [136]. Generally, inventory data for the US is assumed for energy and material supply if available from ecoinvent. Otherwise, global averages are considered.
The process utilities (Table S4) are evaluated based on their respective electricity demand during operation. Cooling water is modeled using life cycle inventory data from the literature

for cooling water provision [108] and the electricity demand for pumping [94].
The vaporous waste streams are modeled as flared to CO_2 and H_2O. The streams are vented, and heat recovery is neglected.
The main assumptions for LCA are provided in Table S10.

Table S10: Main assumptions for the life cycle assessment of the CO_2 electroreduction and ethylene dimerization process.

Activity	Assumption
CO_2 source	Direct air capture [97]
Electricity source	Decarbonized US electricity mix [136]
Fossil 1-butene reference	Global 1-butene market [88]

Distillation columns and heat exchangers are modeled via the steel demand. The mass of steel is calculated using a density of steel of $\rho_{\text{Steel}} = 7500\,\frac{\text{kg}}{\text{m}^3}$. The walls are modeled with a thickness of $\delta = 0.002\,\text{m}$ [137]. Given the column size and the heat transfer area of the heat exchangers, the total mass is calculated. The total mass is estimated as twice the mass of the heat transfer area A for the heat exchangers (Eq. S40).

$$m_{\text{HeatExchanger}} = \rho_{\text{Steel}} \cdot A \cdot \delta \cdot 2 \tag{S40}$$

The mass is split equally between Chromium steel for the heat transfer area and unalloyed steel for the surroundings and piping.
The assumption on the heat exchanger mass is validated using data from the literature for industrial heat exchangers, which is in line with the suggestion by Gerber et al. [138]. The areas and weights of industrial heat exchangers are provided in Table S11. The above-mentioned assumption of twice the mass of the heat transfer area is consistent with the literature data. A linear curve fit to the data in Table S11 results in a slope of about 28 $\frac{\text{kg}}{\text{m}^2}$. Given the assumptions about steel density and wall thickness, the estimate serves as a solid approximation, assuming no differentiation between steel types.

Compressor equipment is modeled using data from Peng et al. [142] for a natural gas compressor. Following the method suggested by Gerber et al. [138], the life cycle inventory is scaled linearly with power due to limited data availability.
The inventories for the utility equipment and unspecified separation units are neglected.

The construction of the electrolyzer is a rarely investigated topic in the literature. We found that only a few studies have examined the life cycle assessment of electrolyzers, and even fewer of CO_2 electrolyzers. We collected a few studies in Table S12.

Table S11: Heat exchanger data from the literature for comparison.

Area m^2	Weight kg	Reference
15.23	242	[137]
18.6	193.8	[139]
29.51	468	[137]
60	2000	[140]
88.7	1407	[137]
102.6	647	[141]
120	8000	[140]
125	6000	[140]
150	6000	[140]
380	10 000	[140]
450	20 000	[140]
800	12 000	[140]
900	30 000	[140]

Table S12: Details on inventories for electrolyzer construction.

Reference	Electrolyzer type	Details
Bareiß et al. [58]	PEM water electrolysis	Detailed inventory for stack, and balance of plant Germany / Europe for an optimal future case for a 1 MW stack which is scaled to 1 kW and 1 m^2 (with 18.5 $\frac{kW}{m^2}$) (cited by Löffelholz et al. [143])
Koj et al. [60]	Alkaline water electrolysis /AEL)	Detailed inventory for stack, and balance of plant for a prospective pressurized 6 MW electrolyzer which is scaled to 1 kW and 1 m^2 (with 4 cells, 139 cells each, and 1.6 m^2). Inventories for an inverter unit are taken from ecoinvent (global average [88]).
Schreiber et al. [144]	High-temperature solid oxide electrolysis for syngas production	Detailed inventory for cells, stack, and balance of plant including transport and direct emissions in Germany / Europe for a 150 kW stack which is scaled to 1 kW and 1 m^2 (with 2 $\frac{W}{cm^2}$)

Table S12: Details on inventories for electrolyzer construction. (continued)

Reference	Electrolyzer type	Details
Rumayor et al. [145]	PEM CO_2 electrolyzer for formic acid production	Detailed inventory for electrodes, membrane, and assembly for a 10 cm^2 cell which is scaled to 1 kW and 1 m^2 (with 200 $\frac{mA}{cm^2}$ and 4.3 V)
Lee et al. [1]	Anion exchange membrane CO_2 electrolyzer for ethylene production	Inventory for an experimental 5 cm^2 cell which is scaled to 1 kW and 1 m^2 (with 100 $\frac{mA}{cm^2}$ and 2.5 V). Only the metal loading of anode and cathode is considered from Lee et al. [1]. Membrane and cathode carbon paper inventories taken from Rumayor et al. [145]. No construction or steel plates were considered.
Mori et al. [146]	PEM fuel cell	Detailed inventory for stack of an proton exchange membrane fuel cell for a 48 kW stack in Europe which is scaled to 1 kW and 1 m^2 (with 280 membrane electrode assembly units with an active area of 200 cm^2 each)
Stropnik et al. [147]	PEM fuel cell	Detailed inventory for materials, manufacturing, and balance of plant of a proton exchange membrane fuel cell for a 1 kW system in Europe/Global which is scaled to 1 kW and 1 m^2 (with 0.13 $\frac{W}{cm^2}$ [148])

Table S12: Details on inventories for electrolyzer construction. (continued)

Reference	Electrolyzer type	Details
ecoinvent [88]	PEM fuel cell	Inventories for a future 2 kW stack polymer electrolyte membrane (PEM) fuel cell. Averaged as global market activity considering 6 yr lifetime and building materials, their decomposition, the energy consumption during production, and land use [88]. The inventory is scaled to 1 kW and 1 m^2 (with 160 cm^2 and 2772 W) assuming the Lunar Power Plant Element case from Giacoppo et al. [149].

In this study, we use the pilot-plant scale inventory by Koj et al. [60] as an assumption with a higher technology readiness level [150] and scaled per $kW_{installed}$ (alignment with techno-economic analysis (Section S3)). The construction of all electrolyzers in Table S12 is modeled for comparison and to assess the importance of the choice made.

Similar to the techno-economic analysis, we assume a technology development of CO_2 electrolyzer and carbon capture equipment (Section S3 and Eqs. S37, S38, S39). The same proportional changes are used for the TEA and the material and energy consumption of the CO_2 electrolyzer and DAC construction following the approach of Qiu et al. [151].

The Levelized Cost of Carbon Abatement (LCCA) is calculated to compare the cost of CO_2 utilization technologies on an economic scale [152–154] (Eq. S41).

$$\text{LCCA} = \frac{\text{MSP}_{\text{ECO2RR}} - \text{MSP}_{\text{fossil}}}{E_{\text{fossil}} - E_{\text{ECO2RR}}} \tag{S41}$$

$\text{MSP}_{\text{ECO2RR}}$ is the minimum selling price of the electrochemical route, while $\text{MSP}_{\text{fossil}}$ describes fossil reference as market price (average). E_{fossil} and E_{ECO2RR} give the climate change impacts of the fossil reference and the climate change impacts of the electrochemical route, respectively.

The ecoinvent processes used for the inventories discussed above are listed in Table S13. Mainly, US datasets are selected if available. For many materials, global average inventories are chosen due to missing regional data in ecoinvent [88]. Some selected processes vary in location and/or are very similar. The processes were selected as close as possible to the provided inventories (especially for the direct air capture plant and the electrolyzer construction). Hence, variances arise in assumptions, proxy processes, or locations.

Table S13: Life cycle inventory data for the streams in the process and assumptions on the background system.

Process stream	(ecoinvent) activity	Reference unit	Comment	Reference
CO_2 feedstock	CO_2 direct air capture (DAC) process	kg	Electricity mix for this study assumed	[97]
Electricity for process operation, utility supply, and DAC	Electricity mix	kW h	US	[136]
Heating utility	Electric heating using an electric boiler and heat pump	MJ	Assume only electricity mix for electricity supply	See Table S4
Cooling water	Pump electricity demand and cooling water provision	MJ	Assume pressure drop in heat exchangers for power demand and cooling water provision from literature	[94, 155]
Refrigeration	Electricity demand for operation	MJ	Assume only electricity mix for electricity supply	See Table S4
Gaseous purge stream	Flaring to CO_2 and H_2O and direct emission	kg	Assume no heat utilization and direct emission	
Wastewater treatment	Treatment of wastewater, unpolluted, wastewater treatment, RoW	m^3	Assume unpolluted wastewater as the purity after the distillation column is high	[88]

Table S13: Life cycle inventory data for the streams in the process and assumptions on the background system. (continued)

Process stream	(ecoinvent) activity	Reference unit	Comment	Reference
Stainless steel for vessels, heat exchangers, compressors	market for steel, chromium steel 18/8, GLO	kg	See assumptions in text for steel use	[88]
Unalloyed steel for heat exchangers	Market for steel, unalloyed, GLO	kg	See assumptions in text for steel use	[88]
1-Butene	Market for butene, mixed, RoW	kg		[88]
Acetic anhydride	Market for acetic anhydride, GLO	kg		[88]
Acetone	Market for acetone, liquid, RoW	kg		[88]
Acrylonitrile	market for acrylonitrile, GLO	kg		[88]
Acrylonitrile-butadiene-styrene copolymer	Market for acrylonitrile-butadiene-styrene copolymer, GLO	kg		[88]
Activated carbon	Market for activated carbon, granular, GLO	kg		[88]
Alloy steel	Market for steel, low-alloyed, GLO	kg		[88]
Aluminium oxide	Market for aluminium oxide, metallurgical, RoW	kg		[88]

Table S13: Life cycle inventory data for the streams in the process and assumptions on the background system. (continued)

Process stream	(ecoinvent) activity	Reference unit	Comment	Reference
Aluminium waste landfill	Treatment of waste aluminium, sanitary landfill, RoW	kg		[88]
Aluminium, cast alloy	Market for aluminium, cast alloy, GLO	kg		[88]
Ammonium sulfate	Market for ammonium sulfate, RoW	kg		[88]
Aniline	Market for aniline, RoW	kg		[88]
Barium carbonate	Market for barium carbonate, GLO	kg		[88]
Benzyl alcohol	Market for benzyl alcohol, GLO	kg		[88]
Borax	Market for borax, anhydrous, powder, GLO	kg		[88]
Boric oxide	Market for boric oxide, GLO	kg		[88]
Calendering	Market for calendering, rigid sheets, GLO	kg		[88]
Car transport	Transport, passenger car, large size, diesel, EURO 5, RoW	km		[88]
Carbon black	Market for carbon black, GLO	kg		[88]
Carbon monoxide	Market for carbon monoxide, RoW	kg		[88]
Carboxymethyl cellulose	Market for carboxymethyl cellulose, powder, GLO	kg		[88]
Cast iron	Cast iron production, RoW	kg		[88]

Table S13: Life cycle inventory data for the streams in the process and assumptions on the background system. (continued)

Process stream	(ecoinvent) activity	Reference unit	Comment	Reference
Cast iron	Market for cast iron, GLO	kg		[88]
Cerium oxide	Market for cerium oxide, GLO	kg		[88]
Chemicals, general	Market for chemical, organic, GLO	kg		[88]
Chromium	Chromium production, RoW	kg		[88]
Chromium oxide	Market for chromium oxide, flakes, GLO	kg		[88]
Chromium steel production	Steel production, electric, chromium steel 18/8, RoW	kg		[88]
Chromium steel rolling	Sheet rolling, chromium steel, RoW	kg		[88]
Chromium steel, hot rolled	Market for steel, chromium steel 18/8, hot rolled, GLO	kg		[88]
Cobalt	Cobalt production, GLO	kg		[88]
Cobalt hydroxide	Market for cobalt hydroxide, GLO	kg		[88]
Concrete	Market group for concrete, medium strength, GLO	m^3		[88]
Concrete landfill	Treatment of waste concrete, inert material landfill, RoW	kg		[88]
Construction hall building	Building construction, hall, steel construction, RoW	m^2		[88]

Table S13: Life cycle inventory data for the streams in the process and assumptions on the background system. (continued)

Process stream	(ecoinvent) activity	Reference unit	Comment	Reference
Construction work, heat and power co-generation unit	Construction work, heat and power co-generation unit, 160kW electrical, construction work, heat and power co-generation unit, 160kW electrical, RoW	unit		[88]
Copper oxide	Market for copper oxide, GLO	kg		[88]
Copper, anode	Market for copper, anode, GLO	kg		[88]
Copper, cathode	Market for copper, cathode, GLO	kg		[88]
Diethyl ether	Ethylene hydration, RoW	kg		[88]
Electricity, mix	Market (group) for electricity, high voltage	kW h	US, BR, EU, CH	[88]
Electricity production, wind	Electricity production, wind, <1MW turbine, onshore, US	kW h		[88]
Electricity production, solar	Electricity production, solar thermal parabolic trough, 50MW, US	kW h		[88]
Electricity production, nuclear	Electricity production, nuclear, pressure water reactor, US	kW h		[88]

Table S13: Life cycle inventory data for the streams in the process and assumptions on the background system. (continued)

Process stream	(ecoinvent) activity	Reference unit	Comment	Reference
Electricity production, hydro	Electricity production, hydro, reservoir, alpine region, US	kW h		[88]
Electricity production, natural gas	fElectricity production, natural gas, combined cycle power plant, US	kW h		[88]
Electronic component	Electronic component production, active, unspecified, GLO	kg		[88]
Ethanol	Market for ethanol, without water, in 99.7% solution state, from ethylene, RoW	kg		[88]
Ethoxylated alcohol	Market for ethoxylated alcohol (AE11), GLO	kg		[88]
Ethylene glycol	Market for ethylene glycol, RoW	kg		[88]
Fatty acid	Market for fatty acid, GLO	kg		[88]
Fuel cell	Market for fuel cell, polymer electrolyte membrane, 2kW electrical, future, GLO	unit		[88]
Furnace heat	Heat production, natural gas, at industrial furnace low-NOx ¿100kW, RoW	MJ		[88]
Gadolinium oxide	Market for gadolinium oxide, GLO	kg		[88]

Table S13: Life cycle inventory data for the streams in the process and assumptions on the background system. (continued)

Process stream	(ecoinvent) activity	Reference unit	Comment	Reference
Glass fibre	Market for glass fibre, GLO	kg		[88]
Glass fibre reinforced plastic	Market for glass fibre reinforced plastic, polyamide, injection moulded, GLO	kg		[88]
Graphite	Market for graphite, GLO	kg		[88]
Heat from light fuel oil	Heat production, light fuel oil, at boiler 100kW condensing, non-modulating, RoW	MJ		[88]
Heat from natural gas	Market for heat, from steam, in chemical industry, RoW	MJ		[88]
Heat in industrial sites	Market for heat, district or industrial, natural gas, RoW	MJ		[88]
Hydrochloric acid	Market for hydrochloric acid, without water, in 30% solution state, RoW	kg		[88]
Industrial machine	Market for industrial machine, heavy, unspecified, RoW	kg		[88]
Inert waste landfill	Treatment of inert waste, inert material landfill, RoW	kg		[88]
Insulation	Tube insulation production, elastomere, RoW	kg		[88]

Table S13: Life cycle inventory data for the streams in the process and assumptions on the background system. (continued)

Process stream	(ecoinvent) activity	Reference unit	Comment	Reference
Inverter	Inverter production, 500kW, RoW	unit		[88]
Inverter	Market for inverter, 2.5kW, GLO	unit		[88]
Isopropanol	Market for iso-propanol, RoW	kg		[88]
Lanthanum oxide	Market for lanthanum oxide, GLO	kg		[88]
Lorry transport	Market for transport, freight, lorry, unspecified, RoW	t km		[88]
Lubricating oil	Market for lubricating oil, RoW	kg		[88]
Manganese	Manganese production, RoW	kg		[88]
Manganese dioxide	Market for manganese dioxide, GLO	kg		[88]
Metal working, average	Metal working, average for copper product manufacturing, RoW	kg		[88]
Methyl ethyl ketone	Market for methyl ethyl ketone, RoW	kg		[88]
Mineral wool landfill	Treatment of waste mineral wool, inert material landfill, RoW	kg		[88]
Monoethanolamine	Market for monoethanolamine, GLO	kg		[88]
Multi-storey building	Building construction, multi-storey, RoW	m^3		[88]

Table S13: Life cycle inventory data for the streams in the process and assumptions on the background system. (continued)

Process stream	(ecoinvent) activity	Reference unit	Comment	Reference
Nickel	Market for nickel, class 1, GLO	kg		[88]
Nitric acid	Narket for nitric acid, without water, in 50% solution stat, RNA	kg		[88]
N-methyl-2-pyrrolidone	Market for N-methyl-2-pyrrolidone, GLO	kg		[88]
Paint	Market for alkyd paint, white, without solvent, in 60% solution state, RoW	kg		[88]
Plaster mixing	market for plaster mixing, GLO	kg		[88]
Platinum	Market for platinum, GLO	kg	Approximation for iridium as also done by Nuss and Eckelman [156]	[88]
Polyethylene terephthalate	market for polyethylene terephthalate, granulate, amorphous, GLO	kg		[88]
Polyethylene, high density	Market for polyethylene, high density, granulate, GLO	kg		[88]
Polyphenylene sulfide	Market for polyphenylene sulfide, GLO	kg		[88]
Polypropylene	Market for polypropylene, granulate, GLO	kg		[88]
Polysulfone	Market for polysulfone, GLO	kg		[88]

Table S13: Life cycle inventory data for the streams in the process and assumptions on the background system. (continued)

Process stream	(ecoinvent) activity	Reference unit	Comment	Reference
Polyurethane adhesive	Market for polyurethane adhesive, GLO	kg		[88]
Polyurethane incineration	Treatment of waste polyurethane, municipal incineration, RoW	kg		[88]
Polyvinylchloride	Market for polyvinylchloride, suspension polymerised, GLO	kg		[88]
Potassium hydroxide	Market for potassium hydroxide, GLO	kg		[88]
Quicklime	Market for quicklime, in pieces, loose, RoW	kg		[88]
Reinforcement steel disposal	Treatment of waste reinforcement steel, collection for final disposal, RoW	kg		[88]
Reinforcement steel recycling	Treatment of waste reinforcement steel, recycling, RoW	kg		[88]
Reinforcing steel	Market for reinforcing steel, GLO	kg		[88]
Rubber	Synthetic rubber production, RoW	kg		[88]
Rubber incineration	Treatment of waste rubber, unspecified, municipal incineration, RoW	kg		[88]

Table S13: Life cycle inventory data for the streams in the process and assumptions on the background system. (continued)

Process stream	(ecoinvent) activity	Reference unit	Comment	Reference
Sheet rolling, aluminium, GLO	Market for sheet rolling, aluminium, GLO	kg		[88]
Sheet rolling, aluminium, RoW	Sheet rolling, aluminium, RoW	kg		[88]
Sheet rolling, copper, RoW	Sheet rolling, copper, RoW	kg		[88]
Ship transport	Market for transport, freight, sea, container ship, GLO	t km		[88]
Silica sand	Market for silica sand, GLO	kg		[88]
Silicone	Market for silicone product, RoW	kg		[88]
Sodium hydroxide	Market for sodium hydroxide, without water, in 50% solution state, GLO	kg		[88]
Sodium silicate	Market for sodium silicate, solid, RoW	kg		[88]
Sodium sulfate	Sodium sulfate production, from natural sources, RoW	kg		[88]
Sodium tetrahydridoborate	Brown-Schlesinger process, GLO	kg		[88]
Spent solvent	Market for spent solvent mixture, RoW	kg		[88]

Table S13: Life cycle inventory data for the streams in the process and assumptions on the background system. (continued)

Process stream	(ecoinvent) activity	Reference unit	Comment	Reference
Steam, chemical industry	Steam production, in chemical industry, RoW	kg		[88]
Stone wool	Market for stone wool, packed, GLO	kg		[88]
Strontium carbonate	Market for strontium carbonate, GLO	kg		[88]
Sulfuric acid	Sulfuric acid production, RoW	kg		[88]
Terephthalic acid	Market for purified terephthalic acid, GLO	kg		[88]
Tetrafluoroethylene	Market for tetrafluoroethylene, GLO	kg		[88]
Tin	Market for tin, GLO	kg		[88]
Titanium	Market for titanium, GLO	kg		[88]
Train transport	Market for transport, freight train, US	t km		[88]
Unalloyed steel	Market for steel, unalloyed, GLO	kg		[88]
Vinyl acetate	Vinyl acetate production, RoW	kg		[88]
Wastewater treatment, average	Treatment of wastewater, average, wastewater treatment, RoW	m^3		[88]
Wastewater treatment, unpolluted	Treatment of wastewater, unpolluted, wastewater treatment, RoW	m^3		[88]
Water, decarbonized	Market for water, decarbonised, US	kg		[88]

Table S13: Life cycle inventory data for the streams in the process and assumptions on the background system. (continued)

Process stream	(ecoinvent) activity	Reference unit	Comment	Reference
Water, deionized	Market for water, deionised, RoW	kg		[88]
Water, tap	Market group for tap water, GLO	kg		[88]
Yttrium oxide	Market for yttrium oxide, GLO	kg		[88]
Zinc oxide	Market for zinc oxide, GLO	kg		[88]
Zirconium oxide	Market for zirconium oxide, GLO	kg		[88]
CO_2 (fossil) to air	Carbon dioxide, fossil to air	kg		[88]
CO_2 from air	Carbon dioxide, in air	kg		[88]
CO_2 (biogenic) to air	Carbon dioxide, non-fossil	kg		[88]
H_2O to air	Water to air	kg		[88]
CO (fossil) to air	Carbon monoxide, fossil to air	kg		[88]
Nitrogen oxides	Nitrogen oxides	kg		[88]
NMVOC	NMVOC	kg		[88]
HFC-134a	1,1,1,2-Tetrafluoroethane	kg		[88]
Particulates, $< 2.5\,\mu m$	Particulates, < 2.5 um	kg		[88]
Land occupation	Occupation, industrial area, GLO	$m^2\,yr$		[88]

Table S13: Life cycle inventory data for the streams in the process and assumptions on the background system. (continued)

Process stream	(ecoinvent) activity	Reference unit	Comment	Reference
Land transformation from unspecified	Transformation, from unspecified	m^2		[88]
Land transpormation to industrial area	Transformation, to industrial area	m^2		[88]

S5. Utility systems in literature

Multiple studies on CO_2 electroreduction provide techno-economic analyses, including utility costs. Most cost assumptions are based on literature and chemical engineering textbooks. Table S14 provides a non-comprehensive overview of assumed heating utility costs compared to the assumed electricity prices.

Most studies assume NG-based steam cost, which reflects the current situation in many chemical sites [157–161]. Some of these studies provide more details and assume NG-based steam, which is also accounted for in an LCA.

Many studies do not disclose the source of heat used. However, comparing the assumed prices for heat and electricity shows that fossil heating is assumed, since a heat pump achieving near- or above-Carnot efficiency would be required for electricity-based heating.

For these comparisons, we used the Carnot efficiency of a heat pump defined as

$$\eta_{\text{Carnot,HeatPump}} = \frac{T_{\text{Hot}}}{T_{\text{Hot}} - T_{\text{Cold}}} \tag{S42}$$

We assume a heat pump temperature lift from 25 °C.

Table S14: Utility systems CO_2 electroreduction TEA studies.

Reference	Heating system	Price comparison utility (U) vs. electricity-driven heat pump (HP) (if data available) $\left[\frac{\$}{\text{GJ}}\right]$
Alerte et al. [7]	Cost estimations based on literature books, which provide current chemical industry data (NG / fossil-based) for LPS, MPS, HPS, and natural gas.	LPS:<br>U: 4.54, HP: 0.89<br>MPS:<br>U: 3.51, HP: 1.28<br>HPS:<br>5.66, HP: 1.43
Al Moinee et al. [162]	Fossil-based MPS with utility cost below electricity cost for heat pump [162].	MPS:<br>U: 3.19, HP: 6.44

Table S14: Utility systems CO_2 electroreduction TEA studies. (continued)

Reference	Heating system	Price comparison utility (U) vs. electricity-driven heat pump (HP) (if data available) $\left[\frac{\$}{\mathrm{GJ}}\right]$
Bahnamiri et al. [163]	Cost estimations based on literature books, which provide current chemical industry data (NG / fossil-based) [163]. HPS costs below the electricity cost for a heat pump.	LPS:<br>U: 4.30, HP: 4.09<br>HPS:<br>U: 4.30, HP: 6.58
Chung et al. [39]	Differentiation between electricity and steam with a higher carbon footprint for steam [39].	
Gao et al. [47]	HPS cost assumed from Bahnamiri et al. [163] based on current fossil-based steam. For the carbon footprint accounting, only renewable electricity is considered [47]. Utility costs are below the electricity costs for the heat pump.	HPS:<br>U: 4.36, HP: 4.77
Huang et al. [164]	Fossil-based heat assumption with natural gas [164].	
Pribyl-Kranewitter et al. [46]	Fossil steam from combined heat and power unit [46].	
Sarswat et al. [5]	Combined CO_2 electroreduction and combustion of NG [5].	
Silveira Sbrice Pinto et al. [165]	Fossil-based steam prices assumed based on current industrial reports and decarbonized electricity directly assumed for heating in LCA [165].	LPS:<br>U: 9.64, HP: 6.27<br>MPS:<br>U: 12.64, HP: 8.29<br>HPS:<br>12.64, HP: 11.70

Table S14: Utility systems CO_2 electroreduction TEA studies. (continued)

Reference	Heating system	Price comparison utility (U) vs. electricity-driven heat pump (HP) (if data available) $\left[\frac{\$}{GJ}\right]$
Venkataraman et al. [48]	Steam from natural gas assumed and considered for CO_2 emissions [48].	

[a] Assumption of latent heat for steam condensation: 2200 $\frac{kJ}{kg}$ [96]

[b] Default assumed temperatures low-pressure steam (LPS): 134 °C [96]

[c] Default assumed temperatures mid-pressure steam (MPS): 212 °C [96]

[d] Default assumed temperatures high-pressure steam (HPS): 250 °C [96]

S6. Results

S6.1. Investment cost and climate change impacts of the electrolyzer construction in the literature.

The investment cost of electrolyzers, often referred to as capital expenditures in the literature, is a subject of ongoing debate within the research community, characterized by significant uncertainty yet holding considerable importance [166–168]. Moreover, the scaling references used in these studies vary, with some expressing costs in dollars per kilowatt installed $\left(\frac{\$}{\mathrm{kW_{installed}}}\right)$ and others in dollars per square meter installed $\left(\frac{\$}{\mathrm{m^2_{installed}}}\right)$. To ensure the robustness of our analysis, we compare our assumptions against those reported in the literature. The assumed costs for electrolyzer investments, as presented in the literature, are illustrated in Figure S6a and Figure S6b, which depict data for CO_2 electrolyzers and other electrolyzer types with higher technology readiness levels (TRL) [150].

The data presented in Figure S6a and Figure S6b shows a wide variation in costs, spanning several magnitudes for both $\frac{\$}{\mathrm{kW_{installed}}}$ and $\frac{\$}{\mathrm{m^2_{installed}}}$, with values ranging from 100 $\frac{\$}{\mathrm{kW_{installed}}}$–8945 $\frac{\$}{\mathrm{kW_{installed}}}$ and 677 $\frac{\$}{\mathrm{m^2_{installed}}}$–20 000 $\frac{\$}{\mathrm{m^2_{installed}}}$, respectively. There are multiple reasons for these differing cost assumptions. Some studies rely on cost estimations for mature H_2O electrolyzers to estimate costs for CO_2 electrolyzers, often incorporating optimistic projections for future cost reductions. For example, current stack costs for H_2 electrolyzers range from 384 $\frac{€}{\mathrm{kW}}$ to 1071 $\frac{€}{\mathrm{kW}}$ for proton exchange membrane (PEM) setups, with potential reductions to 63 $\frac{€}{\mathrm{kW}}$ to 234 $\frac{€}{\mathrm{kW}}$ in the future [167]. Additionally, the type of electrolyzer significantly influences costs. For instance, alkaline electrolyzers typically have lower costs than more advanced systems, such as PEM and solid oxide electrolyzer cells (SOECs), which require higher investment [39]. In membrane electrode assembly (MEA) setups, the anode catalyst, particularly iridium, is a major cost contributor [10, 54].

Furthermore, the methods used for techno-economic analysis [169, 170] and the locations of studies vary widely. Inflation adjustments are sometimes made using the Chemical Engineering Plant Cost Index based on the year of publication, but this adjustment is not consistently applied. Given the inherent uncertainty, we have chosen not to adjust for inflation in our plot, as even adjusting for inflation from 2018 to 2023 (the range of our references) does not account for the observed orders-of-magnitude differences. Other factors contributing to cost variability include the estimation of economies of scale in electrolyzer production [99, 168]. Additionally, the uncertainty in cost data for H_2O electrolyzers, as discussed by Glenk and Reichelstein [166], is further compounded when extrapolating high TRL uncertainties to lower TRL CO_2 electrolyzers.

The high uncertainty in cost estimates highlights the significant impact of investment costs on the overall cost, as detailed in Section S6.4. There remains an open question within

the community regarding how to accurately estimate costs, as assumptions vary widely among studies, both in how they provide cost estimates and in how they use them for techno-economic analysis (TEA) calculations. In our study, we assume an installed cost of $450\,\frac{\$}{\mathrm{kW_{installed}}}$ to $200\,\frac{\$}{\mathrm{kW_{installed}}}$ [22, 123], given that we maintain a constant current density while varying voltage significantly. This approach reflects the assumed case more reasonably. The overarching question for the community remains how to estimate costs and whether to use scaling metrics of $\frac{\$}{\mathrm{kW_{installed}}}$ or $\frac{\$}{\mathrm{m^2_{installed}}}$.

The environmental impacts of electrolyzer construction are assessed using data from the literature. Due to the variability in available data, a brief overview is provided. References for life cycle inventories of different electrolyzer systems are listed [1, 58, 60, 88, 144–147], with details available in Table S12. The life cycle assessment for electrolyzer construction utilizes the ecoinvent database for all materials, with locations normalized to US data where available (see Table S13). Due to limited data availability, which is less comprehensive than cost estimations, system boundaries vary. These can range from focusing solely on active electrode materials to including data from pilot plants, which cover manufacturing, assembly inventories, and balance-of-plant components such as pumps, heat exchangers, and converters. Despite variations in electrolyzer applications—such as fuel cells, H_2 production, or CO_2 electroreduction—the basic structure of the electrochemical cell, including the cathode, anode, and membrane, remains similar across all cases. Similar to investment costs, normalization is performed on either $\frac{\$}{\mathrm{kW_{installed}}}$ or $\frac{\$}{\mathrm{m^2_{installed}}}$.

The climate change impacts associated with electrolyzer construction are illustrated in Figure S6, presented in terms of $\frac{\mathrm{kg_{CO2eq.}}}{\mathrm{kW_{installed}}}$ and $\frac{\mathrm{kg_{CO2eq.}}}{\mathrm{m^2_{installed}}}$. The observed differences in climate change impacts span several orders of magnitude, ranging from $46\,\frac{\mathrm{kg_{CO2eq.}}}{\mathrm{kW_{installed}}}$–$1415\,\frac{\mathrm{kg_{CO2eq.}}}{\mathrm{kW_{installed}}}$ and $118\,\frac{\mathrm{kg_{CO2eq.}}}{\mathrm{m^2_{installed}}}$–$245\,224\,\frac{\mathrm{kg_{CO2eq.}}}{\mathrm{m^2_{installed}}}$. The limited number of data points prevents the identification of a clear trend by electrolyzer type. The high variance across studies can be partly attributed to the technology readiness level of the assessed plants. For instance, for the publication of Lee et al. [1], we only considered the active materials of the cathode, anode, and membrane, excluding other parts and the balance of plant, resulting in impacts that fall in the middle range. Conversely, mature pilot plants with comprehensive inventories, such as the 1 MW pilot plant studied by Bareiß et al. [58] and the 48 kW pilot plant examined by Mori et al. [146], cover the entire range of impacts observed.

The materials used in electrolyzers, particularly iridium for the anode, are critical due to their scarcity and high climate change impacts associated with their extraction [171, 172]. The high impacts are particularly concerning given that other scarce metals, such as platinum, are also used in electrolyzers [172] and contribute significantly to climate change impacts during extraction [156]. Additionally, high-density cells have substantial per-square-meter

impacts, and variations in cell voltage and current density can lead to significant differences in climate change impacts. For instance, the climate change impact can range from 2.5 $\frac{kW}{m^2}$–173 $\frac{kW}{m^2}$, though such data are not always readily available.

When decarbonizing the chemical industry, the environmental impacts associated with electrolyzer construction could decrease if the supply chain for the required materials is also decarbonized. However, there is a critical need to quantify these impacts more precisely and to provide reliable inventories to better assess the potential of electrochemical processes for decarbonization. An overarching question remains: How should we assume inventories and determine the appropriate scaling metric, whether in $\frac{\$}{kW_{installed}}$ or $\frac{\$}{m^2_{installed}}$?

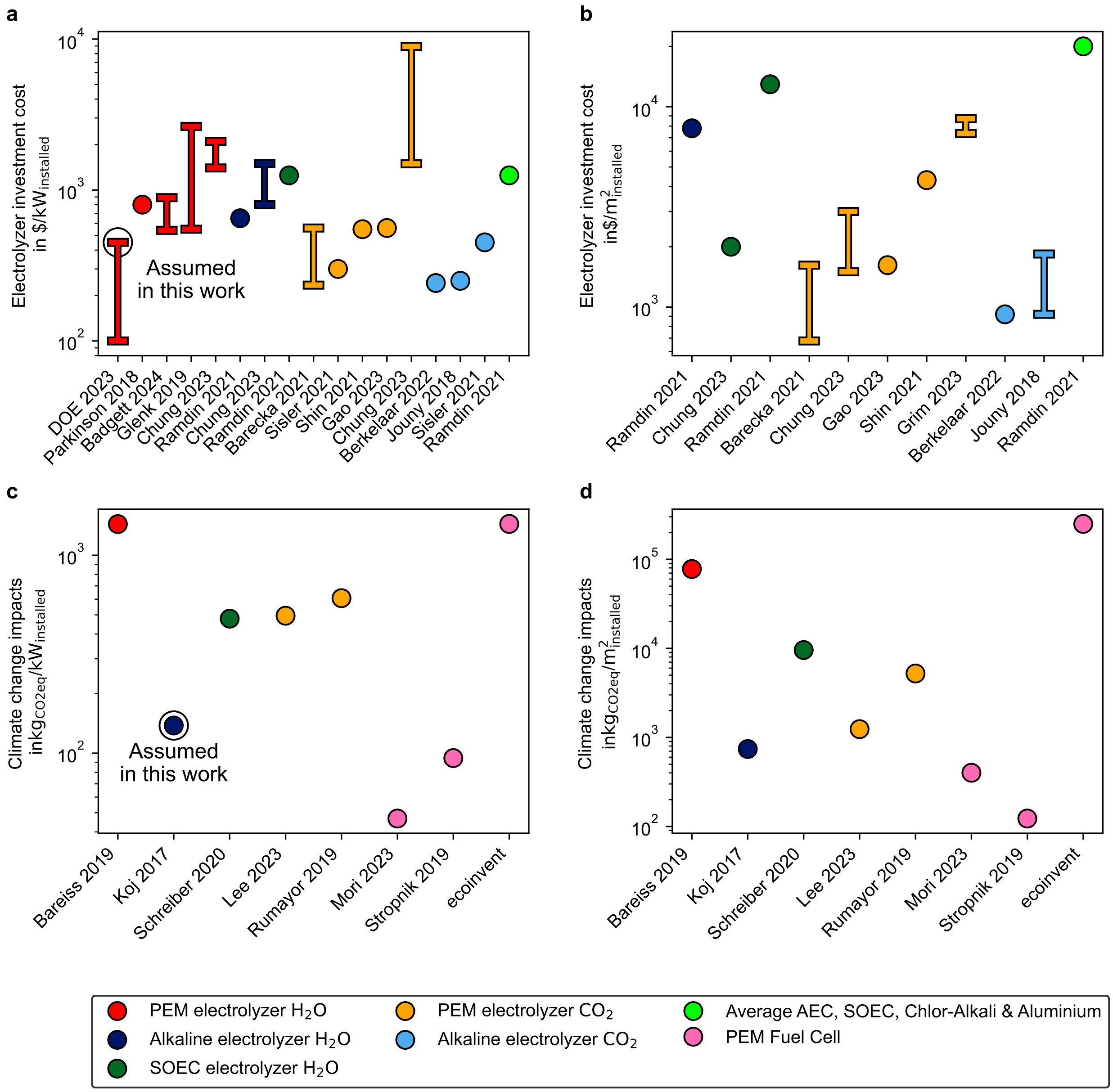

Figure S6: Investment cost for the installation of different electrolyzer configurations from literature based on (a) installed power ($kW_{installed}$) and (b) installed area ($m^2_{installed}$). Climate change impacts for the installation of different electrolyzer configurations from literature based on (c) installed power ($kW_{installed}$) and (d) installed area ($m^2_{installed}$). References: US DOE(2023) [123], Parkinson et al. (2018) [173], Badgett et al. (2024) [174], Glenk and Reichelstein (2019) [166], Chung et al. (2023) [39], Ramdin et al. (2021) [20], Jouny et al. (2018) [12], Shin et al. (2021) [54], Gao et al. (2023) [175], Sisler et al. (2021) [22], Barecka et al. (2021) [99], Berkelaar et al. (2022) [176], Grim et al. (2023) [129], ecoinvent [88], Bareiß et al. (2019) [58], Koj et al. (2017) [60], Schreiber et al. (2020) [144], Rumayor et al. (2019) [145], Lee et al. (2023) [1], Mori et al. (2023) [146], Stropnik et al. (2019) [147]. The assumed values for this study are marked with a circle.

S6.2. Sankey diagrams for the ideal case

The Sankey diagrams in Figure S7 and Figure S8 show the energy and carbon flows in the process for a low electrolyzer operating temperature, respectively. The figures supplement the results in Figure 1 in the main manuscript.

The overall electricity demand is 258 $\frac{\text{GJ}}{\text{t}_{1-\text{butene}}}$ for the base case and 93 $\frac{\text{GJ}}{\text{t}_{1-\text{butene}}}$ for the ideal case, compared to the minimum energy demand of 47.5 $\frac{\text{GJ}}{\text{t}}$ of 1-butene, which corresponds to the enthalpy of formation of ethylene from CO_2 and H_2O [2, 9].

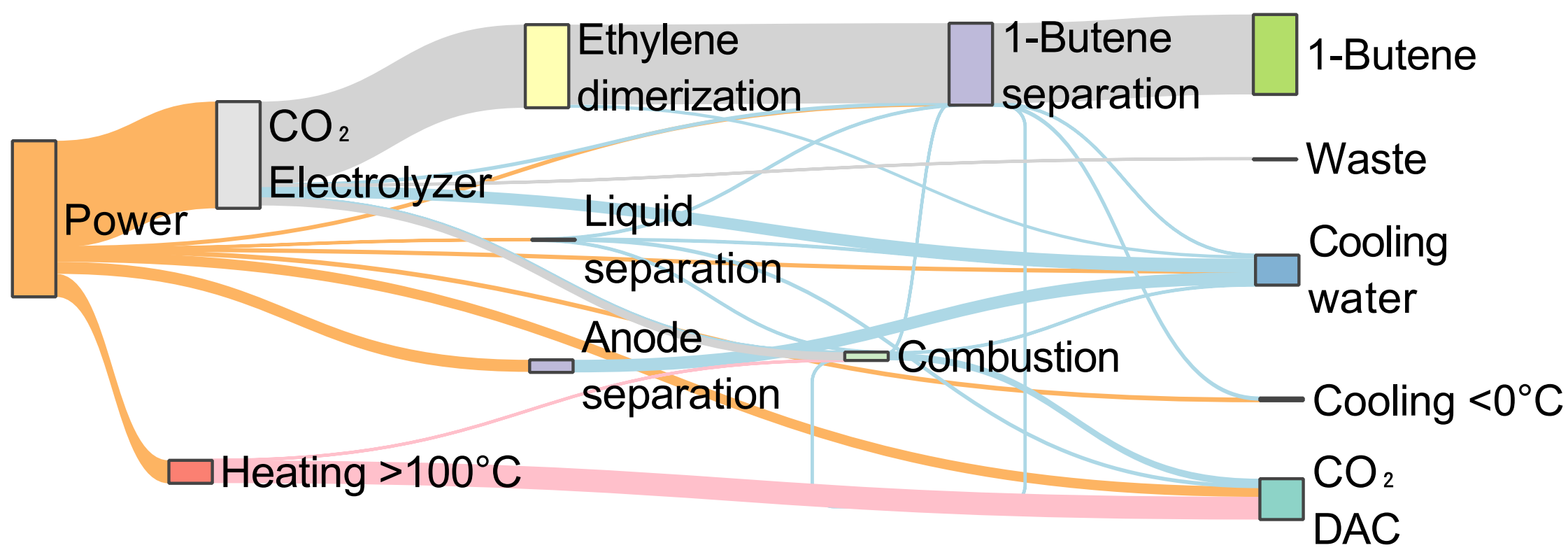

Figure S7: Sankey diagram of the energy flows in the electrochemical reduction of CO_2 to ethylene and dimerization to 1-butene for the ideal case.

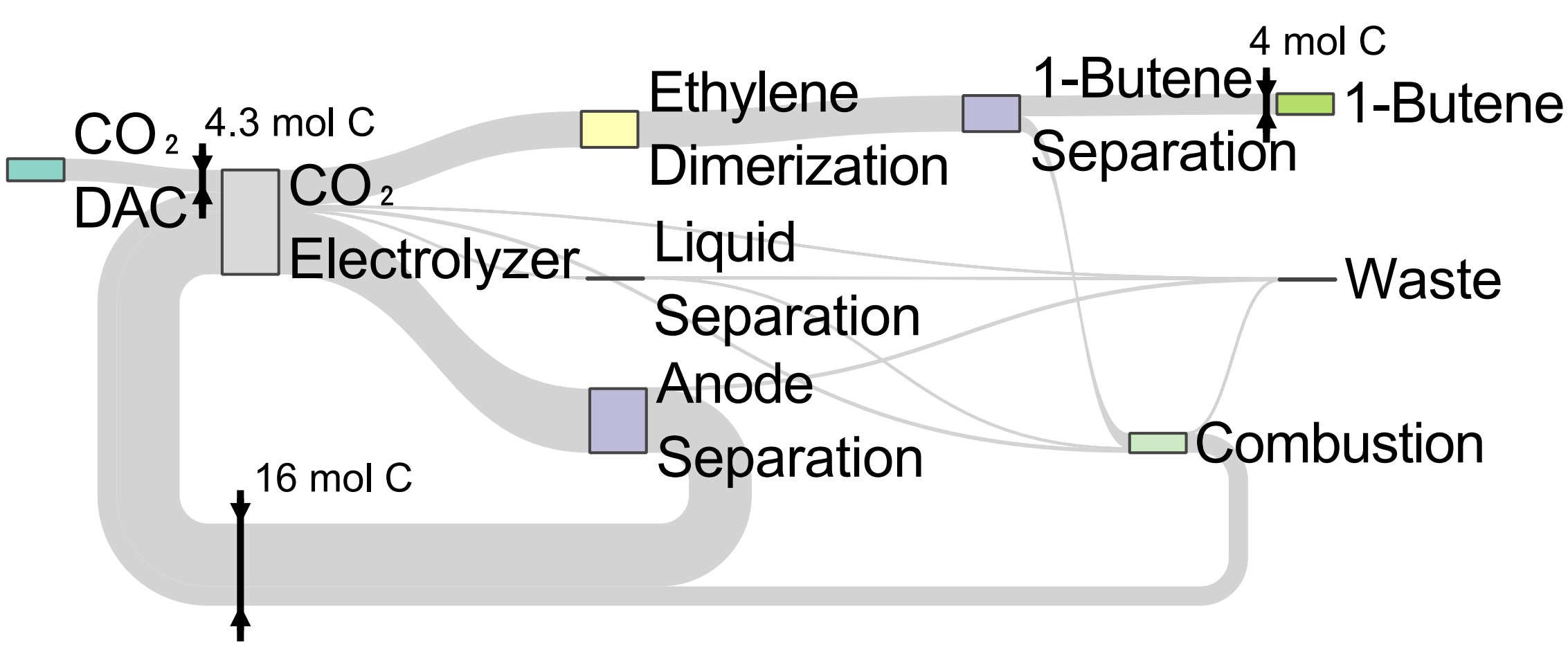

Figure S8: Sankey diagram of the carbon flows in the electrochemical reduction of CO_2 to ethylene and dimerization to 1-butene for the ideal case.

S6.3. Thermal energy

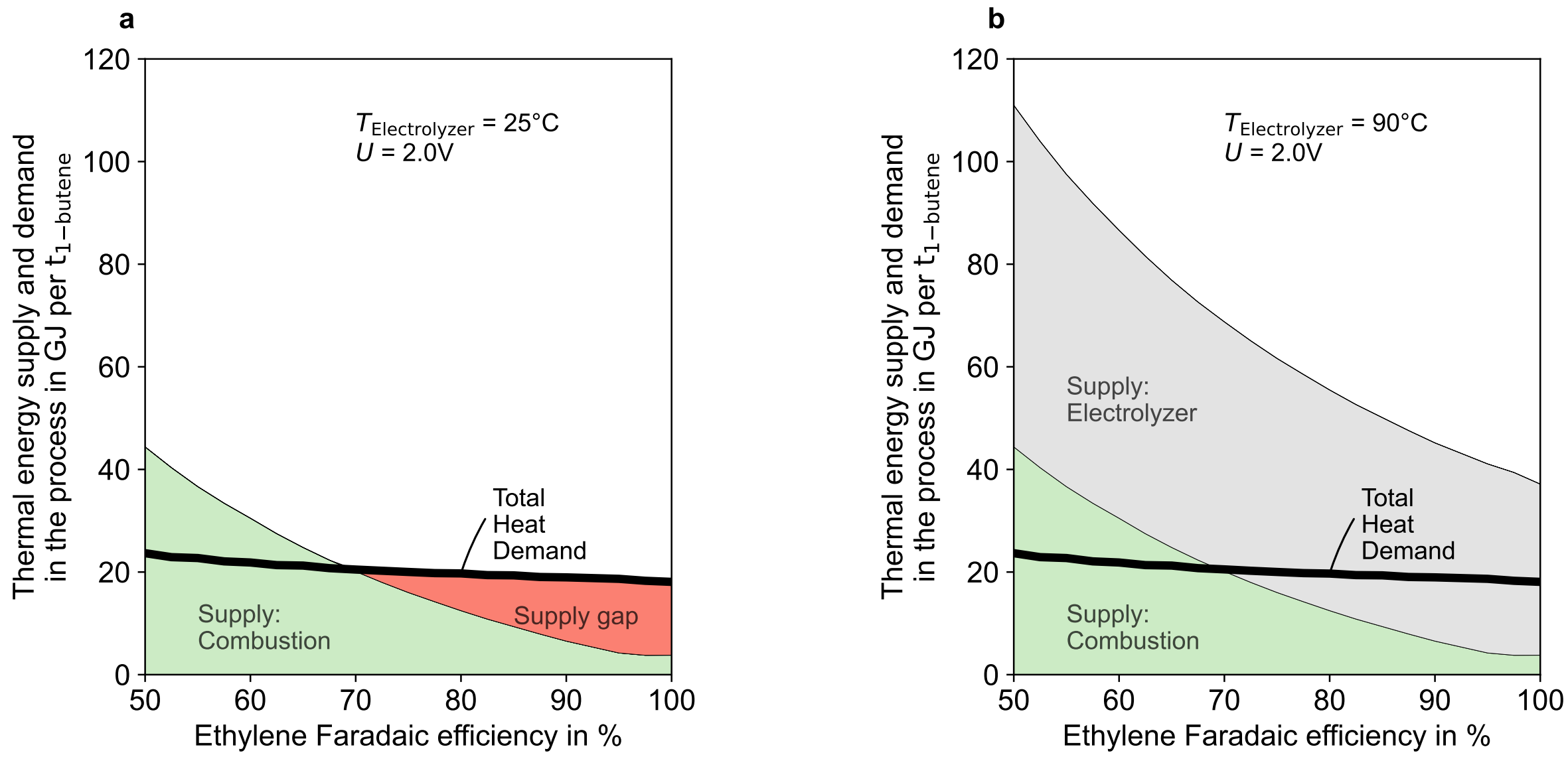

Figure S9: — **Thermal energy supply and demand - Electrolyzer temperature, cell voltage, and Faradaic efficiency.** **a,b** Thermal energy supply and demand for varying Faradaic efficiency at an electrolyzer temperature of 25 °C (a) or 90 °C (b) at a constant cell voltage of 2.0 V.

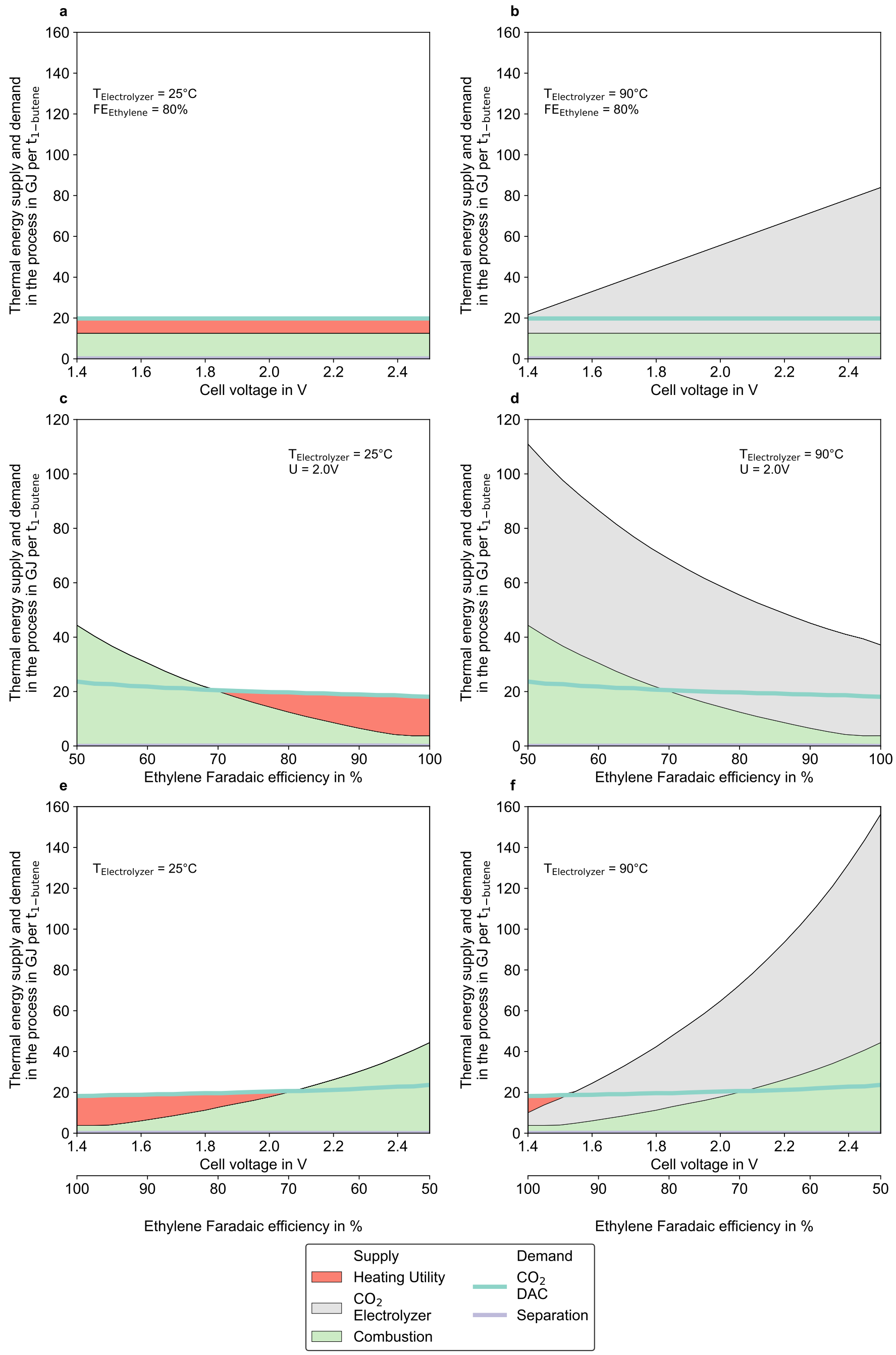

Figure S10: — **Thermal energy supply and demand - Electrolyzer temperature, cell voltage, and Faradaic efficiency.** **a,b** Thermal energy supply and demand for varying cell voltage for an electrolyzer temperature of 25 °C (a) and 90 °C (b) at a constant Faradaic efficiency of 80 %. **c,d** Thermal energy supply and demand for varying Faradaic efficiency for an electrolyzer temperature of 25 °C (c) and 90 °C (d) at a constant cell voltage of 2.0 V. **e,f** Thermal energy supply and demand for varying both Faradaic efficiency and cell voltage jointly for an electrolyzer temperature of 25 °C (e) and 90 °C (f). The thermal energy supply above 90 °C is shown as the hashed area from the combustion by byproducts, waste heat from the electrolyzer, or external heating utilities. The demand is shown as a line for the CO_2 capture of separation units.

S6.4. Main contributors to climate change impacts and cost

The contributions analysis for more electrolyzer temperatures is provided in Figure S11.

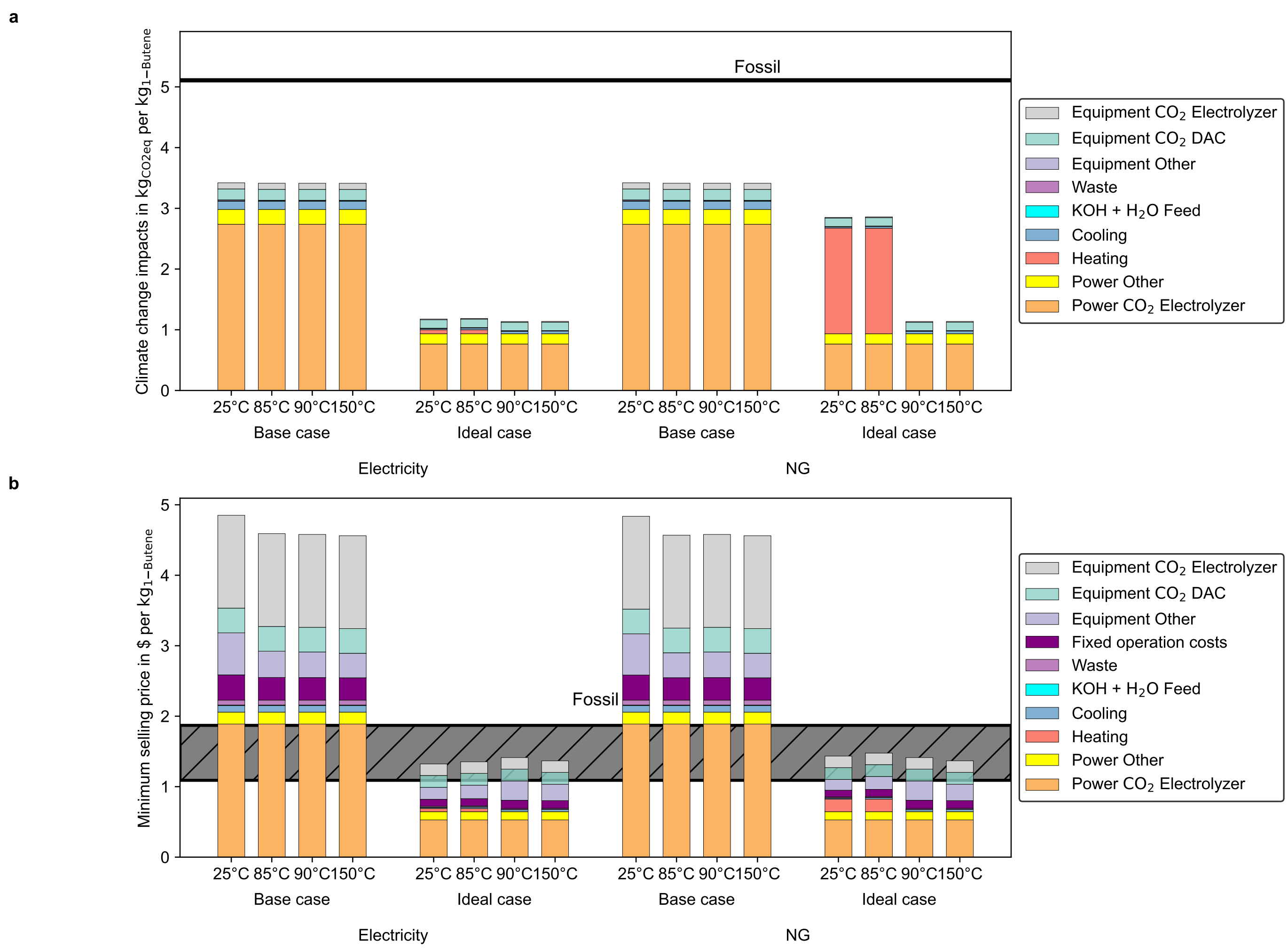

Figure S11: — **Contribution analysis for cost and environmental impacts of 1-butene via CO_2 electroreduction and ethylene dimerization. a,** Contribution to climate change impacts for the production of 1-butene for the base case and the ideal case with different electrolyzer operating temperatures and heating systems. The contribution is split into flowsheet sections. The fossil benchmark is marked in black. **b,** Contribution to the minimum selling price of 1-butene for the base case and the ideal case with different electrolyzer operating temperatures and heating systems. The contribution is split into flowsheet sections. The range of fossil benchmark prices is marked by a gray-hatched band.

The importance of decarbonizing the electricity system is shown in Figure S12. The overall climate change impacts strongly exceed the fossil benchmark when the current average US electricity mix is assumed for the system. The electricity supply dominates the impacts through direct use in the electrolyzer and other process units, as well as indirect use, e.g., for the operation of cooling systems.

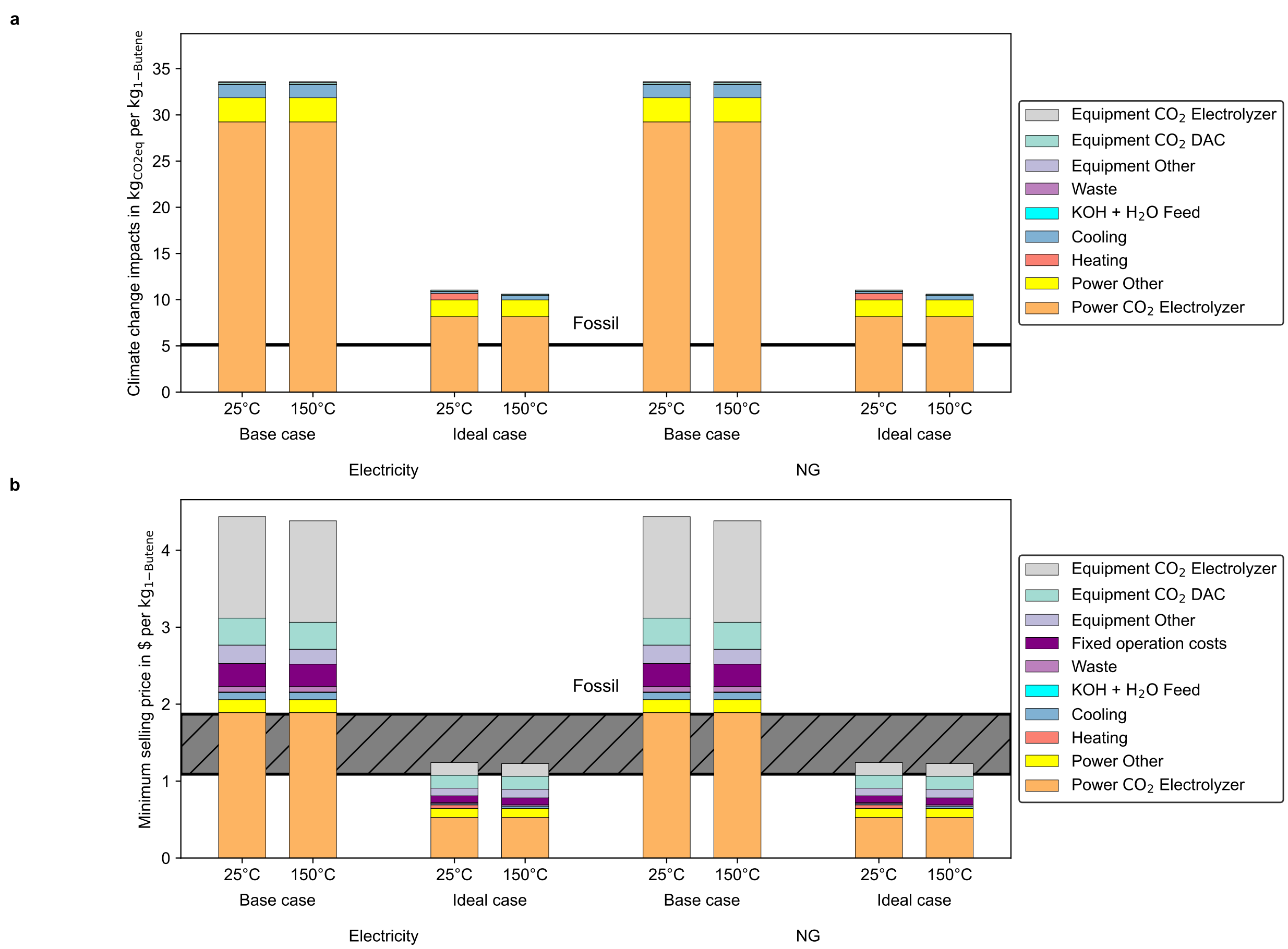

Figure S12: — **Contribution analysis for cost and environmental impacts of 1-butene via CO_2 electroreduction and ethylene dimerization for an US electricity mix. a,** Contribution to climate change impacts for the production of 1-butene for the base case and the ideal case with different electrolyzer operating temperatures and heating systems. The contribution is split into flowsheet sections. The fossil benchmark is marked in black. In contrast to the main manuscript and the results above, a US average electricity mix is assumed rather than electricity generated by wind power. **b,** Contribution to the minimum selling price of 1-butene for the base case and the ideal case with different electrolyzer operating temperatures and heating systems. The contribution is split into flowsheet sections. The range of fossil benchmark prices is marked by a gray-hatched band.

S6.5. CO_2 single pass conversion

A key question is the significance of the single-pass CO_2 conversion, which was held constant in this study for other results. The single-pass CO_2 conversion is a crucial development target for electrolyzers [177]. As described by Moore et al. [3], the energy required for separating electrolyzer product streams can be minor compared to the overall energy demand of the electrolyzer itself. Furthermore, high CO_2 conversion can reduce the Faradaic efficiency for ethylene while increasing the efficiency for other products such as H_2 [3, 178]. However, a minimum CO_2 conversion, often around 10 %, is required depending on the model and cost/LCA assumptions [3, 47, 54, 56, 179]. Beyond this threshold, the energy efficiency of the electrolyzer becomes the primary concern.

The described finding is also observed and confirmed with the model of this work. The climate change impacts for varying CO_2 single pass conversion are given in Figure S13. For a cathodic CO_2 conversion of about 10 %, the climate change impacts remain constant and do not affect the overall performance. The same trend applied to ethylene Faradaic efficiencies from 50 % to 100 %. The benefit of increased single-pass conversion to products is only significant up to a certain point, as noted by Moore et al. [3], Gao et al. [47], Chae et al. [179], Shin et al. [54], and Da Cunha and Resasco [56]. Therefore, maximizing single-pass CO_2 conversion is not the primary target, as separation is expected to require significantly less energy than the electrolyzer operation itself. Consequently, we assume a fixed value for single-pass conversion to products, aligned with the assumptions of Alerte et al. [2].

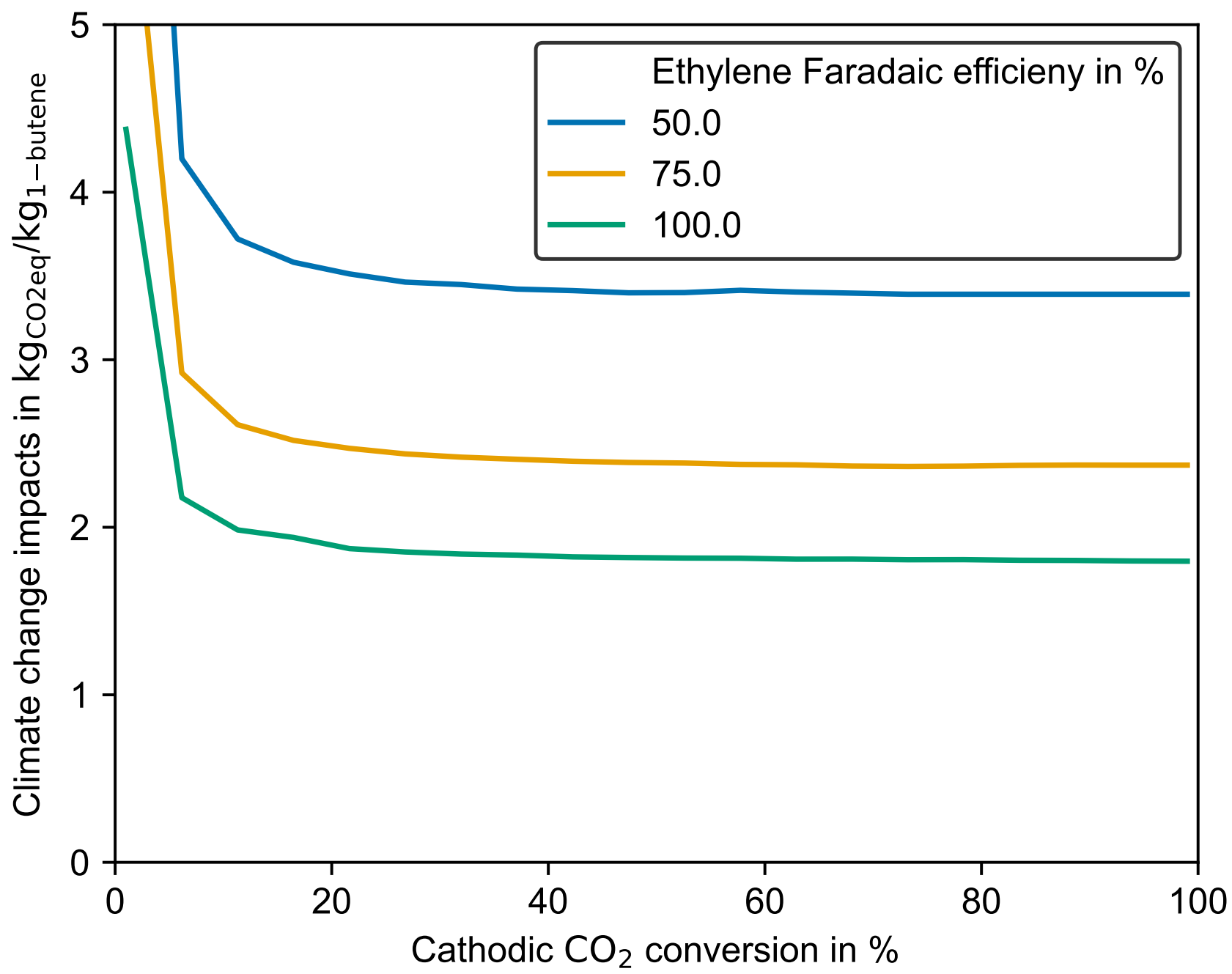

Figure S13: Electrolyzer performance effect on climate change impacts for varying CO_2 single pass conversion (cell voltage 2.5 V). The electrolyzer performance is based on Alerte et al. [2] (see main manuscript).

S6.6. Carbon efficiency

Carbon utilization is a key performance indicator for the overall CO_2 utilization process. The carbon efficiency of the entire process, as shown in Figure S14, generally increases with rising Faradaic efficiency, which is the expected trend. However, the plot also highlights the critical importance of maintaining high Faradaic efficiency, as even slight reductions can lead to significant carbon loss. With multiple separation steps, each contributing approximately 1 % to the carbon loss, these losses can accumulate substantially. Additionally, low CO_2 conversion in the electrolyzer (around 25 %) leads to high recycle streams, further exacerbating carbon loss. This effect must be carefully considered when discussing ideal separation processes and setting targets for CO_2 electroreduction.

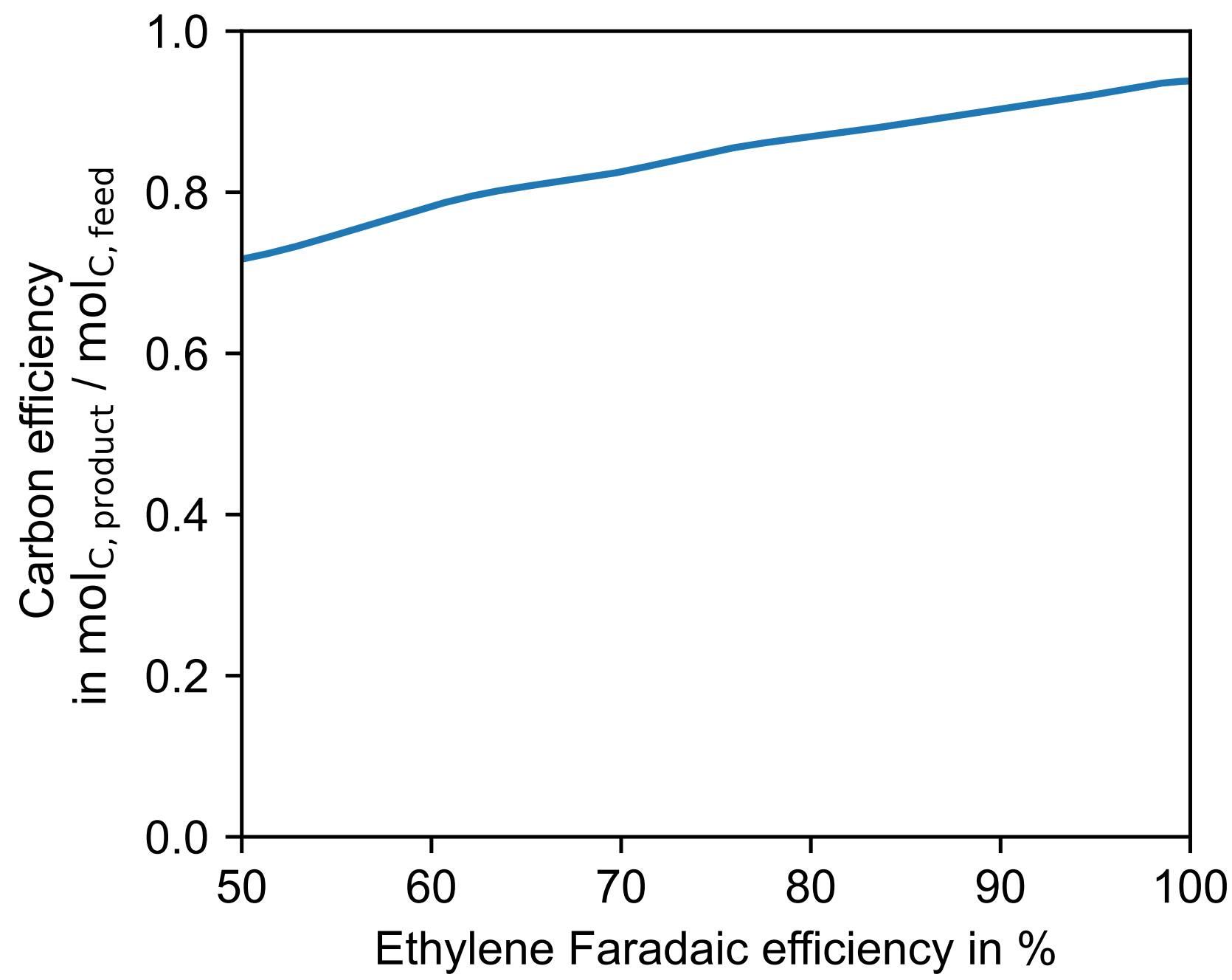

Figure S14: Carbon efficiency of the overall process as mol carbon in 1-butene per mol carbon provided via CO_2 capture over a varying Faradaic efficiency.

S6.7. Byproducts and separation

The separation of byproducts for energy recovery by combustion, e.g., the liquid cathode separation, adds energy demand. To analyze whether this separation is energetically favorable, we compare the required energy demand for separation to the heat of combustion.

First, we compare the computed energy demands for the separation: The separation of C4 (butene, butane) from the gaseous mix of CO_2, carbon monoxide, and H_2 requires much less energy ($\approx 18\,\frac{\mathrm{kJ}}{\mathrm{mol_{CO+H2}}}$ for the base case) compared to the water-ethanol separation ($\approx 250\,\frac{\mathrm{kJ}}{\mathrm{mol_{EtOH}}}$), which we both consider in our process (water is considered due to the humidification of the CO_2 feedstock). Separation of CO_2 from carbon monoxide and H_2 is not considered in our study, as it is unnecessary. The energy demand for the ethanol-water separation is within the range reported in the literature, considering different degrees of purification, initial concentration, and process designs [180, 181], and still below the enthalpy of combustion of ethanol (Table S15), suggesting that its recovery is energetically favorable.

Still, from an energy recovery perspective, carbon monoxide and H_2 are more promising targets than ethanol, as the gained enthalpy of combustion per electron transferred in the CO_2 electrolyzer is higher compared to ethanol (Table S15). This effect is also reflected in the higher standard potential. As the same cell potential is applied for all (by)product-forming reactions, more energy is chemically stored in carbon monoxide or H_2, thus available for combustion, and less energy is lost to waste heat compared to ethanol.

A summary of options for combustion, disposal, and recovery for the electrolyzer byproducts is given in Table S17 and Figure S15.

Table S15: Standard potential and enthalpies of combustion for the (by)products considered in this study.

(By)product	Electrons transferred $\left[\frac{\mathrm{mol_{e^-}}}{\mathrm{mol}}\right]$	Standard potential [V] [41]	Standard enthalpy of combustion $\left[\frac{\mathrm{kJ}}{\mathrm{mol}}\right]$ [41]	Standard enthalpy of combustion $\left[\frac{\mathrm{kJ}}{\mathrm{mol_{e^-}}}\right]$
Ethylene	12	-1.150	-1411	-118
H_2	2	-1.229	-286	-143
Carbon monoxide	2	-1.333	-283	-142
Ethanol	12	-1.145	-1367	-114
Acetate (assuming acetic acid)	8	-1.131	-875	-109
n-Propanol	18	-1.129	-2021	-112

Table S17: Downstream process options and energy considerations corresponding to Figure S15. The chosen options are marked with a yellow circle.

Number	Option	Implications
(1)	Combustion of gaseous byproducts	Thermal recovery of heat of combustion, very little separation energy demand
2	Disposal of gaseous byproduct streams without separation	High loss of CO_2 feedstock and carbon due to byproduct formation and incomplete CO_2 conversion, no energy demand for separation
3	Separation of CO_2 and disposal of byproducts	Energy demand for separation, CO_2 recovery, loss of byproducts
4	Separation of CO_2 and purification of byproducts	High separation energy demand to reach purities of 5+ component mixture, full recovery of carbon
5	Disposal of liquid byproducts	Loss of byproducts, no energy demand
6	Liquid separation for the purification of byproducts	Full carbon recovery at high energy demands as multiple azeotropes involved (e.g., water-ethanol, water-propanol)
(7)	Concentration of byproducts with remaining water content and combustion of mixture	Low energy demand for separation, as no full purification over azeotropes, high carbon recovery rate, energy gain in combustion

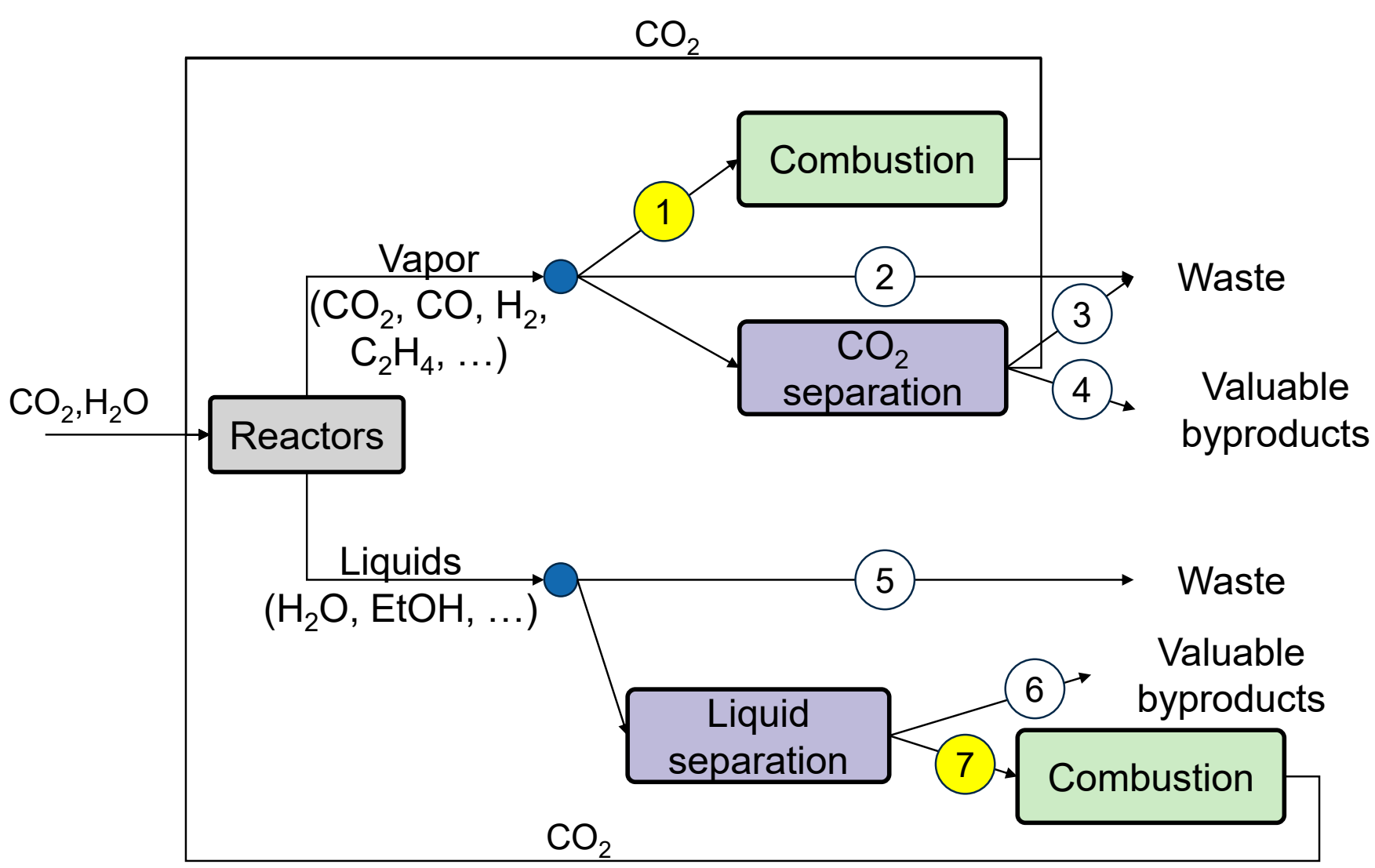

Figure S15: Downstream separation and recycle options for the reaction byproducts in the 1-butene process. The yellow options are considered in this work, and all options are discussed in Table S17.

S6.8. Electrolyzer operation under pressure

In industrial applications, electrolyzers might be operated under pressure [182]. While a detailed analysis of the pressure effect is outside the scope of this study, we estimate and discuss potential implications here. Increasing the electrolyzer operating pressure would affect both (1) the overall process with compression energy demand, cost, and climate change impacts, as well as (2) the electrolyzer operating on a catalysis level.

At a process level (1), the compression energy demand is minimal if one assumes to compress the whole CO_2 feedstock to 50 bar (assuming an ideal gas, c_p of CO_2 [183], a carbon efficiency of 80 % (see Section S6.6), and an isentropic efficiency of 85 % [184], neglecting intermediate cooling steps). The calculated compression energy demand of 1.6 $\frac{\mathrm{GJ}}{\mathrm{t_{1-butene}}}$ (Equations S43-S46) is around 1 % or less of the overall process electricity demand (96 $\frac{\mathrm{GJ}}{\mathrm{t_{1-butene}}}$ to 274 $\frac{\mathrm{GJ}}{\mathrm{t_{1-butene}}}$: ideal case vs. base case, see Section S6.2). Furthermore, gas separation (butene distillation and pressure swing adsorption) would benefit from increased pressure, reducing the need for compression after the reaction steps.

$$k_{\mathrm{CO2}} = \frac{c_\mathrm{p}}{c_\mathrm{v}} = \frac{c_\mathrm{p}}{c_\mathrm{p} - R} \approx \frac{37.2\,\frac{\mathrm{J}}{\mathrm{mol\,K}}}{37.2\,\frac{\mathrm{J}}{\mathrm{mol\,K}} - 8.314\,\frac{\mathrm{J}}{\mathrm{mol\,K}}} = 1.288 \tag{S43}$$

$$W_{\mathrm{comprssion}} = \frac{n_{\mathrm{CO2}}}{\eta_{\mathrm{carbon}} \cdot n_{1-\mathrm{butene}} \cdot M_{1-\mathrm{Butene}}} \cdot \frac{1}{\eta_{\mathrm{isentropic}}} \cdot \frac{k}{k-1} \cdot RT_0 \left[\left(\frac{p_2}{p_1} \right)^{\frac{k-1}{k}} - 1 \right] \tag{S44}$$

$$= \frac{4}{0.8 \cdot 1 \cdot 56\,\frac{\mathrm{g}}{\mathrm{mol}}} \cdot \frac{1}{0.85} \cdot \frac{1.288}{1.288 - 1} \cdot 8.314\,\frac{\mathrm{J}}{\mathrm{mol\,K}} \cdot 298.15\,\mathrm{K} \cdot \left[\left(\frac{50\,\mathrm{bar}}{1\,\mathrm{bar}} \right)^{\frac{1.288-1}{1.288}} - 1 \right] \tag{S45}$$

$$= 1628\,\frac{\mathrm{J}}{\mathrm{g_{1-butene}}} = 1.628\,\frac{\mathrm{GJ}}{\mathrm{t_{1-butene}}} \tag{S46}$$

On the electrolyzer level (2), the CO_2 concentration would increase, potentially steering the selectivity to formate [185, 186]. Additional effects might be an increased current density [186] or higher FEs for products such as carbon monoxide [182], which in turn might reduce the ethylene FE. Here, catalyst development might prevent a large shift in FEs. Electrolyzer operation at higher temperatures weakens surface binding, whereas increased pressure can strengthen it. Additionally, higher pressures and increased CO_2 concentrations can prevent flooding of the electrolyzer [17]. Furthermore, the membrane stability needs to be investigated under increased pressure.

S6.9. Sensitivity analysis on ethylene dimerization

In our system, the conversion of ethylene to butene depends on the efficiency of the dimerization catalyst (such as H:RuNi, as reported by Lee et al. [187]). The ethylene concentration, in turn, is determined by the performance of the CO_2 electrolyzer. Therefore, the formation rate of butene isomers varies depending on the ethylene concentration generated from the CO_2 electrolyzer. Experimental data on catalyst sensitivity are not yet available. Hence, we conducted a sensitivity analysis of the system's performance on the ethylene conversion to 1-butene.

The analysis reveals a strong dependence of total process electricity demand on the ethylene conversion, particularly for lower ethylene FE (Figure S16). At lower ethylene FEs, reduced ethylene conversion to 1-butene leads to greater loss via the combustion recycle loop. However, high ethylene conversions were experimentally achieved by Lee et al. [187] and are therefore considered in this work. The selectivity of ethylene toward 1-butene and n-butane is kept constant in the analysis. Nevertheless, further development and experimental validation of dimerization kinetics under varying feed conditions are needed.

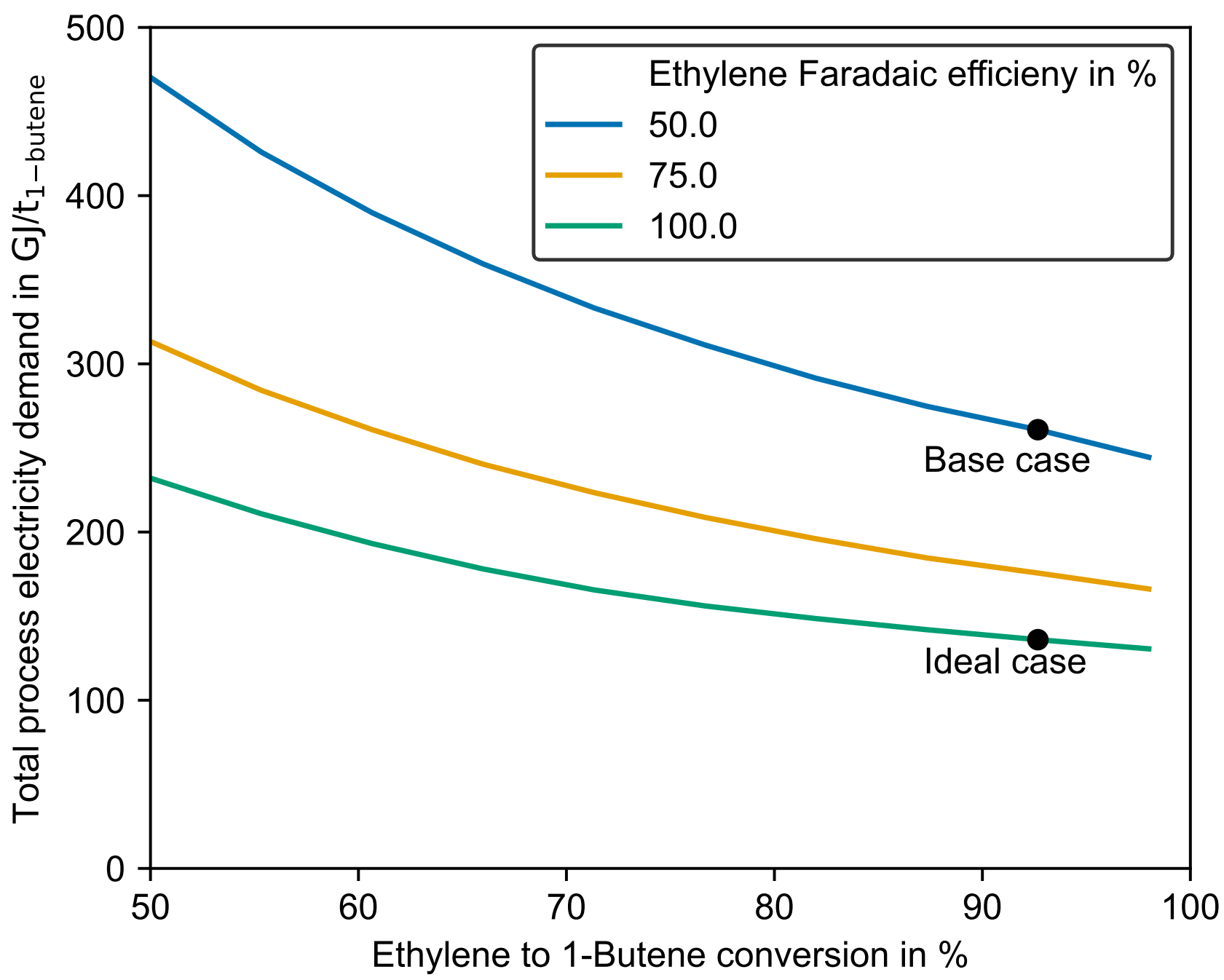

Figure S16: Sensitivity analysis: dependence of the overall process electricity demand on the ethylene conversion to 1-butene. The ethylene conversion to butane is scaled accordingly. Electrolyzer performance: 2.4 V, 25 °C, all other parameters as in Table S2.

S6.10. Flowsheet choices

The flowsheet configuration for the process of CO_2 utilization via electroreduction and dimerization to 1-butene has been fixed for this study by defined unit operations and connections. However, some choices and separations are optional. We investigated two optional separations: an intermediate ethylene separation between the electrolyzer and the dimerization reactor and a separation of the liquid cathode stream. We provide the process flowsheet from the main manuscript here for the sake of completeness and include optional choices in the flowsheet (Figure S17).

Ethylene can be purified before the dimerization to 1-butene. However, Lee et al. [188] demonstrated a direct dimerization without purification. In this work, ethylene is optionally separated from the other components before being fed to the dimerization step. Without further detail, an entropic efficiency separation with 7 % is considered (same as for anode separation), which consists of two separation steps (one for higher boilers (mostly gases like CO_2, H_2, carbon monoxide) and lower boilers (mostly liquid reaction products (H_2O, ethanol, 1-propanol, acetate)).

Secondly, the cathode liquid stream can be discarded or purified to recover byproducts lost to the liquid. If considered, cathode liquids are separated using a distillation column to reduce carbon loss. A full separation of all H_2O is neglected as H_2O and 1-propanol form an azeotrope, which increases separation cost.

Neither optional separation step significantly influenced overall process performance, as their respective energy demands are significantly lower than those of other process units. Therefore, intermediate ethylene purification is neglected, following the work of Lee et al. [188], and cathode liquid separation is considered to increase carbon efficiency. Furthermore, the cathode liquid separation distillation column can be driven by waste heat from other unit operations.

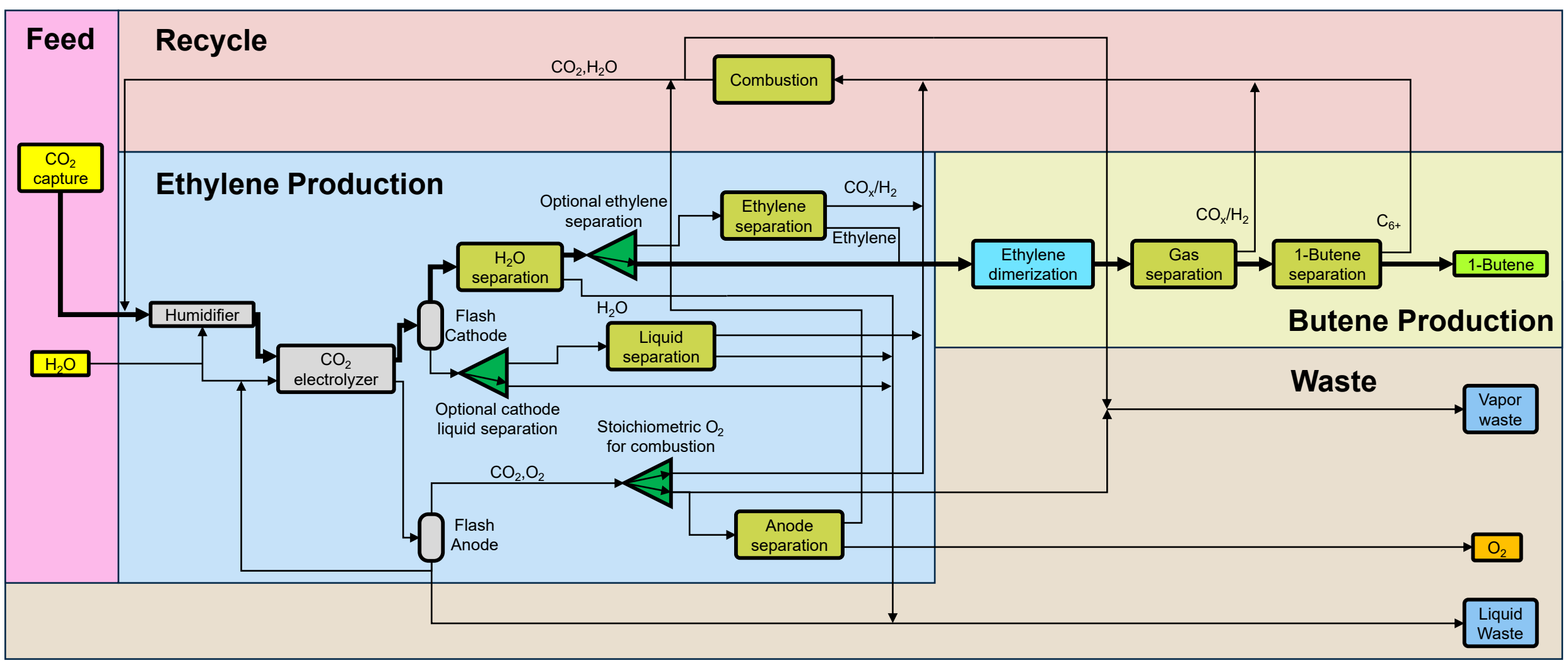

Figure S17: Process flow diagram for the CO_2 utilization via electroreduction and dimerization to 1-butene. The process comprises CO_2 capture (adsorbent-based direct air capture) and water feed, CO_2 electroreduction to ethylene (AEM electrolyzer) and downstream separation (entropic efficiency separation and distillation), ethylene dimerization to 1-butene with 1-butene separation (cryogenic distillation), and CO_2 recycle including combustion of byproducts.

S6.11. Electrolyzer cell lifetime

Operating the electrolyzer at higher temperatures introduces trade-offs, including accelerated membrane degradation, increased gas and carbonate crossover, and rapid catalyst destabilization [189–191]. However, these trade-offs remain unquantified because the overarching effects of elevated operating temperatures have not yet been fully investigated.

Figures S18 and S19 quantify the trade-off between elevated electrolyzer temperature and cell degradation, balancing enhanced performance against the penalty of a reduced cell lifetime. While a shorter lifetime increases both climate change impacts and the minimum selling price of 1-butene, this penalty is outweighed by the performance gains of higher operating temperatures, provided the lifetime remains above 0.5 yr to 5 yr (depending on the metric and assumptions). This threshold falls within or below the lifetime ranges typically proposed in the literature [192–195].

For Figures S18 and S19, cell stacks are assumed to account for 50 % of the initial electrolyzer investment cost. This cost recurs upon cell replacement as lifetime decreases. This 50 % contribution represents an upper bound that implicitly accounts for auxiliary expenses, such as plant downtime during replacements. In comparison, literature values typically range from 5 % to 45 % [196–198]. Similarly, the life cycle assessment assumes that the cell life cycle inventory [60] is required more frequently as lifetime decreases. Electrolyzer performance parameters are adopted from Figure 4 of the main manuscript.

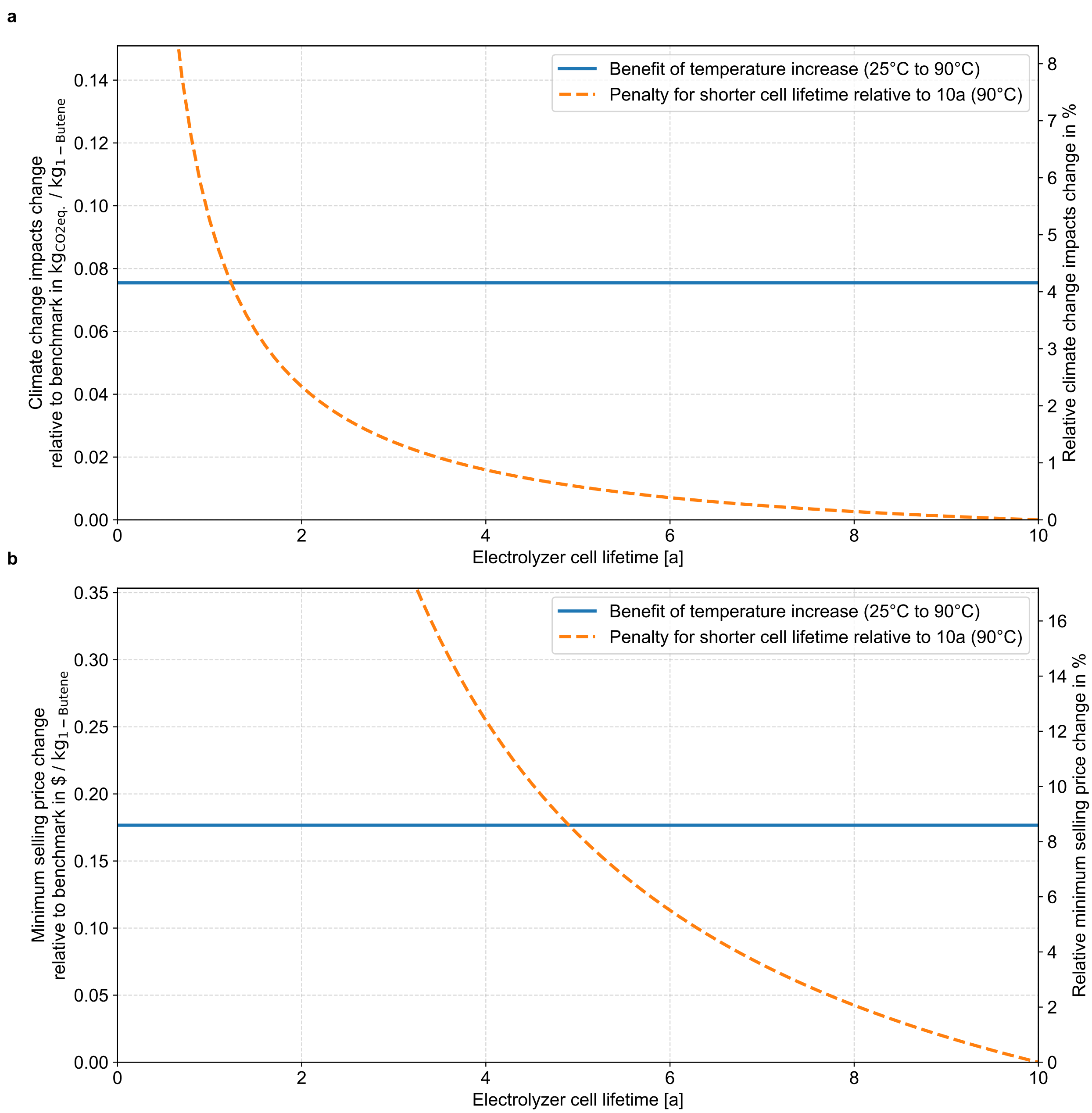

Figure S18: Sensitivity analysis: Increase in climate change impacts (a) and minimum selling price (b) over the electrolyzer cell lifetime. The benefit of increased electrolyzer temperature is given as a horizontal reference. We compare the electrolyzer performance at a constant cell voltage of 2.5 V and Faradaic efficiency of 100 % (corresponding to the very right points in Figure 4b/d in the main manuscript). External heat is provided via a heat pump.

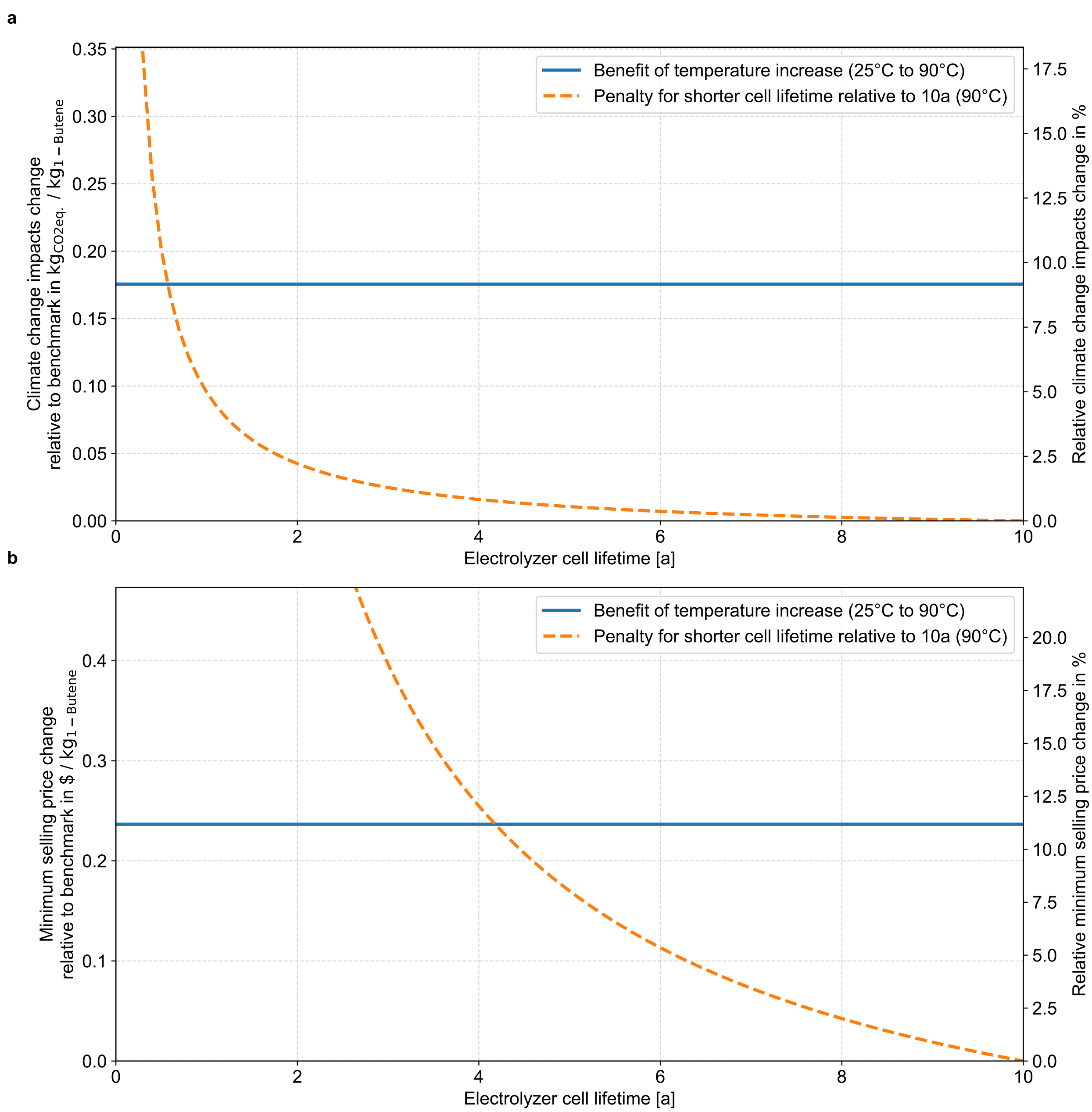

Figure S19: Sensitivity analysis: Increase in climate change impacts (A) and minimum selling price (B) over the electrolyzer cell lifetime. The benefit of increased electrolyzer temperature is given as a horizontal reference. We compare the electrolyzer performance at a constant cell voltage of 2.5 V and Faradaic efficiency of 100 % (corresponding to the very right points in Figure 4b/d in the main manuscript). External heat is provided via an electric boiler.

S6.12. Thermal energy saving potential estimation for other CO_2 electroreduction products

We assess the potential of thermal energy integration for other products aside from ethylene/ 1-butene via a simplified energy balance analysis. We consider a simplified process (Figure S20). The only energy demands considered are the thermal and electrical energy demand for CO_2 capture as well as the electrical energy demand of the electrolyzer. The electrolyzer converts the electrical energy input into chemical energy in product and byproduct, as well as ohmic waste heat.

The electrolyzer is assumed to operate at elevated temperature to utilize the ohmic waste heat, and the temperature is sufficiently high to supply heat directly to the CO_2 capture unit. For the calculation of the byproduct energy, the same thermodynamic minimum voltage is assumed for both product and byproducts. The byproducts are assumed to be combusted at 100 % efficiency to recover their chemical energy if needed.

The process is idealized, and carbon losses in recycle streams due to incomplete CO_2 conversion or incomplete separation are neglected. Furthermore, the energy demand of downstream separation or other inefficiencies is neglected.

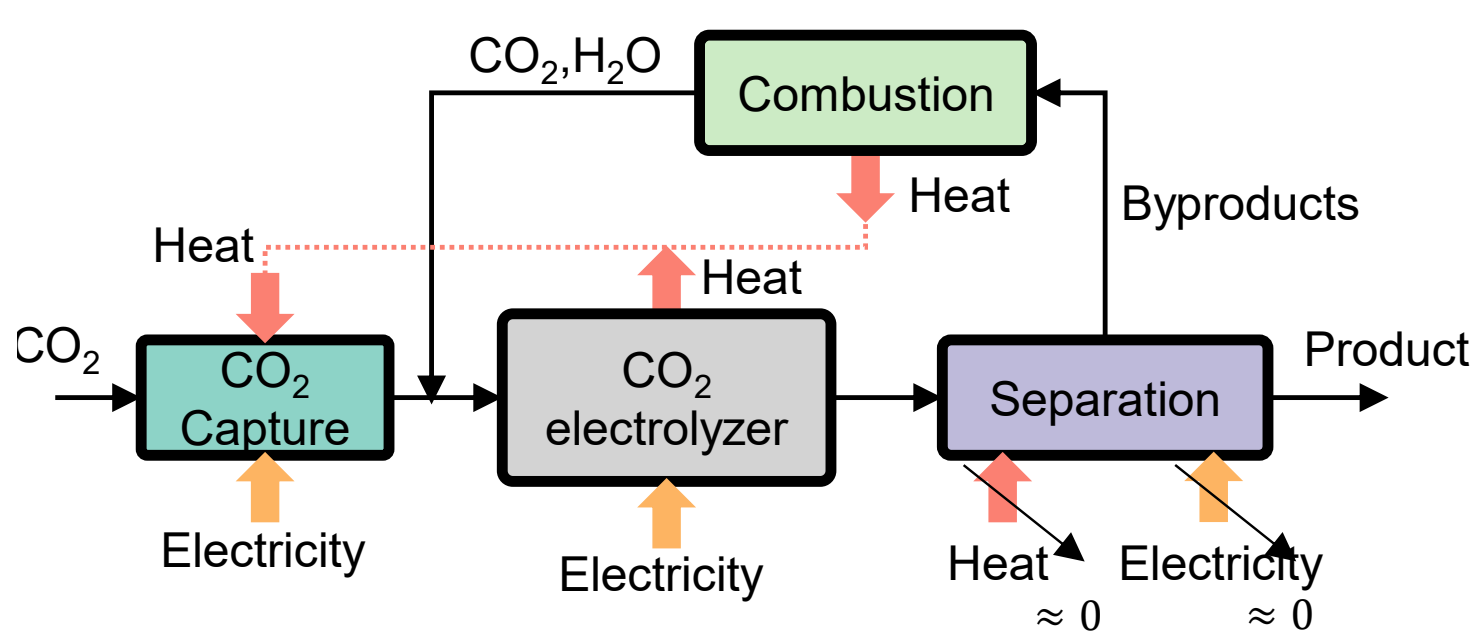

Figure S20: Process flow diagram for a simplified electrochemical process converting CO_2 to products. The downstream separation is neglected, while CO_2 capture, electrolyzer, and the byproduct combustion are considered.

The energy streams in this simplified process are calculated via Equations S47 to S57.

Total electrolyzer energy input

$$w_{\text{electrolyzer,total}} = \frac{UFz_{\text{product},i}}{\text{FE}_{\text{product},i} M_{\text{product},i}} \tag{S47}$$

Electrolyzer energy chemically converted to product molecule

$$w_{\text{elecolyzer,product}} = \frac{U_{\text{min},i} F z_{\text{product},i}}{M_{\text{product},i}} \tag{S48}$$

Electrolyzer energy chemically converted to byproduct molecules

$$w_{\text{elecolyzer,byproducts}} = \frac{Fz_{\text{product},i}}{\text{FE}_{\text{product},i} M_{\text{product},i}} \sum_{\text{byproducts},j} (U_{\text{min},j} \text{FE}_j) \tag{S49}$$

$$\approx \frac{Fz_{\text{product},i}}{\text{FE}_{\text{product},i} M_{\text{product},i}} U_{\text{min},i} (1 - \text{FE}_{\text{product},i}) \tag{S50}$$

Electrolyzer energy lost to waste heat

$$w_{\text{elecolyzer,waste heat}} = \frac{Fz_{\text{product},i}}{\text{FE}_{\text{product},i} M_{\text{product},i}} \left(U - U_{\text{min},i} \text{FE}_{\text{product},i} - \sum_{\text{byproducts},j} (U_{\text{min},j} \text{FE}_j) \right) \tag{S51}$$

$$\approx \frac{Fz_{\text{product},i}}{\text{FE}_{\text{product},i} M_{\text{product},i}} \left(U - U_{\text{min},i} \text{FE}_{\text{product},i} - U_{\text{min},i} \left(1 - \text{FE}_{\text{product},i}\right)\right) \tag{S52}$$

$$= \frac{Fz_{\text{product},i}}{\text{FE}_{\text{product},i} M_{\text{product},i}} (U - U_{\text{min},i}) \tag{S53}$$

The CO_2 capture energy demand is scaled to the product muss via

$$w_{\text{CO2 capture}} = \frac{W}{m_{\text{CO2}}} \cdot \frac{M_{\text{CO2}} n_{\text{product},i}}{M_{\text{product},i}} \tag{S54}$$

Depending on the heating scenario (electric boiler or heat pump), the thermal energy demand of the CO_2 capture unit is converted to an electricity demand

$$w_{\text{CO2 capture,elec,heating}} = \frac{w_{\text{CO2 capture,heating}}}{\text{COP}_{\text{heating}}} \tag{S55}$$

For an electric boiler, the COP/efficiency is set to 99 % [107](see Section S1.6). For a heat pump, Table S21 is used.

The total absolute energy demand (summing thermal energy and electrical energy) sums to:

$$\begin{aligned} w_{\text{total}} &= w_{\text{CO2 capture}} + w_{\text{electrolyzer,total}} + \cancelto{0}{w_{\text{downstream}}} & \text{(S56)} \\ &= w_{\text{CO2 capture,elec}} + w_{\text{CO2 capture,therm}} + w_{\text{elecolyzer,product}} + w_{\text{elecolyzer,byproducts}} & \text{(S57)} \\ &\quad + w_{\text{elecolyzer,waste heat}} \end{aligned}$$

The electrical energy demand sums to:

$$\begin{aligned} w_{\text{total,elec}} = & w_{\text{CO2 capture,elec}} + w_{\text{CO2 capture,elec,heating}} + w_{\text{elecolyzer,product}} \\ & + w_{\text{elecolyzer,byproducts}} + w_{\text{elecolyzer,waste heat}} \end{aligned} \tag{S58}$$

The variables describe the total cell potential U, the minimum cell potential for a given product $U_{\text{min},i}$, Faraday constant F, the number of electrons transferred for the synthesis of the product from CO_2 $z_{\text{product},i}$, the Faradaic efficiency $\text{FE}_{\text{product},i}$, the molar mass $M_{\text{product},i}$, the number of CO_2 molecules per product molecule $n_{\text{product},i}$, coefficient of performance COP, and energy demands w.

The parameters for individual CO_2 electroreduction products are given in Table S19. As the minimum cell potential $U_{\text{min},i}$ is similar for the individual products, we assume a constant total cell potential $U = 2.5\,\text{V}$ for simplicity. We consider sorbent-based direct air capture and solvent-based point-source capture for CO_2 supply (Table S21).

Table S19: Electrochemical parameters for various CO_2 reduction products.

Product	Formula	Number of CO_2 molecules per product molecule	Number of electrons transferred	Minimum cell potential in V [2, 9, 12]	Range of reported achieved Faradaic effiency in %
Ethylene	C_2H_4	2	12	1.15	65-85 [11, 14, 15, 199]
Carbon monoxide	CO	1	2	1.33	80-100 [11, 199]
Formic acid	HCOOH	1	2	1.48	80-100 [199]
Methanol	CH_3OH	1	6	1.21	40-85 [199]
Acetate	CH_3COOH	2	8	1.12	40-85 [199, 200]
Ethanol	C_2H_5OH	2	12	1.15	20-85 [11, 199, 200]
n-Propanol	C_3H_7OH	3	18	1.14	10-60 [199, 200]

Table S21: CO_2 Capture Alternatives.

Technology	Electrical energy demand in $\frac{MJ}{kg_{CO2}}$	Thermal energy demand in $\frac{MJ}{kg_{CO2}}$	Required temperature level	Heat pump COP	Reference
Sorbent-based direct air capture	1.8	5.4	below 100 °C	2.51	[97]
MEA solvent point-source capture	0.0421	3.98	121 °C reboiler temperature	2.1	[201–203]

In most cases, the electrolyzer waste heat can supply the whole thermal energy demand for the CO_2 capture (Figures S21 to S26). Only when Faradaic efficiency is high (no energy loss to byproducts) is the electrolyzer waste heat sufficient to supply thermal energy for CO_2 capture. Then, in most cases, both the electrolyzer waste heat and the thermal utilization of the byproduct are used to meet the thermal energy demand.

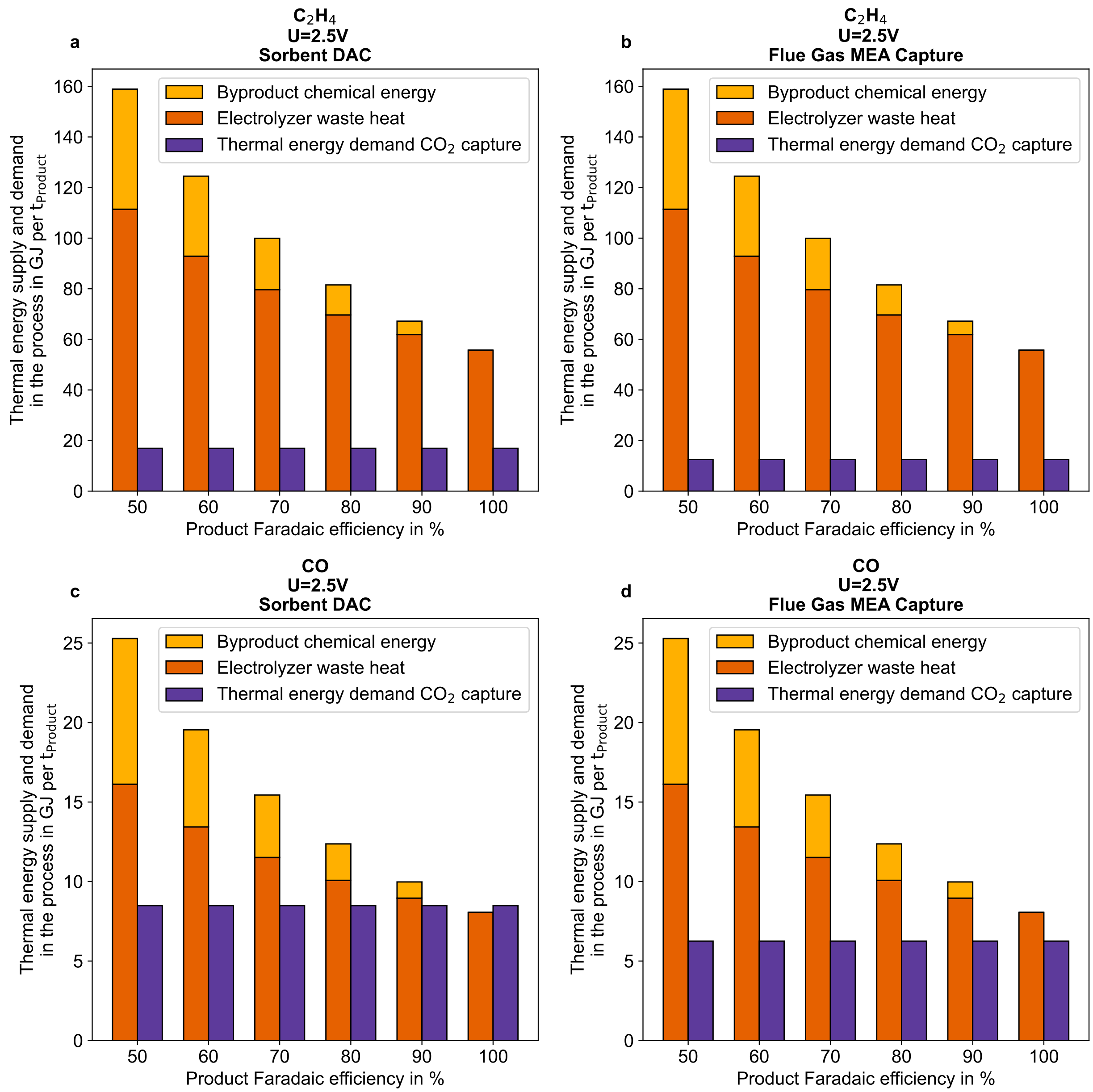

Figure S21: — **Thermal energy supply and demand - Ethylene, carbon monoxide a,b** Thermal energy supply and demand for ethylene as target product over the ethylene Faradaic efficiency for (a) direct air capture CO_2 supply and (b) point-source CO_2 capture. **c,d** Thermal energy supply and demand for carbon monoxide as target product over the carbon monoxide Faradaic efficiency for (c) direct air capture CO_2 supply and (d) point-source CO_2 capture.

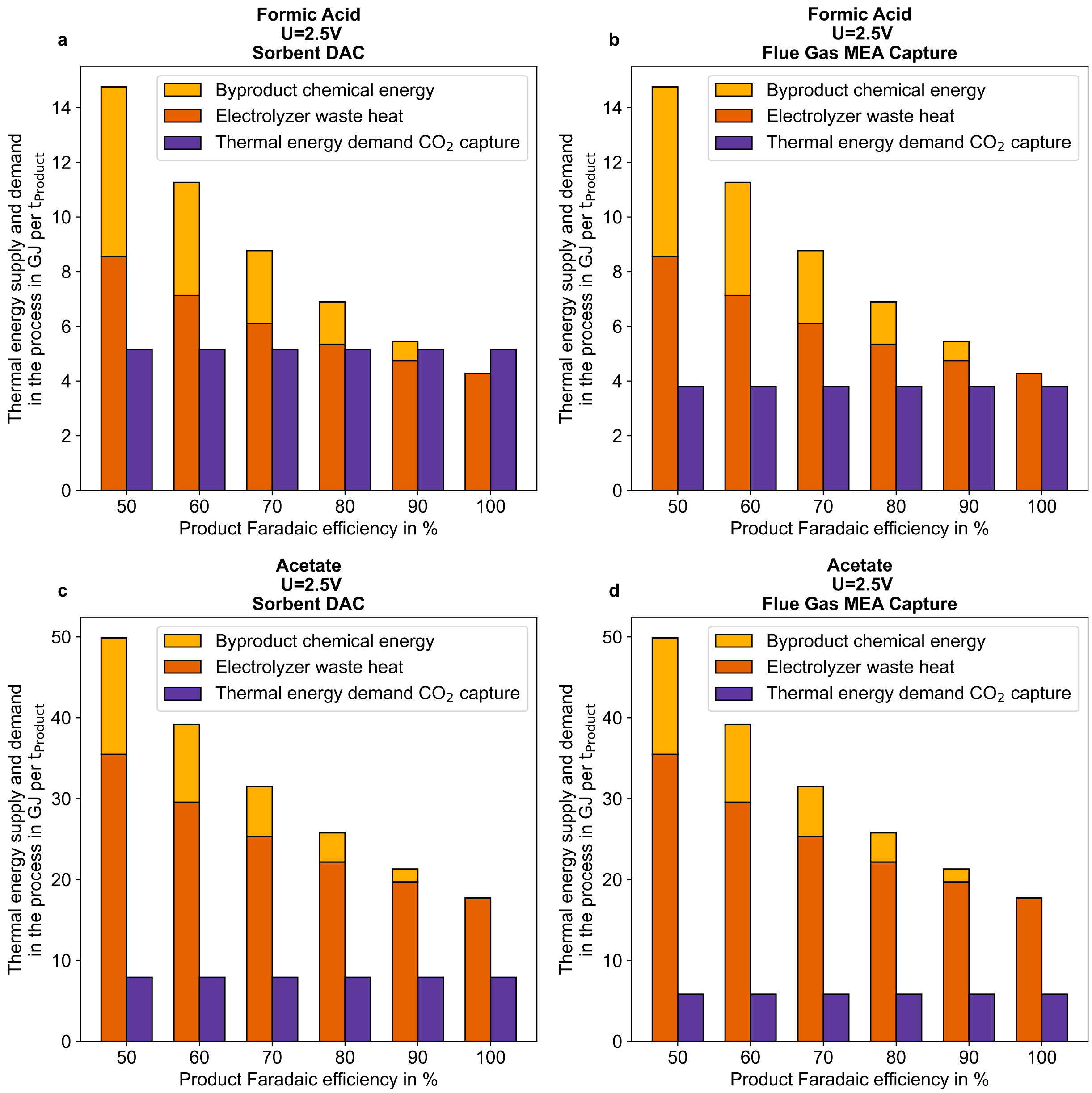

Figure S22: — **Thermal energy supply and demand - Formic acid, acetate a,b** Thermal energy supply and demand for formic acid as target product over the formic acid Faradaic efficiency for (a) direct air capture CO_2 supply and (b) point-source CO_2 capture. **c,d** Thermal energy supply and demand for acetate as target product over the acetate Faradaic efficiency for (c) direct air capture CO_2 supply and (d) point-source CO_2 capture.

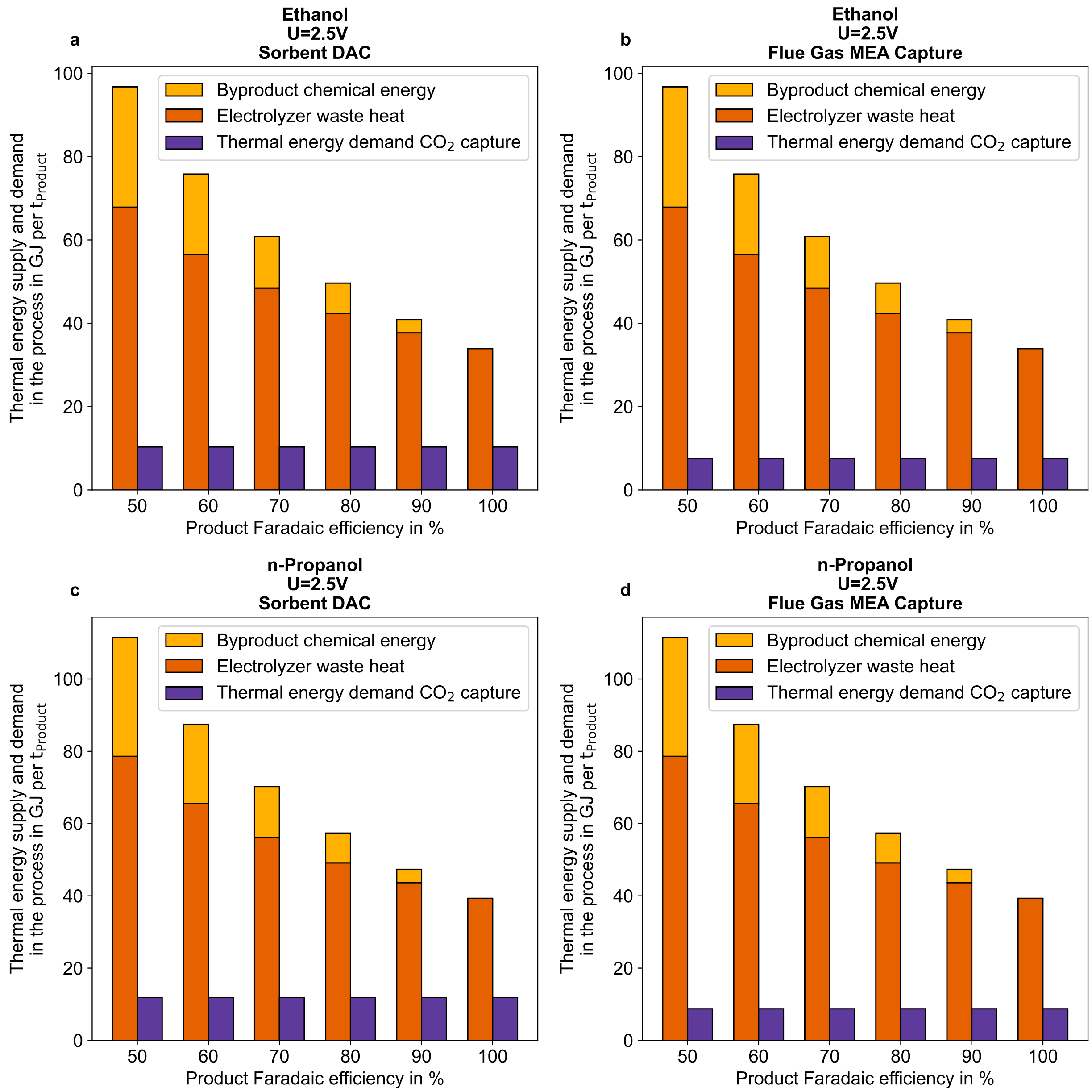

Figure S23: — **Thermal energy supply and demand - Ethanol, n-propanol a,b** Thermal energy supply and demand for ethanol as target product over the ethanol Faradaic efficiency for (a) direct air capture CO_2 supply and (b) point-source CO_2 capture. **c,d** Thermal energy supply and demand for n-propanol as target product over the n-propanol Faradaic efficiency for (c) direct air capture CO_2 supply and (d) point-source CO_2 capture.

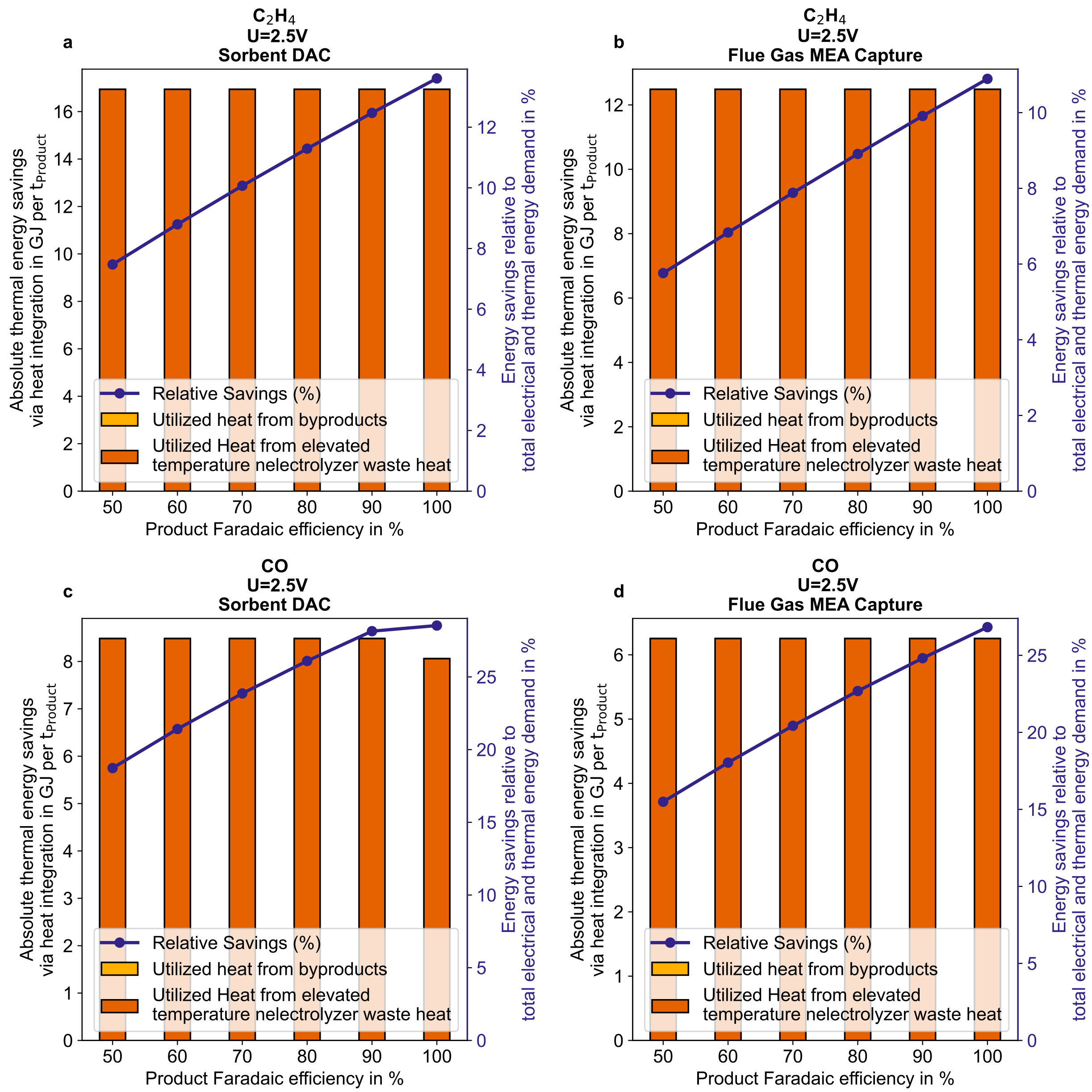

Figure S24: — **Potential thermal energy savings - Ethylene, carbon monoxide a,b** Potential thermal energy savings for ethylene as target product over the ethylene Faradaic efficiency for (a) direct air capture CO_2 supply and (b) point-source CO_2 capture. **c,d** Potential thermal energy savings for carbon monoxide as target product over the carbon monoxide Faradaic efficiency for (c) direct air capture CO_2 supply and (d) point-source CO_2 capture.

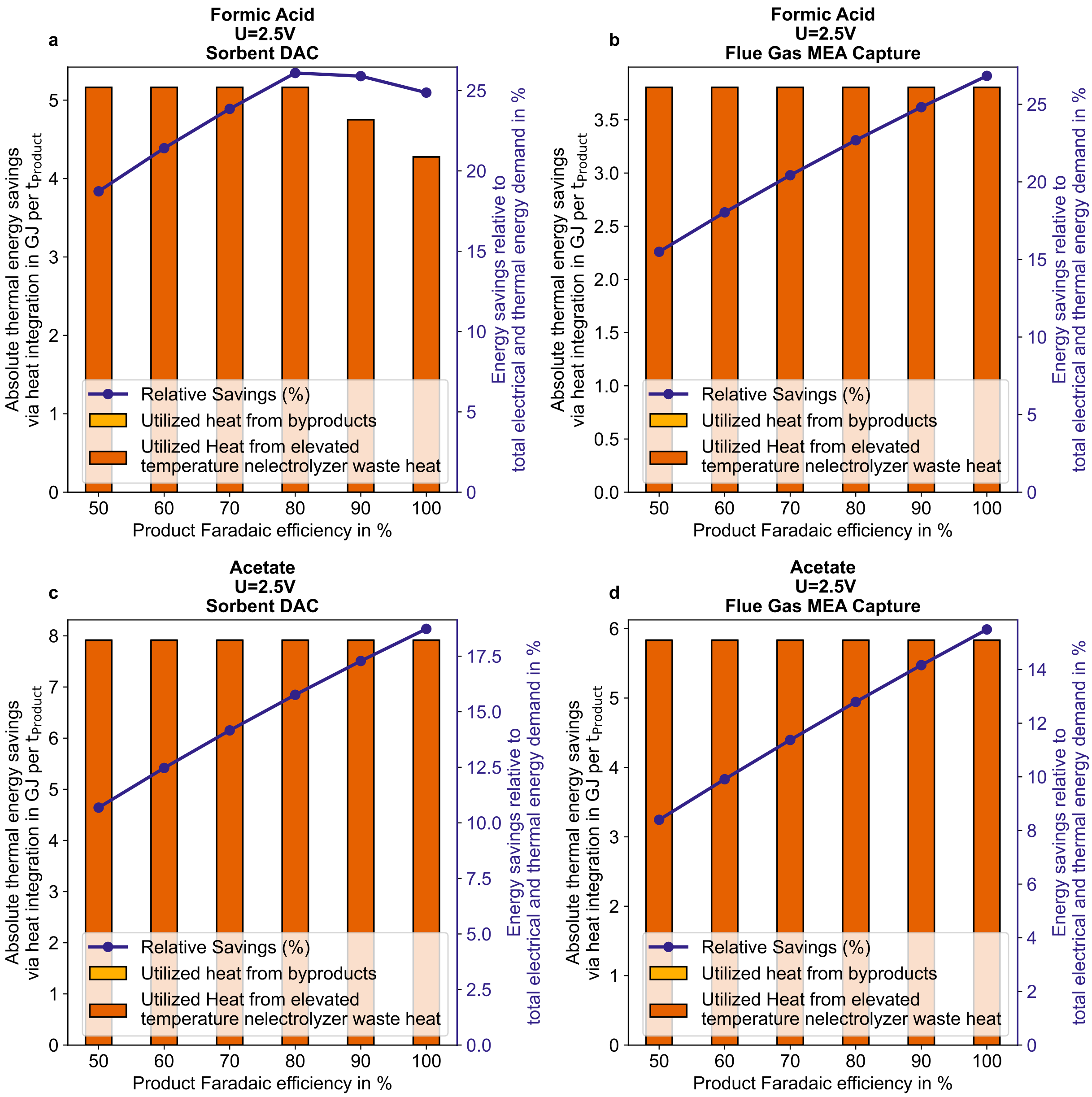

Figure S25: — **Potential thermal energy savings - Formic acid, acetate a,b** Potential thermal energy savings for formic acid as target product over the formic acid Faradaic efficiency for (a) direct air capture CO_2 supply and (b) point-source CO_2 capture. **c,d** Potential thermal energy savings for acetate as target product over the acetate Faradaic efficiency for (c) direct air capture CO_2 supply and (d) point-source CO_2 capture.

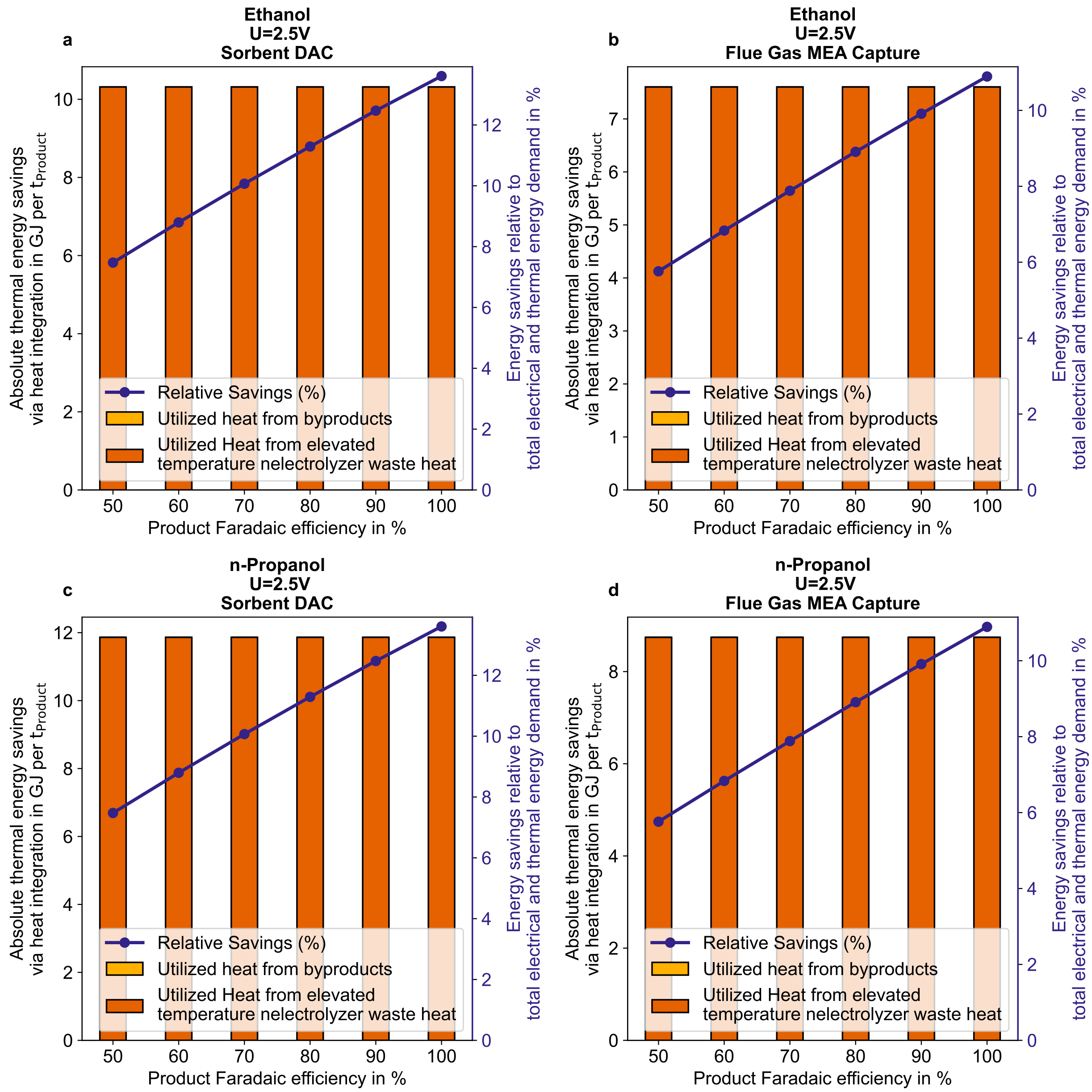

Figure S26: — **Potential thermal energy savings - Ethanol, n-propanol a,b** Potential thermal energy savings for ethanol as target product over the ethanol Faradaic efficiency for (a) direct air capture CO_2 supply and (b) point-source CO_2 capture. **c,d** Potential thermal energy savings for n-propanol as target product over the n-propanol Faradaic efficiency for (c) direct air capture CO_2 supply and (d) point-source CO_2 capture.

Current experimental results on Faradaic efficiency vary across the investigated CO_2 electroreduction products (compare Table S19). Given the current performance, there remains energy-reduction potential, even with sometimes low Faradaic efficiencies and point-source capture via a heat pump. The relative saving potential increases with smaller molecules and higher Faradaic efficiency. The saving potential for the case of 100 % Faradaic efficiency is given in Figure S28.

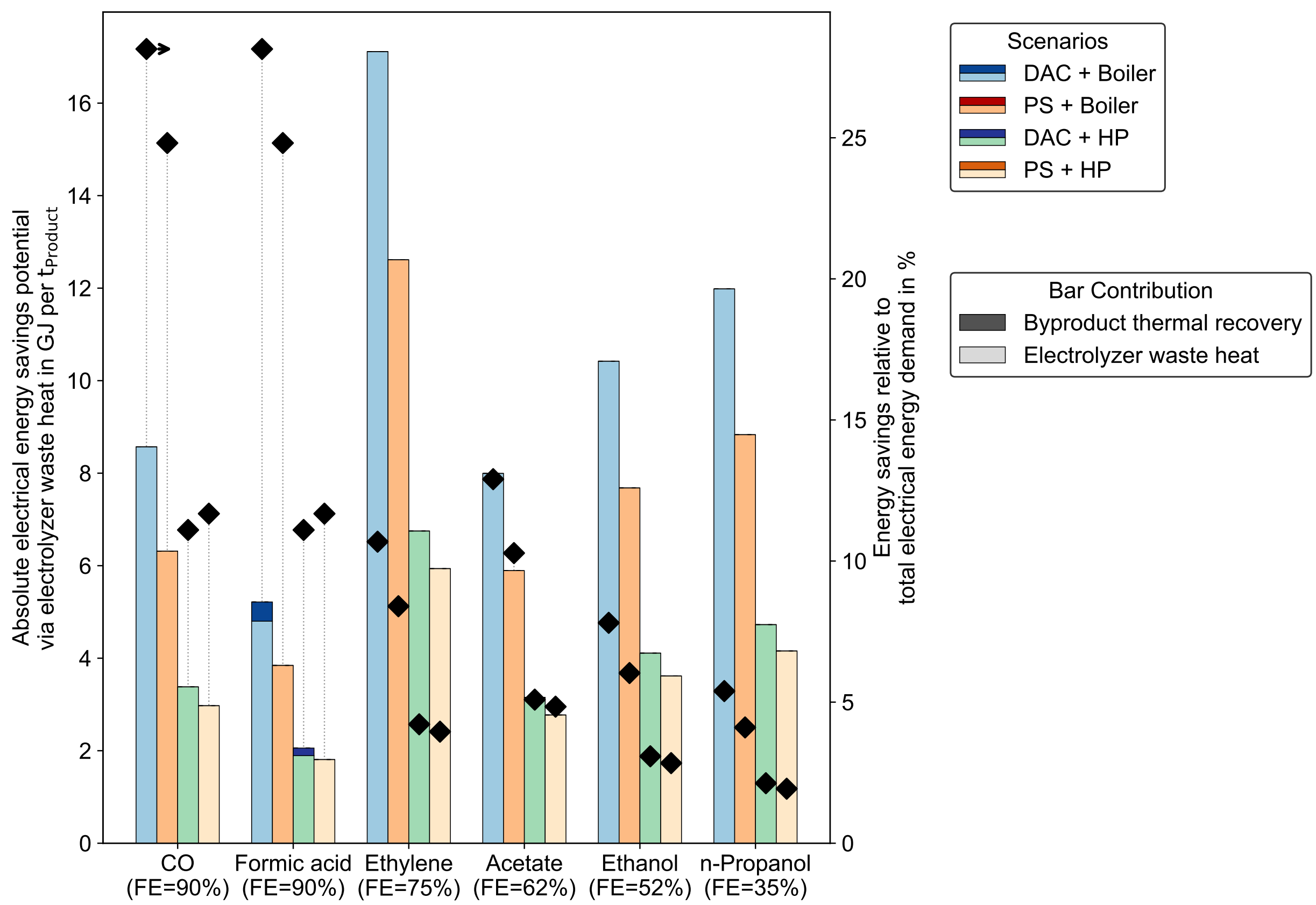

Figure S27: — **Electrical energy savings potential via electrolyzer waste heat utilization.** Absolute and relative electrical energy savings potential (electricity and heat supply) for the CO_2 electroreduction products CO, formic acid, ethylene, acetate, ethanol, and n-propanol for a cell voltage of 2.5 V. The utilization of electrolyzer waste heat and the savings in heating utility are compared to the total electricity demand. For the CO_2 capture, a DAC system [97] and a solvent-based point-source capture [201–203] are considered. For the heat supply, an electric boiler and a heat pump are compared. The remaining energy demand for the CO_2 supply is marked in white. The Faradaic efficiency is adapted from current experimentally reported results (Table S19).

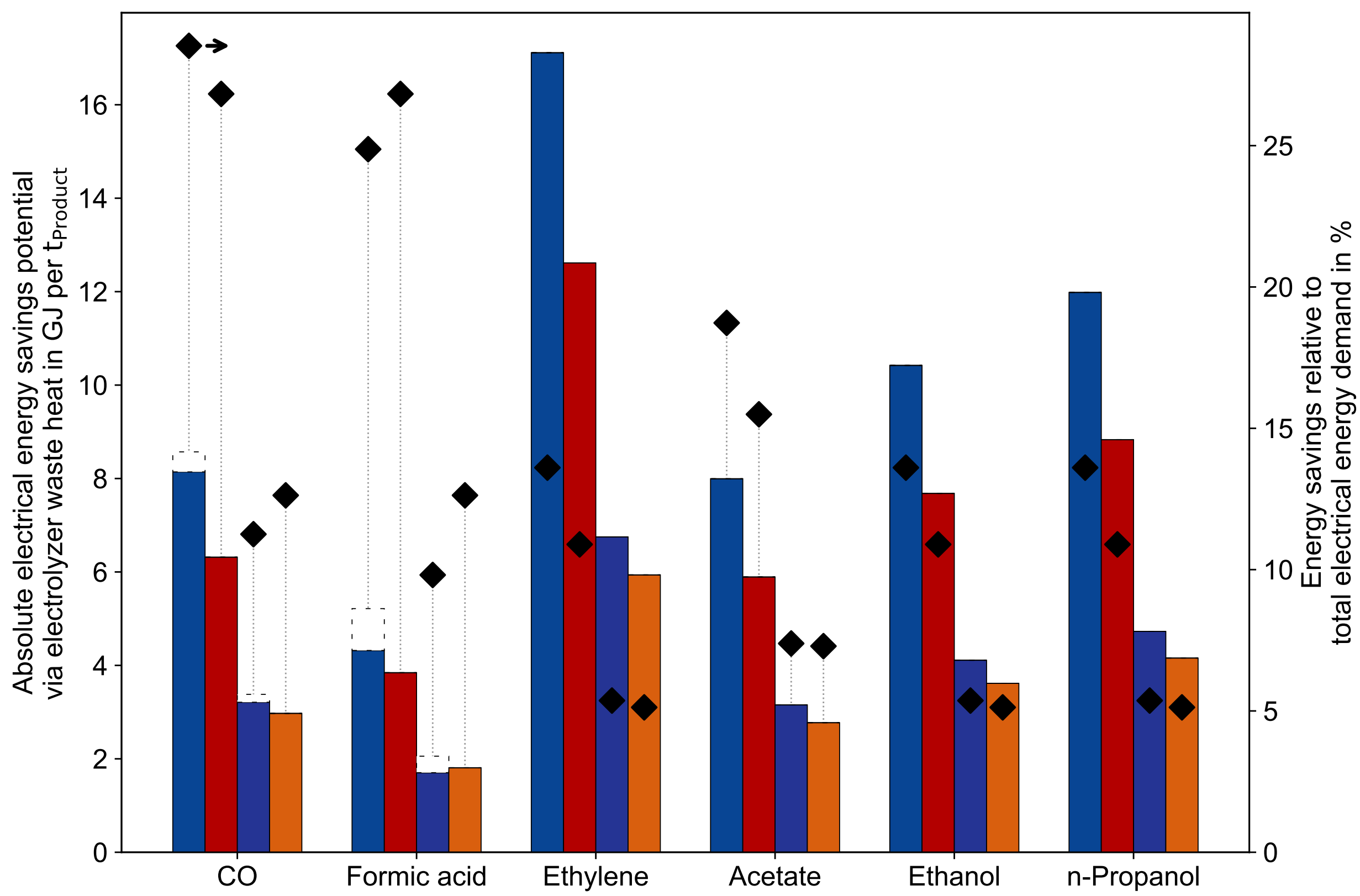

Figure S28: — **Maximum electrical energy savings potential via electrolyzer waste heat utilization.** Absolute and relative electrical energy savings potential (electricity and heat supply) for the CO_2 electroreduction products CO, formic acid, ethylene, acetate, ethanol, and n-propanol for a cell voltage of 2.5 V and Faradaic efficiency of 100 %. The utilization of electrolyzer waste heat and the savings in heating utility are compared to the total electricity demand. For the CO_2 capture, a DAC system [97] and a solvent-based point-source capture [201–203] are considered. For the heat supply, an electric boiler and a heat pump are compared. The remaining energy demand for the CO_2 supply is marked in white.

S7. Discussion

The main manuscript focuses on the benefits of higher electrolyzer temperatures for thermal integration and energy demand. This section provides further discussion on the case study and the process design.

The climate change impacts analysis demonstrated that the heating system must be decarbonized, especially if the electrolyzer is operated at low temperature. This need for heat decarbonization comes on top of decarbonizing the electricity system, which supplies the electrolyzer's main energy demand. A decarbonized electricity system with an electrified utility system could reduce the climate change impacts of 1-butene production by up to 96 % compared to using the current average US electricity grid mix (ideal case, Section S6.4).

Energy losses to cooling and carbon losses to byproducts are crucial factors to consider when designing CO_2 electroreduction processes. Carbon loss to liquid byproducts in aqueous solutions poses a separation challenge (Section S6.2, Section S6.6). Future research in the field will be needed. Such studies could build on the assessment of gas separations by Moore et al. [3] relevant to CO_2 electrolyzers and expand the scope to combined technology assessments of both liquid and gas separation systems for CO_2 electroreduction [46]. Furthermore, elevated pressure can enhance CO_2 electrolyzer performance and aid pressure-based separation, but may shift the product distribution [182]. Further research is needed to understand these effects on both the electrolyzer and the separation system.

Currently, 1-butene is primarily obtained as a byproduct from crackers or via catalytic dehydrogenation of n-butane or butanol [204]. Alternative production typically uses the intermediate ethylene, with ethylene dimerization being a mature process [25, 28, 29] and novel process alternatives being investigated [26, 27, 38]. Emerging pathways to ethylene start from CO_2, with the electrocatalytic conversion being more mature than the thermocatalytic conversion of CO_2 to ethylene [205]. Alternative multi-step pathways involve CO_2 hydrogenation to methanol, followed by methanol-to-olefins, or bio-based syngas routes. The pathway of this study prevents these multi-step processes with multiple separation steps and byproducts [205]. However, a direct comparison is challenging given the multiple parameters, assumptions, and technology readiness levels involved. A system-level combined feedstock and pathway analysis could close this gap by guiding research and development of key synthesis steps [206].

Bibliography

[1] M. G. Lee, X.-Y. Li, A. Ozden, J. Wicks, P. Ou, Y. Li, R. Dorakhan, J. Lee, H. K. Park, J. W. Yang, B. Chen, J. Abed, R. dos Reis, G. Lee, J. E. Huang, T. Peng, Y.-H. Chin, D. Sinton, and E. H. Sargent. Selective synthesis of butane from carbon monoxide using cascade electrolysis and thermocatalysis at ambient conditions. *Nature Catalysis*, 6(4):310–318, 2023. DOI: 10.1038/s41929-023-00937-0.

[2] T. Alerte, J. P. Edwards, C. M. Gabardo, C. P. O'Brien, A. Gaona, J. Wicks, A. Obradović, A. Sarkar, S. A. Jaffer, H. L. MacLean, D. Sinton, and E. H. Sargent. Downstream of the CO_2 Electrolyzer: Assessing the Energy Intensity of Product Separation. *ACS Energy Letters*, 6(12):4405–4412, 2021. DOI: 10.1021/acsenergylett.1c02263.

[3] T. Moore, D. I. Oyarzun, W. Li, T. Y. Lin, M. Goldman, A. A. Wong, S. A. Jaffer, A. Sarkar, S. E. Baker, E. B. Duoss, and C. Hahn. Electrolyzer energy dominates separation costs in state-of-the-art CO_2 electrolyzers: Implications for single-pass CO_2 utilization. *Joule*, 7(4):782–796, 2023. DOI: 10.1016/j.joule.2023.03.015.

[4] H. Al-Kalbani, J. Xuan, S. García, and H. Wang. Comparative energetic assessment of methanol production from CO_2: Chemical versus electrochemical process. *Applied Energy*, 165:1–13, 2016. DOI: 10.1016/j.apenergy.2015.12.027.

[5] A. Sarswat, D. S. Sholl, and R. P. Lively. Achieving order of magnitude increases in CO_2 reduction reaction efficiency by product separations and recycling. *Sustainable Energy & Fuels*, 6(20):4598–4604, 2022. DOI: 10.1039/D2SE01156E.

[6] M. Ramdin, A. R. T. Morrison, M. de Groen, R. van Haperen, R. de Kler, E. Irtem, A. T. Laitinen, L. J. P. van den Broeke, T. Breugelmans, J. P. M. Trusler, W. de Jong, and T. J. H. Vlugt. High-Pressure Electrochemical Reduction of CO_2 to Formic Acid/Formate: Effect of pH on the Downstream Separation Process and Economics. *Industrial & Engineering Chemistry Research*, 58(51):22718–22740, 2019. DOI: 10.1021/acs.iecr.9b03970.

[7] T. Alerte, A. Gaona, J. P. Edwards, C. M. Gabardo, C. P. O'Brien, J. Wicks, L. Bonnenfant, A. S. Rasouli, D. Young, J. Abed, L. Kershaw, Y. C. Xiao, A. Sarkar, S. A. Jaffer, M. W. Schreiber, D. Sinton, H. L. MacLean, and E. H. Sargent. Scale-Dependent Techno-Economic Analysis of CO_2 Capture and Electroreduction to Ethylene. *ACS Sustainable Chemistry & Engineering*, 11(43):15651–15662, 2023. DOI: 10.1021/acssuschemeng.3c04373.

[8] M. Rosental, T. Fröhlich, and A. Liebich. Life Cycle Assessment of Carbon Capture and Utilization for the Production of Large Volume Organic Chemicals. *Frontiers in Climate*, 2:586199, 2020. DOI: 10.3389/fclim.2020.586199.

[9] K. Ogura. Electrochemical reduction of carbon dioxide to ethylene: Mechanistic approach. *Journal of CO2 Utilization*, 1:43–49, 2013. DOI: 10.1016/j.jcou.2013.03.003.

[10] R. I. Masel, Z. Liu, H. Yang, J. J. Kaczur, D. Carrillo, S. Ren, D. Salvatore, and C. P. Berlinguette. An industrial perspective on catalysts for low-temperature CO_2 electrolysis. *Nature Nanotechnology*, 16(2):118–128, 2021. DOI: 10.1038/s41565-020-00823-x.

[11] C. P. O'Brien, R. K. Miao, A. Shayesteh Zeraati, G. Lee, E. H. Sargent, and D. Sinton. CO_2 Electrolyzers. *Chemical Reviews*, 124(7):3648–3693, 2024. DOI: 10.1021/acs.chemrev.3c00206.

[12] M. Jouny, W. Luc, and F. Jiao. General Techno-Economic Analysis of CO_2 Electrolysis Systems. *Industrial & Engineering Chemistry Research*, 57(6):2165–2177, 2018. DOI: 10.1021/acs.iecr.7b03514.

[13] S. Verma, B. Kim, H.-R. M. Jhong, S. Ma, and P. J. A. Kenis. A Gross-Margin Model for Defining Technoeconomic Benchmarks in the Electroreduction of CO_2. *ChemSusChem*, 9(15):1972–1979, 2016. DOI: 10.1002/cssc.201600394.

[14] H. Wu, L. Huang, J. Timoshenko, K. Qi, W. Wang, J. Liu, Y. Zhang, S. Yang, E. Petit, V. Flaud, J. Li, C. Salameh, P. Miele, L. Lajaunie, B. Roldán Cuenya, D. Rao, and D. Voiry. Selective and energy-efficient electrosynthesis of ethylene from CO_2 by tuning the valence of Cu catalysts through aryl diazonium functionalization. *Nature Energy*, 9(4):422–433, 2024. DOI: 10.1038/s41560-024-01461-6.

[15] C.-T. Dinh, T. Burdyny, M. G. Kibria, A. Seifitokaldani, C. M. Gabardo, F. P. García de Arquer, A. Kiani, J. P. Edwards, P. de Luna, O. S. Bushuyev, C. Zou, R. Quintero-Bermudez, Y. Pang, D. Sinton, and E. H. Sargent. CO_2 electroreduction to ethylene via hydroxide-mediated copper catalysis at an abrupt interface. *Science*, 360(6390): 783–787, 2018. DOI: 10.1126/science.aas9100.

[16] Membrane Properties and Characterization for Zero-Gap CO_2 Electrolyzers, 2021. URL `https://www.fuelcellstore.com/blog-section/membrane-properties-characterization-zero-gap-co2-electrolyzer`, [Online] (accessed on 2023-07-05).

[17] L. Yuan, S. Zeng, X. Zhang, X. Ji, and S. Zhang. Advances and challenges of electrolyzers for large-scale CO_2 electroreduction. *Materials Reports: Energy*, 3(1):100177, 2023. DOI: 10.1016/j.matre.2023.100177.

[18] L. Hoof, N. Thissen, K. Pellumbi, K. junge Puring, D. Siegmund, A. K. Mechler, and U.-P. Apfel. Hidden parameters for electrochemical carbon dioxide reduction in zero-gap electrolyzers. *Cell Reports Physical Science*, 3(4):100825, 2022. DOI: 10.1016/j.xcrp.2022.100825.

[19] M. S. Sajna, S. Zavahir, A. Popelka, P. Kasak, A. Al-Sharshani, U. Onwusogh, M. Wang, H. Park, and D. S. Han. Electrochemical system design for CO_2 conversion: A comprehensive review. *Journal of Environmental Chemical Engineering*, 11(5):110467, 2023. DOI: 10.1016/j.jece.2023.110467.

[20] M. Ramdin, B. de Mot, A. R. T. Morrison, T. Breugelmans, L. J. P. van den Broeke, J. P. M. Trusler, R. Kortlever, W. de Jong, O. A. Moultos, P. Xiao, P. A. Webley, and T. J. H. Vlugt. Electroreduction of CO_2/CO to C2 Products: Process Modeling, Downstream Separation, System Integration, and Economic Analysis. *Industrial & Engineering Chemistry Research*, 60(49):17862–17880, 2021. DOI: 10.1021/acs.iecr.1c03592.

[21] W. Choi, S. Park, W. Jung, H. Da Won, J. Na, and Y. J. Hwang. Origin of Hydrogen Incorporated into Ethylene during Electrochemical CO_2 Reduction in Membrane Electrode Assembly. *ACS Energy Letters*, 7(3):939–945, 2022. DOI: 10.1021/acsenergylett.1c02658.

[22] J. Sisler, S. Khan, A. H. Ip, M. W. Schreiber, S. A. Jaffer, E. R. Bobicki, C.-T. Dinh, and E. H. Sargent. Ethylene Electrosynthesis: A Comparative Techno-economic Analysis of Alkaline vs Membrane Electrode Assembly vs CO_2–CO–C_2H_4 Tandems. *ACS Energy Letters*, 6(3):997–1002, 2021. DOI: 10.1021/acsenergylett.0c02633.

[23] J.-Y. Lee, S. An, K. Cha, and T. Hur. Life cycle environmental and economic analyses of a hydrogen station with wind energy. *International Journal of Hydrogen Energy*, 35 (6):2213–2225, 2010. DOI: 10.1016/j.ijhydene.2009.12.082.

[24] S. Yamada and I. Ono. Production of Butene-1 from Ethylene. *Bulletin of The Japan Petroleum Institute*, 12:160–163, 1970.

[25] F. A. Al-Sherehy. IFP-SABIC process for the selective ethylene dimerization to butene-1. In M. Absi-Halabi, editor, *Catalysts in Petroleum Refining and Petrochemical Industries 1995, Proceedings of the 2nd International Conference on Catalysts in*

Petroleum refining and Petrochemical Industries, volume 100 of *Studies in Surface Science and Catalysis*, pages 515–523, New York, NY, USA, 1996. Elsevier. DOI: 10.1016/S0167-2991(96)80052-8.

[26] E. D. Metzger, C. K. Brozek, R. J. Comito, and M. Dincă. Selective Dimerization of Ethylene to 1-Butene with a Porous Catalyst. *ACS Central Science*, 2(3):148–153, 2016. DOI: 10.1021/acscentsci.6b00012.

[27] J. L. Mancuso, C. A. Gaggioli, L. Gagliardi, and C. H. Hendon. Singlet-to-Triplet Spin Transitions Facilitate Selective 1-Butene Formation during Ethylene Dimerization in Ni(II)-MFU-4 l. *The Journal of Physical Chemistry C*, 125(40):22036–22043, 2021. DOI: 10.1021/acs.jpcc.1c07658.

[28] H. Alenezi, S. Alwi, Z. A. Manan, and D. Zaidel. Recent developments on ethylene dimerization with focus on alphabutol optimization. *International Journal of Innovative Technology and Exploring Engineering*, 8(10):3969–3975, 2019. DOI: 10.35940/ijitee.J9936.0881019.

[29] H. Alenezi, Z. Haidar, and M. Das. Optimization of Parameters Using Taguchi Orthogonal Array Design for an Intensified Per–Pass Conversion of Alphabutol® Technology in Butene–1 Production. *International Journal of Chemical Engineering*, 2022:1699196, 2022. DOI: 10.1155/2022/1699196.

[30] E. Ali and K. Al-humaizi. Temperature Control of Ethylene to Butene-1 Dimerization Reactor. *Industrial & Engineering Chemistry Research*, 39(5):1320–1329, 2000. DOI: 10.1021/ie9903846.

[31] A. Choudhary, A. Shetty, and S. Oswal. Dynamic Simulation of Reactor to Produce 1-Butene by Dimerization of Ethylene. *International Journal of Scientific & Engineering Research*, 3(6):1–7, 2012.

[32] A. Forestière, H. Olivier-Bourbigou, and L. Saussine. Oligomerization of Monoolefins by Homogeneous Catalysts. *Oil & Gas Science and Technology - Revue de l'IFP*, 64 (6):649–667, 2009. DOI: 10.2516/ogst/2009027.

[33] S. V. Kotov and I. N. Kankaeva. Commercial production and principal trends in the use of 1-butene. *Chemistry and Technology of Fuels and Oils*, 30(5):240–245, 1994. DOI: 10.1007/BF00727599.

[34] AlphaButol: Process Flow DIagram, 2022. URL https://www.mogas.com/en-us/resources/media-centre/public/documents/pfds/pfd-alphabutol, [Online] (accessed on 2022-07-29).

[35] S. Mohammadzade Fard, M. Farsi, and M. R. Rahimpour. Optimization of ethylene dimerization in a bubble column reactor based on coupling kinetic and equilibrium models. *Chemical Engineering Research and Design*, 174:357–364, 2021. DOI: 10.1016/j.cherd.2021.07.030.

[36] M. Naeem and Al-Rabiah, A. A., Mughees, Washif. Process Simulation of 1-Butene and N-Butane Separation By Extractive Distillation. *International Journal of Engineering Research & Technology*, 3(6):747–750, 2014. DOI: 10.17577/ijertv3is060878.

[37] F. Ng and D. C. Creaser. Ethylene Dimerization: Kinetics and Selectivity for 1-Butene. In *Progress in Catalysis, Proceedings of the 12th Canadian Symposium on Catalysis*, volume 73 of *Studies in Surface Science and Catalysis*, pages 123–131. Elsevier, 1992. DOI: 10.1016/S0167-2991(08)60805-8.

[38] Z. Xu, J. P. Chada, L. Xu, D. Zhao, D. C. Rosenfeld, J. L. Rogers, I. Hermans, M. Mavrikakis, and G. W. Huber. Ethylene Dimerization and Oligomerization to 1-Butene and Higher Olefins with Chromium-Promoted Cobalt on Carbon Catalyst. *ACS Catalysis*, 8(3):2488–2497, 2018. DOI: 10.1021/acscatal.7b03205.

[39] W. Chung, W. Jeong, J. Lee, J. Kim, K. Roh, and J. H. Lee. Electrification of CO_2 conversion into chemicals and fuels: Gaps and opportunities in process systems engineering. *Computers & Chemical Engineering*, 170:108106, 2023. DOI: 10.1016/j.compchemeng.2022.108106.

[40] M. Heßelmann, H. Minten, T. Geissler, R. G. Keller, A. Bardow, and M. Wessling. Why Membranes Matter: Ion Exchange Membranes in Holistic Process Optimization of Electrochemical CO_2 Reduction. *Advanced Sustainable Systems*, 7(7):2300077, 2023. DOI: 10.1002/adsu.202300077.

[41] J. B. Greenblatt, D. J. Miller, J. W. Ager, F. A. Houle, and I. D. Sharp. The Technical and Energetic Challenges of Separating (Photo)Electrochemical Carbon Dioxide Reduction Products. *Joule*, 2(3):381–420, 2018. DOI: 10.1016/j.joule.2018.01.014.

[42] H. Lee, S. Kwon, N. Park, S. G. Cha, E. Lee, T.-H. Kong, J. Cha, and Y. Kwon. Scalable Low-Temperature CO_2 Electrolysis: Current Status and Outlook. *JACS Au*, 4(9):3383–3399, 2024. DOI: 10.1021/jacsau.4c00583.

[43] P. de Luna, C. Hahn, D. Higgins, S. A. Jaffer, T. F. Jaramillo, and E. H. Sargent. What would it take for renewably powered electrosynthesis to displace petrochemical processes? *Science*, 364(6438):eaav3506, 2019. DOI: 10.1126/science.aav3506.

[44] M. J. Orella, S. M. Brown, M. E. Leonard, Y. Román-Leshkov, and F. R. Brushett. A General Technoeconomic Model for Evaluating Emerging Electrolytic Processes. *Energy Technology*, 8(11):1900994, 2020. DOI: 10.1002/ente.201900994.

[45] A. Paturska, M. Repele, and G. Bazbauers. Economic Assessment of Biomethane Supply System based on Natural Gas Infrastructure. *Energy Procedia*, 72:71–78, 2015. DOI: 10.1016/j.egypro.2015.06.011.

[46] B. Pribyl-Kranewitter, A. Beard, C. L. Gîjiu, D. Dinculescu, and T. J. Schmidt. Influence of low-temperature electrolyser design on economic and environmental potential of CO and HCOOH production: A techno-economic assessment. *Renewable and Sustainable Energy Reviews*, 154:111807, 2022. DOI: 10.1016/j.rser.2021.111807.

[47] T. Gao, B. Xia, K. Yang, Di Li, T. Shao, S. Chen, Q. Li, and J. Duan. Techno-economic Analysis and Carbon Footprint Accounting for Industrial CO_2 Electrolysis Systems. *Energy & Fuels*, 37(23):17997–18008, 2023. DOI: 10.1021/acs.energyfuels.3c01581.

[48] A. Venkataraman, H. Song, V. D. Brandão, C. Ma, M. S. Casajus, C. A. Fernandez Otero, C. Sievers, M. C. Hatzell, S. S. Bhargava, S. S. Arora, C. Villa, S. Dhingra, and S. Nair. Process and techno-economic analyses of ethylene production by electrochemical reduction of aqueous alkaline carbonates. *Nature Chemical Engineering*, 1(11):710–723, 2024. DOI: 10.1038/s44286-024-00137-y.

[49] J. Wyndorps, H. Ostovari, and N. von der Assen. Is electrochemical CO_2 reduction the future technology for power-to-chemicals? An environmental comparison with H_2-based pathways. *Sustainable Energy & Fuels*, 5(22):5748–5761, 2021. DOI: 10.1039/D1SE00975C.

[50] J. B. Dahmus and T. G. Gutowski. What gets recycled: an information theory based model for product recycling. *Environmental Science & Technology*, 41(21):7543–7550, 2007. DOI: 10.1021/es062254b.

[51] K. Z. House, A. C. Baclig, M. Ranjan, E. A. van Nierop, J. Wilcox, and H. J. Herzog. Economic and energetic analysis of capturing CO_2 from ambient air. *Proceedings of the National Academy of Sciences*, 108(51):20428–20433, 2011. DOI: 10.1073/pnas.1012253108.

[52] S. Kibria Nabil, S. McCoy, and M. G. Kibria. Comparative life cycle assessment of electrochemical upgrading of CO_2 to fuels and feedstocks. *Green Chemistry*, 23(2): 867–880, 2021. DOI: 10.1039/D0GC02831B.

[53] A. Ozden, F. P. García de Arquer, J. E. Huang, J. Wicks, J. Sisler, R. K. Miao, C. P. O'Brien, G. Lee, X. Wang, A. H. Ip, E. H. Sargent, and D. Sinton. Carbon-efficient carbon dioxide electrolysers. *Nature Sustainability*, 5(7):563–573, 2022. DOI: 10.1038/s41893-022-00879-8.

[54] H. Shin, K. U. Hansen, and F. Jiao. Techno-economic assessment of low-temperature carbon dioxide electrolysis. *Nature Sustainability*, 4(10):911–919, 2021. DOI: 10.1038/s41893-021-00739-x.

[55] A. Ozden, Y. Wang, F. Li, M. Luo, J. Sisler, A. Thevenon, A. Rosas-Hernández, T. Burdyny, Y. Lum, H. Yadegari, T. Agapie, J. C. Peters, E. H. Sargent, and D. Sinton. Cascade CO_2 electroreduction enables efficient carbonate-free production of ethylene. *Joule*, 5(3):706–719, 2021. DOI: 10.1016/j.joule.2021.01.007.

[56] S. C. Da Cunha and J. Resasco. Maximizing single-pass conversion does not result in practical readiness for CO_2 reduction electrolyzers. *Nature Communications*, 14(1): 5513, 2023. DOI: 10.1038/s41467-023-41348-w.

[57] M. Scholz, B. Frank, F. Stockmeier, S. Falß, and M. Wessling. Techno-economic Analysis of Hybrid Processes for Biogas Upgrading. *Industrial & Engineering Chemistry Research*, 52(47):16929–16938, 2013. DOI: 10.1021/ie402660s.

[58] K. Bareiß, C. de La Rua, M. Möckl, and T. Hamacher. Life cycle assessment of hydrogen from proton exchange membrane water electrolysis in future energy systems. *Applied Energy*, 237(2):862–872, 2019. DOI: 10.1016/j.apenergy.2019.01.001.

[59] S. C. D'Angelo, S. Cobo, V. Tulus, A. Nabera, A. J. Martín, J. Pérez-Ramírez, and G. Guillén-Gosálbez. Planetary Boundaries Analysis of Low-Carbon Ammonia Production Routes. *ACS Sustainable Chemistry & Engineering*, 9(29):9740–9749, 2021. DOI: 10.1021/acssuschemeng.1c01915.

[60] J. Koj, C. Wulf, A. Schreiber, and P. Zapp. Site-Dependent Environmental Impacts of Industrial Hydrogen Production by Alkaline Water Electrolysis. *Energies*, 10(7):860, 2017. DOI: 10.3390/en10070860.

[61] F. Wu, M. D. Argyle, P. A. Dellenback, and M. Fan. Progress in O_2 separation for oxy-fuel combustion–A promising way for cost-effective CO_2 capture: A review. *Progress in Energy and Combustion Science*, 67:188–205, 2018. DOI: 10.1016/j.pecs.2018.01.004.

[62] Z. Liu, P. Hao, S. Li, X. Zhu, Y. Shi, and N. Cai. Simulation and energy consumption comparison of gas purification system based on elevated temperature pressure swing adsorption in ammonia synthetic system. *Adsorption*, 26(7):1239–1252, 2020. DOI: 10.1007/s10450-020-00224-5.

[63] J. Perez-Carbajo, I. Matito-Martos, S. R. G. Balestra, M. N. Tsampas, M. C. M. van de Sanden, J. A. Delgado, V. I. Águeda, P. J. Merkling, and S. Calero. Zeolites for CO_2-CO-O_2 Separation to Obtain CO_2-Neutral Fuels. *ACS Applied Materials & Interfaces*, 10(24):20512–20520, 2018. DOI: 10.1021/acsami.8b04507.

[64] N. Kohlheb, M. Wluka, A. Bezama, D. Thrän, A. Aurich, and R. A. Müller. Environmental-Economic Assessment of the Pressure Swing Adsorption Biogas Upgrading Technology. *BioEnergy Research*, 14(3):901–909, 2021. DOI: 10.1007/s12155-020-10205-9.

[65] M. Jaschik, M. Tańczyk, A. Janusz-Cygan, A. Wojdyła, and K. Warmuziński. The Separation of Cabo Dioxide from $CO_2/N_2/O_2$ Mixtures using Polyimide and Polysulphone Membranes. *Chemical and Process Engineering*, 39(4):449–456, 2018. DOI: 10.24425/122962.

[66] W. Wang, L. Wu, Z. Li, Y. Fang, J. Ding, and J. Xiao. An Overview of Adsorbents in the Rotary Desiccant Dehumidifier for Air Dehumidification. *Drying Technology*, 31 (12):1334–1345, 2013. DOI: 10.1080/07373937.2013.792094.

[67] M. Djaeni, D. Q. A'yuni, M. Alhanif, C. L. Hii, and A. C. Kumoro. Air dehumidification with advance adsorptive materials for food drying: A critical assessment for future prospective. *Drying Technology*, 39(11):1648–1666, 2021. DOI: 10.1080/07373937.2021.1885042.

[68] S. Kumar, N. Singh, and R. Prasad. Anhydrous ethanol: A renewable source of energy. *Renewable and Sustainable Energy Reviews*, 14(7):1830–1844, 2010. DOI: 10.1016/j.rser.2010.03.015.

[69] X. Zheng, K. Chen, and Z. Lin. Synthesis and Characterization of Alginate–Silica Gel Composites for Adsorption Dehumidification. *Industrial & Engineering Chemistry Research*, 59(13):5760–5767, 2020. DOI: 10.1021/acs.iecr.9b06157.

[70] M. Mekala, B. Neerudi, P. R. Are, R. Surakasi, G. Manikandan, V. R. Kakara, and A. A. Dhumal. Water Removal from an Ethanol-Water Mixture at Azeotropic Condition by Adsorption Technique. *Adsorption Science & Technology*, 2022:1–10, 2022. DOI: 10.1155/2022/8374471.

[71] R. Krishna and J. M. van Baten. Water/Alcohol Mixture Adsorption in Hydrophobic Materials: Enhanced Water Ingress Caused by Hydrogen Bonding. *ACS Omega*, 5(43): 28393–28402, 2020. DOI: 10.1021/acsomega.0c04491.

[72] M. Klauck, J. Guhlmann, T. Hähnel, M. Hauser, and G. Kalies. Liquid adsorption and immersion of two alcohol–water mixtures on carbon molecular sieves. *Adsorption*, 28 (3-4):137–147, 2022. DOI: 10.1007/s10450-022-00359-7.

[73] M. Simo, S. Sivashanmugam, C. J. Brown, and V. Hlavacek. Adsorption/Desorption of Water and Ethanol on 3A Zeolite in Near-Adiabatic Fixed Bed. *Industrial & Engineering Chemistry Research*, 48(20):9247–9260, 2009. DOI: 10.1021/ie900446v.

[74] B. Tanchuk, J. A. Sawada, S. Rezaei, and S. M. Kuznicki. Adsorptive drying of CO_2 using low grade heat and humid, ambient air. *Separation and Purification Technology*, 120:354–361, 2013. DOI: 10.1016/j.seppur.2013.10.026.

[75] M. Netusil and P. Ditl. Comparison of three methods for natural gas dehydration. *Journal of Natural Gas Chemistry*, 20(5):471–476, 2011. DOI: 10.1016/S1003-9953(10)60218-6.

[76] J.-C. Li, Y. Cao, S. Xu, P. He, L. Wang, X. Hui, and H.-Q. Li. Catalytic hydrogenation of CO_2 to produce lower alcohols and ether over Co–Cu–Zn–Al catalyst. *Greenhouse Gases: Science and Technology*, 11:1180–1190, 2021. DOI: 10.1002/ghg.2135.

[77] P. M. Osterberg, J. K. Niemeier, C. J. Welch, J. M. Hawkins, J. R. Martinelli, T. E. Johnson, T. W. Root, and S. S. Stahl. Experimental Limiting Oxygen Concentrations for Nine Organic Solvents at Temperatures and Pressures Relevant to Aerobic Oxidations in the Pharmaceutical Industry. *Organic Process Research & Development*, 19 (11):1537–1543, 2015. DOI: 10.1021/op500328f.

[78] P. Glarborg and L. L. B. Bentzen. Chemical Effects of a High CO_2 Concentration in Oxy-Fuel Combustion of Methane. *Energy & Fuels*, 22(1):291–296, 2008. DOI: 10.1021/ef7005854.

[79] A. Cardona-Vargas, D. Valencia, C. E. Arrieta, and A. Amell. Combustion of a hydrogen/carbon monoxide/carbon dioxide mixture in hot and diluted streams. *Journal*

of Physics: Conference Series, 1708(1):012013, 2020. DOI: 10.1088/1742-6596/1708/1/012013.

[80] G. Lu, Z. Wang, U. H. Bhatti, and X. Fan. Recent progress in carbon dioxide capture technologies: A review. *Clean Energy Science and Technology*, 1(1), 2023. DOI: 10.18686/cest.v1i1.32.

[81] S. Talei, D. Fozer, P. S. Varbanov, A. Szanyi, and P. Mizsey. Oxyfuel Combustion Makes Carbon Capture More Efficient. *ACS Omega*, 9(3):3250–3261, 2024. DOI: 10.1021/acsomega.3c05034.

[82] P. Li, Z. Liu, M. Li, Y. Zhao, X. Li, S. Wan, Y. Ma, and Y. He. Investigation on the limiting oxygen concentration of combustible gas at high pressures and temperatures during oil recovery process. *Energy*, 215(9–10):119157, 2021. DOI: 10.1016/j.energy.2020.119157.

[83] R. E. Hayes and S. T. Kolaczkowski. *Introduction to catalytic combustion*. Gordon and Breach, Amsterdam, The Netherlands, 1997. DOI: 10.1201/19780203750186.

[84] M. A. Nemitallah, M. A. Habib, H. M. Badr, S. A. Said, A. Jamal, R. Ben-Mansour, E. M. A. Mokheimer, and K. Mezghani. Oxy-fuel combustion technology: current status, applications, and trends. *International Journal of Energy Research*, 41(12):1670–1708, 2017. DOI: 10.1002/er.3722.

[85] B. Buhre, L. K. Elliott, C. D. Sheng, R. P. Gupta, and T. F. Wall. Oxy-fuel combustion technology for coal-fired power generation. *Progress in Energy and Combustion Science*, 31(4):283–307, 2005. DOI: 10.1016/j.pecs.2005.07.001.

[86] Y. A. Ahmed, I. Lazakis, and G. Mallouppas. Advancements and challenges of onboard carbon capture and storage technologies for the maritime industry: a comprehensive review. *Marine Systems & Ocean Technology*, 20(1):13, 2025. DOI: 10.1007/s40868-024-00161-w.

[87] E. V. Jithin, G. Raghuram, T. V. Keshavamurthy, R. K. Velamati, C. Prathap, and R. J. Varghese. A review on fundamental combustion characteristics of syngas mixtures and feasibility in combustion devices. *Renewable and Sustainable Energy Reviews*, 146 (7565):111178, 2021. DOI: 10.1016/j.rser.2021.111178.

[88] G. Wernet, C. Bauer, B. Steubing, J. Reinhard, E. Moreno-Ruiz, and B. Weidema. The ecoinvent database version 3 (part I): overview and methodology. *The International*

Journal of Life Cycle Assessment, 21(9):1218–1230, 2016. DOI: 10.1007/s11367-016-1087-8.

[89] B. Linnhoff and J. R. Flower. Synthesis of heat exchanger networks: II. Evolutionary generation of networks with various criteria of optimality. *AIChE Journal*, 24(4):642–654, 1978. DOI: 10.1002/aic.690240412.

[90] R. Smith. *Chemical process design and integration*. Wiley, Chichester, West Sussex, UK, 2nd edition, 2016.

[91] T. F. Yee and I. E. Grossmann. Simultaneous optimization models for heat integration—II. Heat exchanger network synthesis. *Computers & Chemical Engineering*, 14(10):1165–1184, 1990. DOI: 10.1016/0098-1354(90)85010-8.

[92] R. H. Byrd, J. Nocedal, and R. A. Waltz. KNITRO: An Integrated Package for Nonlinear Optimization. In G. Di Pillo and M. Roma, editors, *Large-scale nonlinear optimization*, volume 83 of *Nonconvex optimization and its applications*, pages 35–59. Springer, New York, NY, USA, 2006. DOI: 10.1007/0-387-30065-1_4.

[93] A. Renaud and A. Marié. Knitro, 2020. URL `https://www.artelys.com/solvers/knitro/`, [Online] (accessed on 2026-02-20).

[94] D. Vázquez and G. Guillén-Gosálbez. Process design within planetary boundaries: Application to CO_2 based methanol production. *Chemical Engineering Science*, 246 (6):116891, 2021. DOI: 10.1016/j.ces.2021.116891.

[95] R. Lakner, Á. Orosz, B. S. How, and F. Friedler. Synthesis of multiperiod heat exchanger networks: n-best networks with variable approach temperature. *Thermal Science and Engineering Progress*, 42(8):101912, 2023. DOI: 10.1016/j.tsep.2023.101912.

[96] G. P. Towler and R. K. Sinnott. *Chemical engineering design: Principles, practice and economics of plant and process design*. Butterworth-Heinemann, Amsterdam, The Netherlands, 3rd edition, 2021. DOI: 10.1016/C2019-0-02025-0.

[97] S. Deutz and A. Bardow. Life-cycle assessment of an industrial direct air capture process based on temperature–vacuum swing adsorption. *Nature Energy*, 6(2):203–213, 2021. DOI: 10.1038/s41560-020-00771-9.

[98] Aspen Technology Inc. Aspen HYSYS, 2019. URL `https://www.aspentech.com/en/products/engineering/aspen-hysys`, [Online] (accessed on 2023-12-02).

[99] M. H. Barecka, J. W. Ager, and A. A. Lapkin. Techno-economic assessment of emerging CO_2 electrolysis technologies. *STAR protocols*, 2(4):100889, 2021. DOI: 10.1016/j.xpro.2021.100889.

[100] R. Niekerk and R. Manita. Thermal management in green hydrogen production: design considerations, 2022. URL `https://hydrogentechworld.com/thermal-management-in-green-hydrogen-production-design-considerations`, [Online] (accessed on 2023-08-02).

[101] Optimizing electrolyser cooling processes, 2023. URL `https://www.alfalaval.com/industries/energy-and-utilities/sustainablesolutions/sustainable-solutions/clean-energy/clean-hydrogen/optimizing-electrolyser-cooling-processes/`, [Online] (accessed on 2023-08-02).

[102] Solutions for cooling of hydrogen, 2023. URL `https://www.lauda.de/en/temperature-control-solutions-by-sectors/hydrogen`, [Online] (accessed on 2023-08-02).

[103] Integrated Electrolyzer & Heat Pump Solution, 2023. URL `https://www.siemens-energy.com/global/en/offerings/renewable-energy/hydrogen-solutions/integrated-electrolyzer-heat-pump-solution.html`, [Online] (accessed on 2023-08-02).

[104] A. Hodges, A. L. Hoang, G. Tsekouras, K. Wagner, C.-Y. Lee, G. F. Swiegers, and G. G. Wallace. A high-performance capillary-fed electrolysis cell promises more cost-competitive renewable hydrogen. *Nature Communications*, 13(1):1304, 2022. DOI: 10.1038/s41467-022-28953-x.

[105] E. Bellos and C. Tzivanidis. CO_2 Transcritical Refrigeration Cycle with Dedicated Subcooling: Mechanical Compression vs. Absorption Chiller. *Applied Sciences*, 9(8): 1605, 2019. DOI: 10.3390/app9081605.

[106] M. Deymi-Dashtebayaz, A. Sulin, T. Ryabova, I. Sankina, M. Farahnak, and R. Nazeri. Energy, exergoeconomic and environmental optimization of a cascade refrigeration system using different low GWP refrigerants. *Journal of Environmental Chemical Engineering*, 9(6):106473, 2021. DOI: 10.1016/j.jece.2021.106473.

[107] M. J. S. Zuberi, A. Hasanbeigi, and W. R. Morrow. Electrification of Boilers in U.S. Manufacturing, 2021. URL `https://eta-publications.lbl.gov/publications/electrification-boilers-us`, [Online] (accessed on 2023-04-19).

[108] PEP Yearbook: Process Economics, 2017 Q2 data, 2017. URL https://www.spglobal.com/energy/en/products-solutions/chemicals/chemical-process-economics-program-pep, [Online] (accessed on 2026-04-02).

[109] Chemical Properties of 1-Butene (CAS 106-98-9), 2023. URL https://www.chemeo.com/cid/13-178-0/1-Butene, [Online] (accessed on 2026-04-29).

[110] Chemical Properties of Carbon dioxide (CAS 124-38-9), 2023. URL https://www.chemeo.com/cid/25-906-8/Carbon-dioxide, [Online] (accessed on 2026-04-29).

[111] Chemical Properties of Water (CAS 7732-18-5), 2023. URL https://www.chemeo.com/cid/69-886-3/Water, [Online] (accessed on 2026-04-29).

[112] J. Koh, D. Yoon, and C. H. Oh. Simple Electrolyzer Model Development for High-Temperature Electrolysis System Analysis Using Solid Oxide Electrolysis Cell. *Journal of Nuclear Science and Technology*, 47(7):599–607, 2010. DOI: 10.1080/18811248.2010.9720957.

[113] J. L. Valverde, V. R. Ferro, and A. Giroir-Fendler. Automation in the simulation of processes with Aspen HYSYS: An academic approach. *Computer Applications in Engineering Education*, 31(2):376–388, 2023. DOI: 10.1002/cae.22589.

[114] E. I. Sanchez Medina. Aspen HYSYS-Python connection using spreadsheets, 2021. URL https://github.com/edgarsmdn/Aspen_HYSYS_Python, [Online] (accessed on 2023-12-02).

[115] D. Vázquez Vázquez. PySIS, 2023. URL https://github.com/DanielVazVaz/PySIS, [Online] (accessed on 2023-12-02).

[116] P. Heck. pina: A lightweight pinch analysis package, 2019. URL https://github.com/FitzOReilly/pina, [Online] (accessed on 2023-04-06).

[117] C. Mutel. Brightway: An open source framework for Life Cycle Assessment. *Journal of Open Source Software*, 2(12):236, 2017. DOI: 10.21105/joss.00236.

[118] M. Pérez-Fortes, J. C. Schöneberger, A. Boulamanti, and E. Tzimas. Methanol synthesis using captured CO_2 as raw material: Techno-economic and environmental assessment. *Applied Energy*, 161(11):718–732, 2016. DOI: 10.1016/j.apenergy.2015.07.067.

[119] C. Maxwell. Cost Indices, 2020. URL https://toweringskills.com/financial-analysis/cost-indices/, [Online] (accessed on 2023-08-01).

[120] Chemical Engineering Plant Cost Index, 2023. URL https://personalpages.manchester.ac.uk/staff/tom.rodgers/Interactive_graphs/CEPCI.html?reactors/CEPCI/index.html, [Online] (accessed on 2023-04-21).

[121] J. Young, N. McQueen, C. Charalambous, S. Foteinis, O. Hawrot, M. Ojeda, H. Pilorgé, J. Andresen, P. Psarras, P. Renforth, S. Garcia, and M. van der Spek. The cost of direct air capture and storage can be reduced via strategic deployment but is unlikely to fall below stated cost targets. *One Earth*, 6(7):899–917, 2023. DOI: 10.1016/j.oneear.2023.06.004.

[122] Technology Data - Carbon Capture, Transport and Storage: Technology description and projections for long-term energy system planning, 2024. URL https://ens.dk/en/our-services/technology-catalogues/technology-data-carbon-capture-transport-and-storage, [Online] (accessed on 2024-09-23).

[123] U.S. Department of Energy. Technical Targets for Proton Exchange Membrane Electrolysis, 2023. URL https://www.energy.gov/eere/fuelcells/technical-targets-proton-exchange-membrane-electrolysis, [Online] (accessed on 2023-10-15).

[124] Y. Qiu, S. Cohen, and S. Suh. Decarbonization scenarios of the U.S. Electricity system and their costs. *Applied Energy*, 325(6064):119679, 2022. DOI: 10.1016/j.apenergy.2022.119679.

[125] A. Gonzalez-Garay and G. Guillén-Gosálbez. SUSCAPE: A framework for the optimal design of SUStainable ChemicAl ProcEsses incorporating data envelopment analysis. *Chemical Engineering Research and Design*, 137:246–264, 2018. DOI: 10.1016/j.cherd.2018.07.009.

[126] Producer Price Index by Industry: Chemical Manufacturing: PCU325325, retrieved from FRED, Federal Reserve Bank of St. Louis, 2023. URL https://fred.stlouisfed.org/series/PCU325325, [Online] (accessed on 2023-08-15).

[127] Euro to US Dollar Spot Exchange Rates for 2018: Average exchange rate in 2018, 2023. URL https://www.exchangerates.org.uk/EUR-USD-spot-exchange-rates-history-2018.html, [Online] (accessed on 2023-08-16).

[128] Intratec Solutions. Butene Price Outlook — Q4 2022, 2023. URL https://medium.com/intratec-products-blog/butene-prices-latest-historical-data-in-several-countries-3e435ef7daa9, [Online] (accessed on 2024-01-23).

[129] R. G. Grim, J. R. Ferrell III, Z. Huang, L. Tao, and M. G. Resch. The feasibility of direct CO_2 conversion technologies on impacting mid-century climate goals. *Joule*, 7 (8):1684–1699, 2023. DOI: 10.1016/j.joule.2023.07.008.

[130] H. Vatankhah Ghadim, J. Haas, C. Breyer, H. C. Gils, P. Odonkor, E. G. Read, M. Xiao, and R. Peer. Clean technology cost projections: investment and levelized costs of solar, wind, battery, and hydrogen. *Scientific Data*, 12(1):1670, 2025. DOI: 10.1038/s41597-025-05951-4.

[131] Electricity prices, 2026. URL `https://www.globalpetrolprices.com/electricity_prices/`, [Online] (accessed on 2026-05-17).

[132] K. Sievert, T. S. Schmidt, and B. Steffen. Considering technology characteristics to project future costs of direct air capture. *Joule*, 8(4):979–999, 2024. DOI: 10.1016/j.joule.2024.02.005.

[133] DIN EN ISO 14040 International Standard: Environmental Management - Life Cycle Assessment - Principles and Framework, 2009.

[134] DIN EN ISO 14044 International Standard: Environmental Management - Life Cycle Assessment - Requirements and Guidelines, 2006.

[135] K. Madhu, S. Pauliuk, S. Dhathri, and F. Creutzig. Understanding environmental trade-offs and resource demand of direct air capture technologies through comparative life-cycle assessment. *Nature Energy*, 6(11):1035–1044, 2021. DOI: 10.1038/s41560-021-00922-6.

[136] P. Gagnon, A. Pham, W. Cole, S. Awara, A. Barlas, M. Brown, P. Brown, V. Carag, S. Cohen, A. Hamilton, J. Ho, S. Inskeep, A. Karmakar, L. Lavin, A. Lopez, T. Mai, J. Mowers, M. Mowers, C. Murphy, P. Pinchuk, A. Schleifer, B. Sergi, D. Steinberg, and T. Williams. 2023 Standard Scenarios Report: A U.S. Electricity Sector Outlook: NREL/TP-6A40-87724, 2023. URL `https://www.nrel.gov/docs/fy24osti/87724.pdf`, [Online] (accessed on 2025-10-22).

[137] G. Valencia Ochoa, J. Cárdenas Gutierrez, and J. Duarte Forero. Exergy, Economic, and Life-Cycle Assessment of ORC System for Waste Heat Recovery in a Natural Gas Internal Combustion Engine. *Resources*, 9(1):2, 2020. DOI: 10.3390/resources9010002.

[138] L. Gerber, M. Gassner, and F. Maréchal. Systematic integration of LCA in process systems design: Application to combined fuel and electricity production from ligno-

cellulosic biomass. *Computers & Chemical Engineering*, 35(7):1265–1280, 2011. DOI: 10.1016/j.compchemeng.2010.11.012.

[139] Shell and Coil Heat Exchangers, 2022. URL `https://hexonic.com/wp-content/uploads/2021/11/HEXONIC_HAD_EN_ver.1.2022.pdf`, [Online] (accessed on 2022-10-23).

[140] Shell Tube Heat Exchanger, 2016. URL `https://www.suntech-metals.com/supplier-105986-shell-tube-heat-exchanger`, [Online] (accessed on 2023-04-05).

[141] Shell-and-Tube Heat Exchangers 30-Series: Models 218, 525, and 1000, 2015. URL `https://www.bannerindustries.com/wp-content/uploads/2015/02/AMETEK-Shell-and-Tube-Heat-Exchangers-30-Series-10-Inch-Shell.pdf`, [Online] (accessed on 2022-10-23).

[142] S. Peng, T. Li, M. Dong, J. Shi, and H. Zhang. Life cycle assessment of a large-scale centrifugal compressor: A case study in China. *Journal of Cleaner Production*, 139: 810–820, 2016. DOI: 10.1016/j.jclepro.2016.08.105.

[143] M. Löffelholz, J. Weidner, J. Hartmann, H. Ostovari, J. Osiewacz, S. Engbers, B. Ellendorff, J. R. Junqueira, K. Weichert, N. von der Assen, W. Schuhmann, and T. Turek. Optimized scalable CuB catalyst with promising carbon footprint for the electrochemical CO_2 reduction to ethylene. *Sustainable Chemistry for Climate Action*, 3:100035, 2023. DOI: 10.1016/j.scca.2023.100035.

[144] A. Schreiber, A. Peschel, B. Hentschel, and P. Zapp. Life Cycle Assessment of Power-to-Syngas: Comparing High Temperature Co-Electrolysis and Steam Methane Reforming. *Frontiers in Energy Research*, 8:533850, 2020. DOI: 10.3389/fenrg.2020.533850.

[145] M. Rumayor, A. Dominguez-Ramos, and A. Irabien. Environmental and economic assessment of the formic acid electrochemical manufacture using carbon dioxide: Influence of the electrode lifetime. *Sustainable Production and Consumption*, 18:72–82, 2019. DOI: 10.1016/j.spc.2018.12.002.

[146] M. Mori, D. Iribarren, J. Cren, E. Cor, A. Lotrič, J. Gramc, B. Drobnič, L. Rey, F. Campos-Carriedo, G. Puig-Samper, E. Bargiacchi, J. Dufour, and R. Stropnik. Life cycle sustainability assessment of a proton exchange membrane fuel cell technology for ecodesign purposes. *International Journal of Hydrogen Energy*, 48(99):39673–39689, 2023. DOI: 10.1016/j.ijhydene.2023.05.255.

[147] R. Stropnik, A. Lotrič, A. Bernad Montenegro, M. Sekavčnik, and M. Mori. Critical materials in PEMFC systems and a LCA analysis for the potential reduction of environmental impacts with EoL strategies. *Energy Science & Engineering*, 7(6):2519–2539, 2019. DOI: 10.1002/ese3.441.

[148] D. A. Notter, K. Kouravelou, T. Karachalios, M. K. Daletou, and N. T. Haberland. Life cycle assessment of PEM FC applications: electric mobility and μ-CHP. *Energy & Environmental Science*, 8(7):1969–1985, 2015. DOI: 10.1039/C5EE01082A.

[149] G. Giacoppo, S. Hovland, and O. Barbera. 2 kW Modular PEM fuel cell stack for space applications: Development and test for operation under relevant conditions. *Applied Energy*, 242:1683–1696, 2019. DOI: 10.1016/j.apenergy.2019.03.188.

[150] D. W. Engel, A. C. Dalton, K. K. Anderson, C. Sivaramakrishnan, and C. Lansing. Development of Technology Readiness Level (TRL) Metrics and Risk Measures, 2012. URL `https://www.pnnl.gov/main/publications/external/technical_reports/PNNL-21737.pdf`, [Online] (accessed on 2023-12-02).

[151] Y. Qiu, P. Lamers, V. Daioglou, N. McQueen, H.-S. de Boer, M. Harmsen, J. Wilcox, A. Bardow, and S. Suh. Environmental trade-offs of direct air capture technologies in climate change mitigation toward 2100. *Nature Communications*, 13(1):3635, 2022. DOI: 10.1038/s41467-022-31146-1.

[152] J. Friedmann, Z. Fan, Z. Byrum, E. Ochu, A. Bhardwaj, and H. Sheerazi. Levelized Cost of Carbon Abatement: An Improved Cost-Assessment Methodology for a Net-Zero Emissions World, 2020. URL `https://www.energypolicy.columbia.edu/wp-content/uploads/2020/10/LCCA_CGEP-Report_111522.pdf`, [Online] (accessed on 2024-05-17).

[153] P. R. Shukla, J. Skea, R. Slade, A. Al Khourdajie, R. van Diemen, D. McCollum, M. Pathak, S. Some, P. Vyas, R. Fradera, M. Belkacemi, A. Hasija, G. Lisboa, S. Luz, and J. Malley. Climate Change 2022: Mitigation of Climate Change. Contribution of Working Group III to the Sixth Assessment Report of the Intergovernmental Panel on Climate Change, 2022. URL `https://www.ipcc.ch/report/ar6/wg3/`, [Online] (accessed on 2023-12-02).

[154] J. Martin, E. Dimanchev, and A. Neumann. Carbon abatement costs for renewable fuels in hard-to-abate transport sectors. *Advances in Applied Energy*, 12:100156, 2023. DOI: 10.1016/j.adapen.2023.100156.

[155] PEP Yearbook: Process Economics, 2020 Q2 data, 2020. URL https://www.spglobal.com/energy/en/products-solutions/chemicals/chemical-process-economics-program-pep, [Online] (accessed on 2026-04-02).

[156] P. Nuss and M. J. Eckelman. Life Cycle Assessment of Metals: A Scientific Synthesis. *PLoS ONE*, 9(7):e101298, 2014. DOI: 10.1371/journal.pone.0101298.

[157] Energy and Climate Protection, 2024. URL https://report.basf.com/2023/en/combined-managements-report/environmental-social-governance/environmental/energy-and-climate-protection.html, [Online] (accessed on 2025-11-04).

[158] Welcome to your CDP Climate Change Questionnaire 2023, 2023. URL https://corporate.dow.com/content/dam/corp/documents/about/066-00450-01-2023-cdp-report-climate-change.pdf, [Online] (accessed on 2025-11-04).

[159] Annual Report 2023: Transform to Perform, 2024. URL https://report.covestro.com/annual-report-2023/_assets/downloads/covestro-ar23-entire.pdf, [Online] (accessed on 2025-11-04).

[160] Direct electrification of industrial process heat: An assessment of technologies, potentials and future prospects for the EU: Study on behalf of Agora Industry, 2024. URL https://www.agora-industry.org/fileadmin/Projects/2023/2023-20_IND_Electrification_Industrial_Heat/A-IND_329_04_Electrification_Industrial_Heat_WEB.pdf, [Online] (accessed on 2025-11-04).

[161] Climate Change and Resource Efficiency, 2023. URL https://www.sabic.com/en/reports/integrated-report-2023/value-chain-impact/production-ccre, [Online] (accessed on 2025-11-04).

[162] A. Al Moinee, A. A. Rownaghi, and F. Rezaei. Process Development and Techno-Economic Analysis for Combined and Separated CO_2 Capture-Electrochemical Utilization. *Chemical Engineering Journal*, 499:155909, 2024. DOI: 10.1016/j.cej.2024.155909.

[163] F. K. Bahnamiri, M. Khalili, P. Pakzad, and M. Mehrpooya. Techno-economic assessment of a novel power-to-liquid system for synthesis of formic acid and ammonia, based on CO_2 electroreduction and alkaline water electrolysis cells. *Renewable Energy*, 187:1224–1240, 2022. DOI: 10.1016/j.renene.2022.01.085.

[164] Z. Huang, R. G. Grim, J. A. Schaidle, and L. Tao. The economic outlook for converting CO_2 and electrons to molecules. *Energy & Environmental Science*, 14(7):3664–3678, 2021. DOI: 10.1039/D0EE03525D.

[165] A. Silveira Sbrice Pinto, N. Gulpinar, F. Liu, E. A. Gibson, L. Fuller, and P. Souter. Designing the Ethylene Factory for Products of Carbon Dioxide Reduction: Techno-Economic and Life Cycle Assessments. *ACS Sustainable Chemistry & Engineering*, 13 (37):15257–15268, 2025. DOI: 10.1021/acssuschemeng.4c10485.

[166] G. Glenk and S. Reichelstein. Economics of converting renewable power to hydrogen. *Nature Energy*, 4(3):216–222, 2019. DOI: 10.1038/s41560-019-0326-1.

[167] S. Krishnan, V. Koning, M. Theodorus de Groot, A. de Groot, P. G. Mendoza, M. Junginger, and G. J. Kramer. Present and future cost of alkaline and PEM electrolyser stacks. *International Journal of Hydrogen Energy*, 48(83):32313–32330, 2023. DOI: 10.1016/j.ijhydene.2023.05.031.

[168] W. Deng, A. Lee, W. Dai, L. Cherniack, B. S. Crandall, H. Li, and F. Jiao. Techno-economics of polymer-membrane-based CO_2 electrolysers. *Nature Reviews Clean Technology*, 1(4):255–268, 2025. DOI: 10.1038/s44359-025-00045-1.

[169] G. P. Towler and R. K. Sinnott. *Chemical engineering design: Principles, practice, and economics of plant and process design*. Butterworth-Heinemann, Boston, MA, USA, 2nd edition, 2012.

[170] M. S. Peters, K. D. Timmerhaus, and R. E. West. *Plant design and economics for chemical engineers*. McGraw-Hill chemical engineering series. McGraw-Hill, Boston, MA, USA and London, UK, 5th edition, 2003.

[171] T. Terlouw, C. Bauer, R. McKenna, and M. Mazzotti. Large-scale hydrogen production via water electrolysis: a techno-economic and environmental assessment. *Energy & Environmental Science*, 15(9):3583–3602, 2022. DOI: 10.1039/D2EE01023B.

[172] B. Belsa, L. Xia, V. Golovanova, B. Polesso, A. Pinilla-Sánchez, L. San Martín, J. Ye, C.-T. Dinh, and F. P. García de Arquer. Materials challenges on the path to gigatonne CO_2 electrolysis. *Nature Reviews Materials*, 9(8):535–549, 2024. DOI: 10.1038/s41578-024-00696-9.

[173] B. Parkinson, M. Tabatabaei, D. C. Upham, B. Ballinger, C. Greig, S. Smart, and E. McFarland. Hydrogen production using methane: Techno-economics of decarbonizing

fuels and chemicals. *International Journal of Hydrogen Energy*, 43(5):2540–2555, 2018. DOI: 10.1016/j.ijhydene.2017.12.081.

[174] A. Badgett, J. Brauch, A. Thatte, R. Rubin, C. Skangos, X. Wang, R. Ahluwalia, B. Pivovar, and M. Ruth. Updated Manufactured Cost Analysis for Proton Exchange Membrane Water Electrolyzers, 2024. URL `https://www.nrel.gov/docs/fy24osti/87625.pdf`, [Online] (accessed on 2024-03-07).

[175] G. Gao, C. A. Obasanjo, J. Crane, and C.-T. Dinh. Comparative analysis of electrolyzers for electrochemical carbon dioxide conversion. *Catalysis Today*, 423:114284, 2023. DOI: 10.1016/j.cattod.2023.114284.

[176] L. Berkelaar, J. van der Linde, J. Peper, A. Rajhans, D. Tiemessen, L. van der Ham, and H. den van Berg. Electrochemical conversion of carbon dioxide to ethylene: Plant design, evaluation and prospects for the future. *Chemical Engineering Research and Design*, 182:194–206, 2022. DOI: 10.1016/j.cherd.2022.03.034.

[177] C. Xu, P. Hong, Y. Dong, M. Robert, G. Shao, and Y. Lei. Toward Complete CO_2 Electroconversion: Status, Challenges, and Perspectives. *Advanced Energy Materials*, 16(8):2406146, 2026. DOI: 10.1002/aenm.202406146.

[178] S. A. Hawks, V. M. Ehlinger, T. Moore, E. B. Duoss, V. A. Beck, A. Z. Weber, and S. E. Baker. Analyzing Production Rate and Carbon Utilization Trade-offs in CO2RR Electrolyzers. *ACS Energy Letters*, 7(8):2685–2693, 2022. DOI: 10.1021/acsenergylett.2c01106.

[179] S. Y. Chae, S. Y. Lee, S. G. Han, H. Kim, J. Ko, S. Park, O.-S. Joo, D. Kim, Y. Kang, U. Lee, Y. J. Hwang, and B. K. Min. A perspective on practical solar to carbon monoxide production devices with economic evaluation. *Sustainable Energy & Fuels*, 4 (1):199–212, 2020. DOI: 10.1039/C9SE00647H.

[180] T. Janković, A. J. Straathof, I. R. McGregor, and A. A. Kiss. Bioethanol separation by a new pass-through distillation process. *Separation and Purification Technology*, 336: 126292, 2024. DOI: 10.1016/j.seppur.2024.126292.

[181] L. M. Vane. Separation technologies for the recovery and dehydration of alcohols from fermentation broths. *Biofuels, Bioproducts and Biorefining*, 2(6):553–588, 2008. DOI: 10.1002/bbb.108.

[182] S. Heuser, L. Hoof, K. Pellumbi, J. N. Oberndorf, L. Krämer, D. Blaudszun, K. j. Puring, M. Prokein, N. Mölders, A. Kilzer, M. Petermann, and U.-P. Apfel. Differential

pressure CO_2 electrolysis opens the way for direct coupling to industrial processes. *Chem Catalysis*, 575:101393, 2025. DOI: 10.1016/j.checat.2025.101393.

[183] Carbon dioxide: NIST Chemistry WebBook, 2025. URL `https://webbook.nist.gov/cgi/cbook.cgi?ID=C124389`, [Online] (accessed on 2025-06-11).

[184] S. Jackson and E. Brodal. A comparison of the energy consumption for CO_2 compression process alternatives. *IOP Conference Series: Earth and Environmental Science*, 167:012031, 2018. DOI: 10.1088/1755-1315/167/1/012031.

[185] L. Huang, G. Gao, C. Yang, X.-Y. Li, R. K. Miao, Y. Xue, K. Xie, P. Ou, C. T. Yavuz, Y. Han, G. Magnotti, D. Sinton, E. H. Sargent, and X. Lu. Pressure dependence in aqueous-based electrochemical CO_2 reduction. *Nature Communications*, 14(1):2958, 2023. DOI: 10.1038/s41467-023-38775-0.

[186] R. Sun, J. Zhao, H. Liu, Y. Xue, and X. Lu. Pressure regulated CO_2 electrolysis on two-dimensional Bi_2O_2Se. *Chemical Communications (Cambridge, England)*, 61(10): 2071–2074, 2025. DOI: 10.1039/d4cc05357e.

[187] M. G. Lee, S. Kandambeth, X.-Y. Li, O. Shekhah, A. Ozden, J. Wicks, P. Ou, S. Wang, R. Dorakhan, S. Park, P. M. Bhatt, V. S. Kale, D. Sinton, M. Eddaoudi, and E. H. Sargent. Bimetallic Metal Sites in Metal-Organic Frameworks Facilitate the Production of 1-Butene from Electrosynthesized Ethylene. *Journal of the American Chemical Society*, 146(20):14267–14277, 2024. DOI: 10.1021/jacs.4c03806.

[188] W. H. Lee, C. Lim, S. Y. Lee, K. H. Chae, C. H. Choi, U. Lee, B. K. Min, Y. J. Hwang, and H.-S. Oh. Highly selective and stackable electrode design for gaseous CO_2 electroreduction to ethylene in a zero-gap configuration. *Nano Energy*, 84:105859, 2021. DOI: 10.1016/j.nanoen.2021.105859.

[189] C. A. Giron Rodriguez, N. C. Kani, A. B. Moss, B. O. Joensen, S. Garg, W. Deng, T. Wilson, J. R. Varcoe, I. Chorkendorff, and B. Seger. Insights into zero-gap CO_2 electrolysis at elevated temperatures. *EES Catalysis*, 2(3):850–861, 2024. DOI: 10.1039/D3EY00224A.

[190] V. D. Brandão, H. Song, A. Venkataraman, Y. Fishler, S. S. Arora, S. S. Bhargava, C. Villa, A. Holewinski, S. Nair, M. C. Hatzell, and C. Sievers. Temperature Effects on the Surface CO Population during CO_2 Electroreduction over Copper. *ACS Catalysis*, 15(11):8979–8990, 2025. DOI: 10.1021/acscatal.5c01173.

[191] Q. Ren, H. Hua, and J. Luo. Temperature impact on zero-gap CO_2 electrolyzers. *Cell Reports Physical Science*, 6(9):102802, 2025. DOI: 10.1016/j.xcrp.2025.102802.

[192] A. Gawel, T. Jaster, D. Siegmund, J. Holzmann, H. Lohmann, E. Klemm, and U.-P. Apfel. Electrochemical CO_2 reduction - The macroscopic world of electrode design, reactor concepts & economic aspects. *iScience*, 25(4):104011, 2022. DOI: 10.1016/j.isci.2022.104011.

[193] S. J. Blazer, Y. Wang, N. Xu, X.-D. Zhou, and B. Marchetti. A systematic life cycle assessment of the electroconversion of carbon dioxide. *Sustainable Energy Technologies and Assessments*, 61:103574, 2024. DOI: 10.1016/j.seta.2023.103574.

[194] W. Fang, W. Guo, R. Lu, Y. Yan, X. Liu, D. Wu, F. M. Li, Y. Zhou, C. He, C. Xia, H. Niu, S. Wang, Y. Liu, Y. Mao, C. Zhang, B. You, Y. Pang, L. Duan, X. Yang, F. Song, T. Zhai, G. Wang, X. Guo, B. Tan, T. Yao, Z. Wang, and B. Y. Xia. Durable CO_2 conversion in the proton-exchange membrane system. *Nature*, 626(7997):86–91, 2024. DOI: 10.1038/s41586-023-06917-5.

[195] C. D. Sewell, D. A. Henckel, and M. G. Resch. Ion-Exchange Membrane-Centric Durability Testing and Degradation Characterization for Industry-Relevant CO_2 Reduction. *Industrial & Engineering Chemistry Research*, 64(43):20431–20440, 2025. DOI: 10.1021/acs.iecr.5c02103.

[196] A. Mayyas, M. Ruth, B. Pivovar, G. Bender, and K. Wipke. Manufacturing Cost Analysis for Proton Exchange Membrane Water Electrolyzers: NREL/TP-6A20-72740, 2019. URL `https://docs.nlr.gov/docs/fy19osti/72740.pdf`, [Online] (accessed on 2026-05-31).

[197] A. Ozden. Pathways to feasible hydrogen production in alkaline water electrolyzers. *Energy Nexus*, 21(1):100620, 2026. DOI: 10.1016/j.nexus.2025.100620.

[198] H. F. Araújo, J. A. Gómez, and D. M. F. Santos. Proton-Exchange Membrane Electrolysis for Green Hydrogen Production: Fundamentals, Cost Breakdown, and Strategies to Minimize Platinum-Group Metal Content in Hydrogen Evolution Reaction Electrocatalysts. *Catalysts*, 14(12):845, 2024. DOI: 10.3390/catal14120845.

[199] B. Kumar, B. Muchharla, M. Dikshit, S. Dongare, K. Kumar, B. Gurkan, and J. M. Spurgeon. Electrochemical CO_2 Conversion Commercialization Pathways: A Concise Review on Experimental Frontiers and Technoeconomic Analysis. *Environmental Science & Technology Letters*, 11(11):1161–1174, 2024. DOI: 10.1021/acs.estlett.4c00564.

[200] H. Zheng, K. Shi, H. Dong, Y. Yang, P. Yin, B. Shen, and J. Wang. Electrochemical Reduction of CO_2 to Multicarbon Products: A Review on Catalysts and System Optimization toward Industrialization. *Energy & Fuels*, 39(20):9316–9344, 2025. DOI: 10.1021/acs.energyfuels.5c00893.

[201] D. Obi, S. Onyekuru, and A. Orga. Review of recent process developments in the field of carbon dioxide (CO_2) capture from power plants flue gases and the future perspectives. *International Journal of Sustainable Energy*, 43(1):34, 2024. DOI: 10.1080/14786451.2024.2317137.

[202] H. Esen, M. Inalli, M. Esen, and K. Pihtili. Energy and exergy analysis of a ground-coupled heat pump system with two horizontal ground heat exchangers. *Building and Environment*, 42(10):3606–3615, 2007. DOI: 10.1016/j.buildenv.2006.10.014.

[203] O. Ozgener and A. Hepbasli. Experimental performance analysis of a solar assisted ground-source heat pump greenhouse heating system. *Energy and Buildings*, 37(1): 101–110, 2005. DOI: 10.1016/j.enbuild.2004.06.003.

[204] F. M. Geilen, G. Stochniol, S. Peitz, E. Schulte-Koerne, and E. Schulte-Koerne. Butenes. In *Ullmann's Encyclopedia of Industrial Chemistry*, volume 14, pages 1–13. Wiley, Weinheim, Germany, 2013. DOI: 10.1002/14356007.a04_483.pub3.

[205] J. Gao, S. Choo Sze Shiong, and Y. Liu. Reduction of CO_2 to chemicals and Fuels: Thermocatalysis versus electrocatalysis. *Chemical Engineering Journal*, 472(11): 145033, 2023. DOI: 10.1016/j.cej.2023.145033.

[206] R. Meys, A. Kätelhön, M. Bachmann, B. Winter, C. Zibunas, S. Suh, and A. Bardow. Achieving net-zero greenhouse gas emission plastics by a circular carbon economy. *Science*, 374(6563):71–76, 2021. DOI: 10.1126/science.abg9853.
